# The preventive/therapeutic effect of CO_2_ laser and MI Paste Plus® on intact and demineralized enamel against *Streptococcus mutans* (In Vitro Study)

**DOI:** 10.1016/j.heliyon.2023.e20310

**Published:** 2023-09-23

**Authors:** Dhuha H. Almarsomy, Fadia A. Al-khayat, Lamis A. Al-Taee

**Affiliations:** a Department of Conservative and Aesthetic Dentistry, Baghdad College of Dentistry, University of Baghdad, Baghdad, Iraq; b Department of Basic Sciences, Baghdad College of Dentistry, University of Baghdad, Baghdad, Iraq

**Keywords:** CO_2_ laser, MI Paste Plus®, Demineralized enamel, *Streptococcus mutans*, Microhardness

## Abstract

**Background:**

To evaluate the preventive and therapeutic effects of CO_2_ laser and MI paste plus on intact and demineralized enamel surfaces and their impact on bacterial adhesion. Methods: 160 enamel slabs were prepared and randomly allocated into two main groups; sound and demineralized enamel (n = 80 per group), in which specimens were immersed in a demineralizing solution (50 mM acetic acid, pH 4.5) for 72 h at 37 °C. Each group was further divided into four subgroups (n = 20); the control (un treated surfaces), surfaces treated by CO_2_ laser, MI paste plus (Recaldent™, GC corporation/Germany), and those received a combination of CO_2_ and MI paste plus. *Streptococcus Mutans* biofilm was isolated, quantified, and then applied on treated enamel surfaces and incubated anaerobically for 24 h and then quantified by colony-forming unit (CFU). Meanwhile, surface changes were assessed by Vickers microhardness and Scanning Electron Microscope combined with Energy-Dispersive X-Ray Spectroscopy (SEM-EDX). Results: The combined use of CO_2_ laser followed by MI paste plus significantly (p < 0.000) enhanced surface microhardness of sound and demineralized enamel with a significant reduction in bacterial counts. However, each technique alone was beneficial as they exhibited higher microhardness with lower bacterial viability in comparison to the control. The treatment of demineralized enamel surfaces with MI paste significantly reduced the number of bacterial colonies with the presence of dispersed mineral deposits over the surface.

**Conclusions:**

The combined use of CO_2_ laser and MI paste plus was effective as a preventive and/or therapeutic measures in enhancing surface properties of enamel and reducing the bacterial viability.

## 1 Introduction

Dental caries is a pandemic oral disease with a high prevalence socioeconomically based on epidemiological evidence [1]. The previous approaches were based on the use of operative intervention to limit caries progression and recurrence. However, recently, the focus is toward implementing non‐invasive techniques to preserve the integrity of tooth structure against decay and arrest initial carious lesions. Dental caries is initiated when there is an imbalance in the demineralization-remineralization process, once the demineralization proceeds, a net mineral loss will happen which leads to caries development and progression [2]. However, if mineral re-deposition predominates, remineralization will arrest carious lesions. Several caries-preventive strategies are implemented to prevent and treat dental caries in clinical practice. Even though, they present some questionable aspects. The application of fluoride is an effective measure that was used to impede enamel demineralization and thus enhance remineralization even at low concentrations [3]. However, the antimicrobial capability of fluoride is inadequate to prevent the development of caries which requires the use of alternative prophylactic approach that prevent tooth decay via enhancing the structural properties of enamel with antibacterial properties [4].

MI Paste Plus is a commercial product composed of Casein phosphopeptide-amorphous calcium phosphate (CPP-ACPF) that has mineralizing and antimicrobial abilities. It is claimed to supply the lost ions in demineralized enamel [5,6], and affect the binding property of bacteria to tooth surface [7]. The anticariogenicity of CPP-ACPF paste was proved in randomised, controlled clinical trials [8,9]. However, limited studies were conducted to suggest whether this paste is efficient to enhance the surface properties of enamel and thereby increase the resistance to bacterial adhesion.

Laser irradiation is one of the preventive approaches that can enhance the resistance of enamel to acid dissolution and bacterial adhesion [10]. The application of carbon dioxide (CO_2_) laser at 10.6 μm wavelength is highly recommended, since it penetrates ten times deeper without damaging the surface nor causing an increase in pulp temperature [11,12]. Moreover, the absorption of this wavelength is close to the phosphate, carbonate, and hydroxyl groups in the hydroxyapatite [10,13]. Once the light absorbed by minerals, structural and chemical alterations in enamel crystals will occur manifested by thermal decomposition of the carbonated apatite which then reduces the solubility and thus enhances the resistance against acid attack [14,15]. The surface changes occur within the depth of 58 μm, which is sufficient to reduce the demineralization in enamel up to 98% [16]. However, the use of high energy of CO_2_ laser can lead to cracks and irregularities at the surface, which increase the brittleness and reduce tissue hardness [17]. Nevertheless, if appropriate parameters are selected, they can induce favorable changes within enamel structure and thus increasing its resistance to bacterial acids [18]. Accordingly, this study evaluated and compared the effectiveness of CO_2_ laser, MI Paste Plus, and both, in enhancing the surface properties of healthy and demineralized enamel against *streptococcus mutans* biofilm. The null hypothesis tested was none of these approaches is sufficient to enhance the surface properties of enamel nor affect the *SM* adhesion.

## 2 Materials and methods

### 2.1 Sample preparation

**Fig. 1:**
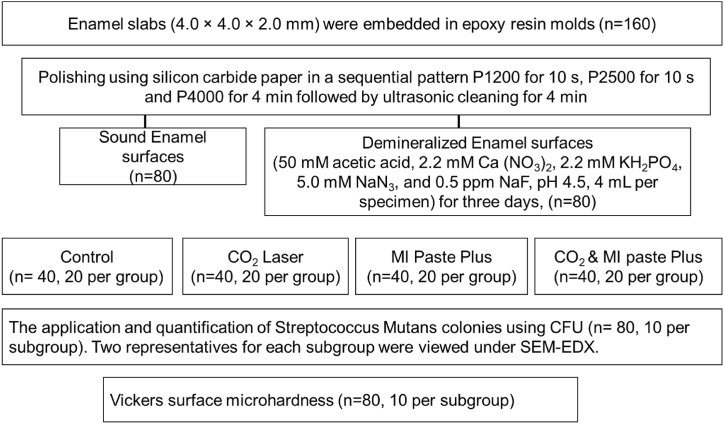
A scheme representing the experimental groups and procedures.

Forty permanent caries-free premolar teeth were selected from patients <20 yrs for orthodontic treatment using an ethics protocol approved by the health research committee (Ref No.496, January 19, 2022), then stored in de-ionized water in a cold cabinet (+4 °C). Teeth were randomly allocated in to two main groups; sound and demineralized enamel (n = 20 per group). The roots were sectioned at the cemento-enamel junction (Isomet 1000, Buehler, USA) using a water-cooled diamond blade (330-CA/RS-70300, Struers, Detroit, USA). Each crown was sectioned mesiodistally into two-halves obtaining the buccal half only, then each surface was further sectioned in to 4 slabs (4.0 × 4.0 × 2.0 mm) and embedded in epoxy resin molds. All specimens (n = 160) were polished (Laryee Technology CO.LTD, Beijing, China) under water cooling using silicon carbide paper in a sequential pattern P1200 for 10 s, P2500 for 10 s and P4000 for 4 min to obtain smooth and flat surfaces, followed by ultrasonic cleaning for 4 min to remove surface debris, then stored in deionized water [19]. In the first group (sound enamel), specimens were subdivided into four subgroups which received CO_2_ laser, MI Paste Plus, and a combination of CO_2_ laser and MI paste plus, with those left without treatment as a control. In the second group (n = 80, 20 per subgroup), specimens were treated similarly after being subjected to a demineralization process, Fig. 1.

### 2.2 Demineralized enamel lesions

The demineralized lesions were created on enamel surfaces in the second group following Fan et al. (2021) [20]. In which the enamel slabs were immersed in a demineralization solution (50 mM acetic acid, 2.2 mM Ca (NO_3_)_2_, 2.2 mM KH_2_PO_4_, 5.0 mM NaN_3_, and 0.5 ppm NaF, pH 4.5, 4 mL per specimen) for three days using a shaker (50 rpm/min) at 37 °C without refreshment, to induce subsurface demineralization mimicking an initial enamel lesion.

### 2.3 The application of MI paste PLUS

The MI Paste plus (RECALDENT™, GC, Breckerfeld, Germany) was applied on sound and demineralized enamel surfaces (n = 20 per group) following the manufactures’ instructions. In which a micro applicator brush was used to dispense the paste over enamel surface and left undisturbed for 3 min. The paste was spread over the surface for 1 min in a circular motion, then washed away after 5 min with deionized water for 30 s. This procedure was repeated daily for two weeks, and specimens were kept in artificial saliva at 37 °C.

### 2.4 The application of carbon dioxide laser CO_2_

A commercially pulsed CO_2_ laser (CO_2_ Fractional Laser, JHC1180, China) was applied to sound and demineralized enamel surfaces (n = 20 per group) using the following parameters: 2 W power, 10 ms pulse duration, 50 Hz pulse frequency, 0.2 mm focal spot, and 11.5 J/cm^2^ energy. Surface scanning was done for 5 s in non-contact mode keeping a 10 mm distance from the irradiated surfaces accompanied with water cooling to mimic the clinical condition and preserve pulp vitality [12]. In the last subgroups (n = 40), of both sound and demineralized enamel, the surfaces were exposed to CO_2_ laser then treated by MI paste for two weeks following the same procedures of both. Then, all specimens were kept in artificial saliva at 37 °C until the next step.

### 2.5 Plaque biofilm collection

Plaque samples were collected from five healthy individuals with no oral disease with an age range between 25 and 54 years after obtaining the informed consent form. A microbiological collection transport swab was used for collecting samples after drying the surface to prevent contamination with saliva. Then, each swab (n = 5) was immersed individually in 1 mL of sterile phosphate buffered saline immediately, then kept inside an icebox until being transferred to the laboratory.

### 2.6 Isolation, identification of *S. Mutans*

The collected samples were subjected to a vortex for a minute to disperse the plaque and obtain a homogeneous suspension. A ten-fold serial dilution of each sample was prepared up to the fifth dilution. Cultures were made from each dilution on a selective media for *S. mutans* (Mitis salivarius bacitracin agar, MSBA, Sigma-Aldrich Chemie GmbH, Eschenstrasse 5, D-82024 TAUFKIRCHEN) and incubated anaerobically for 48 h at 37°, followed by 24 h in aerobic condition [21]. The streptococcus colonies were identified using Gram's stain, which appeared under a light microscope as; round or spherical, dark blue, pin point size, and arranged in chains. Several biochemical tests were performed to identify the isolated species including; The Catalase test, Blood hemolysis test, Carbohydrate fermentation test (mannitol) and Vitek® 2 system (BIOMÉRIEUX, Durham, NC 27712, USA). To maintain the bacterial isolate, the selective colony was transferred from MSBA to 10 mL of sterile Brain heart infusion (BHI–B) and incubated for 24 h aerobically at 37 °C, then stored in an inoculated broth at −80 °C after adding 20% sterilized glycerol, until being used [22].

### 2.7 The application and evaluation of *Streptococcus mutans*

The bacterial colonies were activated by adding 0.1 mL of pure *Mutans Streptococci* isolate to 10 mL of sterile BHI–B (pH 7.0), and then incubated aerobically for 18 h [23]. The freshly grown bacterial suspension was suspended to 1.5 × 10^8^ colony forming unit (CFU)/mL using McFarland test standard with 0.5 turbidity. The concentration of bacterial suspensions was adjusted by spectrophotometer with an optical density of 600 nm (OD_600_) to match the turbidity of all suspensions to 0.5 McFarland test standard.

All enamel blocks (sound and demineralized, n = 80, 10 per subgroup) were sterilized in an autoclave at 121 °C at 15 pounds per square inch for 15 min. Then each block was placed individually in a sterilized screw-capped bottle containing 2-mL of the adjusted bacterial suspension (1.5 × 10^8^ CFU/mL) and incubated aerobically at 37 °C for 24 h to simulate the cariogenic challenge on enamel surfaces by *SM* [24]. Then each enamel slab was removed from the acrylic block and inserted in a tube contains 10 mL of phosphate buffer saline (pH 7.2), and stirred by a Vortex for 1 min to detach the bacteria from the surface. This suspension was further diluted to ten-fold, and 100 μL of each diluent was transferred to MSBA agar contains 1% sucrose, and incubated in 5% CO_2_ at 37 °C for 48 h. The quantitative evaluation of the viable bacteria was carried out using a colony forming unit. In which the number of visible colonies (CFU) on the agar plate, using the 2nd dilution, were multiplied by the dilution factor to provide the CFU/ml values [25].

### 2.8 Surface microhardness

The Hardness profile of sound and demineralized enamel surfaces (n = 80, 10 per subgroup) was examined by using Vickers microhardness tester (TH715, Obsnap Instruments Sdn Bhd, Selangor, Malaysia) using a diamond square-based pyramid diamond-shaped indenter with a 200 gf load for 15 s [26]. Three indentations were done at middle of each surface with 500 μm distance apart. The Vickers hardness number was recorded automatically using the manufacturer's software, in which the value was an average of three readings.

### 2.9 Morphological observation

Two representative specimens of each subgroup were observed under Scanning Electron Microscopy (SEM, Thermo Scientific™ Axia™ ChemiSEM, FEI Company) to evaluate the surface morphology and bacterial aggregation on enamel surfaces, this was combined with energy dispersive X-ray spectroscopy (EDX) to examine the mineral profile. The biofilms on enamel surfaces were fixed in 2% glutaraldehyde (Sigma, Buchs, Switzerland) overnight, then the surfaces were washed once using Phosphate buffer solution (PBS, pH 7.4) and sterile water for 10 min. This was followed by surface dehydration using graded concentrations of ethanol solution 30%, 50%, 70%, 90%, and 100% for 10 min each. After that, all specimens were carbon-coated and viewed under SEM-EDX, using an accelerating voltage of 30 kV, 2500× and 5000× magnification powers, and the working distance at 50 μm and 30 m, respectively. The experimental groups and procedures are shown in Fig. 1.

### 2.10 Statistical analysis

The calculation of sample size was performed using G power 3.0.10 (Program written by Franz-Faul, University of Kiel, Germany) with a power of 95%, alpha error of probability = 0.05. The effect size of Cohen's D was 1.998 (large effect size), considering that the sample number (n = 10) is higher than the calculated G power. The statistical analysis was done using SPSS software version 26 (IBM, USA). Shapiro-Wilk test was used to evaluate the normality of distribution. The data was further analyzed using One-way ANOVA followed by Tukey post-hoc multiple comparisons (p < 0.05). The independent *t*-test (Minitab 14, Minitab Inc., USA) was performed to assess the difference between sound and demineralized enamel surfaces (p < 0.05).

## 3 Results

### 3.1 Colony forming unit (CFU)

The CFU of all groups with statistical correlations were presented in Table (1). All values were normally distributed (p > 0.05), which were further analyzed by One-way ANOVA and Tukey multiple comparison tests that showed statistically significant differences among groups (p < 0.05). In both sound and demineralized enamel groups, surfaces treated by CO_2_ laser followed by MI paste plus showed the least amount of bacterial accumulation among all groups (p < 0.000). This was followed by surfaces treated by MI paste alone then CO_2_ laser which were significantly lower than the control (p < 0.000).

**Table 1:** Colony Forming Units (mean ± SD) in sound and demineralized enamel surfaces when treated by CO_2_ laser, MI paste plus, CO_2_ laser and MI paste plus in comparison to those left without treatment (control).

Groups (n = 10)	Sound Enamel	Demineralized Enamel
Control	20200 ± 1398.4^a^	25700 ± 1398.4^a^^
CO_2_ laser	17200 ± 1619.3	15700 ± 1337.5^
MI Paste Plus	14900 ± 1449.1	12300 ± 1767.0^
CO_2_ and MI paste Plus	588 ± 127.3	2600 ± 966.1^

(^) a statistically significant difference between sound and demineralized enamel surfaces (Independent T-Test p < 0.05).

a A statistically significant difference between the control and surface treated groups in sound and demineralized enamel (Tukey post-hoc test, at alpha level of 0.05).

By comparing the effect of surface treatments in sound vs. demineralized enamel surfaces, the bacterial accumulation was higher on sound enamel surfaces that were treated by CO_2_ laser and MI paste in comparison to demineralized enamel surfaces (p = 0.04, 0.002, respectively). Whilst, a reduced bacterial count was shown in sound enamel surfaces when treated by CO_2_ laser combined with MI paste and those left without treatment (p = 0.000)**.**

### 3.2 Vickers microhardness

**Fig. 2:**
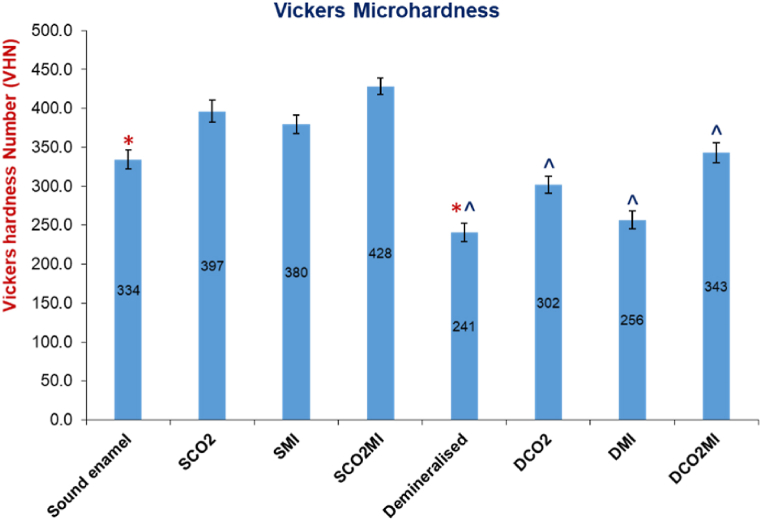
Means of Vickers microhardness number (VHN). Enamel surfaces treated by a combination of CO_2_ laser and MI paste plus recorded the highest VHN than other subgroups that were significantly higher than the control groups (p < 0.000), (*) A statistically significant difference between the control and surface treated groups in sound and demineralized enamel (Tukey post-hoc test, at alpha level of 0.05). (^) a statistically significant difference between sound and demineralized enamel surfaces within each group (Independent T-Test p < 0.05).

There was a statistically significant reduction in microhardness values of demineralized enamel surfaces in comparison to sound (Independent T-Test, p < 0.000). For the effect of surface treatments, in both groups, surfaces treated by a combination of CO_2_ laser and MI paste plus showed the highest statistically significant VHN among subgroups (p = 0.000). This is followed by CO_2_ irradiated surfaces, then those treated by MI paste plus alone (p < 0.000), while the untreated surfaces in both groups showed the lowest VHN (p < 0.000), Fig. 2.

### 3.3 Scanning electron microscopy-energy dispersive spectroscopy analysis

.

## 4 Discussion

This study evaluated and compared the preventive and therapeutic effects of CPP-ACP-contained paste (MI plus) and CO_2_ laser on sound and demineralized enamel against *Streptococcus Mutans* adhesion. The results suggested that the combined use of both approaches is effective in enhancing the hardness properties of sound and demineralized enamel surfaces associated with lower bacterial adhesion. Accordingly, the proposed hypothesis in the current study was rejected.

The surface layer of enamel plays a crucial role in the development and progression of dental caries initiated by microbial adhesion. Vickers microhardness is convenient test for the fine micro-structure and non-homogenous surfaces like tooth enamel, and it is sensitive to any alteration in the mineral density and considered as indirect evidence for mineral loss or gain [27].

In this study, the application of CO_2_ laser solely enhanced tissue hardness in sound and demineralized enamel when compared to non-treated surfaces and reduced the biofilm cultivation. The thermal effect of laser induces the formation of a hypercrystalline matrix by eliminating the impurities from the crystalline structure of HAp such as carbonate, sodium, and magnesium. The re-crystallization of HAp crystals enhances the resistance of enamel against bacterial acids [14]. This process decreases the permeability and hampers the diffusion of acids, thereby reducing the enamel demineralization [18,28].

**Fig. 3:**
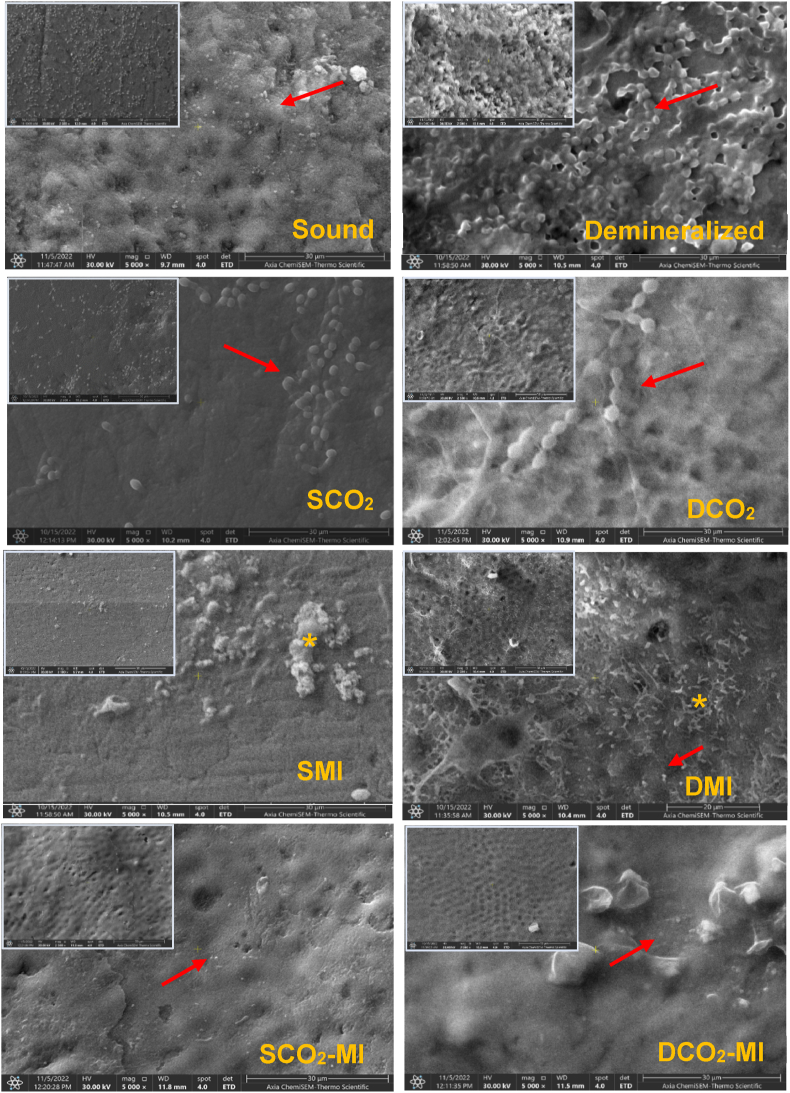
Scanning electron micrograph of bacterial aggregation on representative sound and demineralized enamel surfaces treated by various methods. A higher density of bacterial accumulations (red arrow) was observed on the demineralized enamel surface that left without treatment forming a continuous layer of bacteria adhered to the surface. The surface change (the melting effect) was clear in subgroups treated CO_2_ laser with less amount of *Streptococcus Mutans* on both subgroups (SCO_2_, DCO_2_) as compared to the control. The surfaces treated by MI paste (SMI, DMI) show the presence of mineral like deposits at the surface (yellow asterisks) with less amount of bacteria in comparison to the control and CO_2_-lased surfaces. The last subgroups that received both CO_2_ laser and MI paste (SCO_2_-MI, DCO_2_-MI) exhibited surface changes with few individual cells or small bacterial colonies dispersed onto the surfaces (red arrow).

Although the present study supported the caries-preventive effect of CO_2_ laser, it did not prevent the bacterial adhesion, since the count of viable *SM* was higher than other treated surfaces. The antimicrobial effect of CO_2_ laser was previously proven to be effective, but not for long-term caries prevention, since the bacteria will re-colonize on the tooth surface as part of the physiological oral environment. Nevertheless, laser-treated surfaces exhibited an enhanced tissue hardness associated with an altered morphology which results in a reduction in bacterial counts and density in comparison to non-treated enamel surfaces (Fig. 3 SCO_2_, and DCO_2_). This means that it may play a role in modulating biofilm formation and maturation, and subsequently prevent enamel caries [29]. The initial adhesion of bacteria on enamel is governed by van der Waals and electrostatic forces which rely on the physicochemical properties of the substrate such as; the free surface energy, hydrophobicity, and surface charge [24]. The photothermal effect of CO_2_ laser causes changes in the composition, and/or structure of enamel associated with phase transformation that reduces the water and carbonate contents with more structural OH- groups. This is directly affecting the solubility and diffusion of enamel [30]. On the other hand, the decrease in water results in an increase in the hydrophobicity of the substrate with higher OH- groups. This might reduce the adhesion force of the early strains to heated enamel surfaces up to 76% when measured by atomic force [30]. Song et al. (2015) [31], and Esteves-Oliveira et al. (2017) [12] supported the preventive effect of CO_2_ laser against biofilm-induced demineralization by reducing the adhesion of *SM* up to 79% in a highly cariogenic continuous-culture biofilm model compared to those left without treatment.

**Fig. 4:**
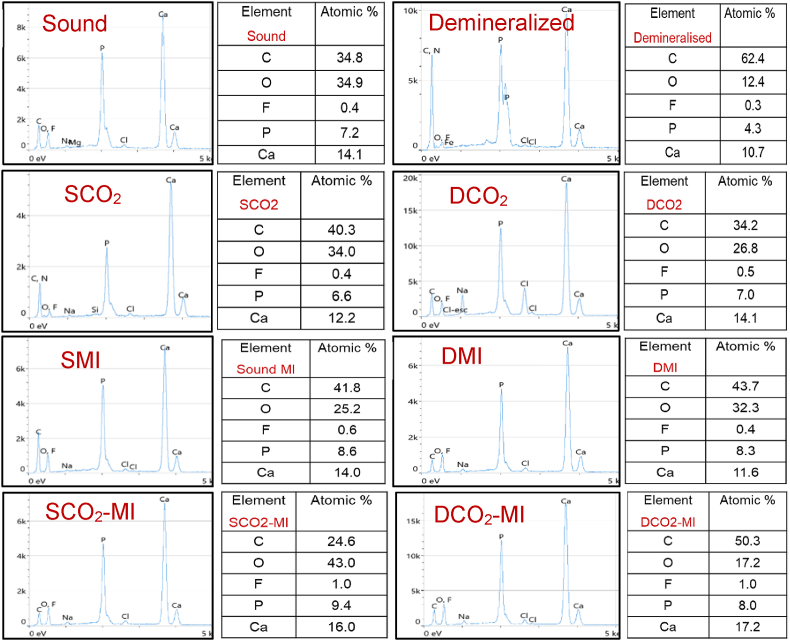
The EDX analysis of all groups (sound and demineralized) and subgroups (CO_2_ laser, MI paste plus, CO_2_-MI). The mineral components (calcium and phosphorus ions) are higher in sound and demineralized enamel surfaces treated by MI paste and a combination of CO_2_-MI paste.

Enamel surfaces treated by MI Paste Plus® showed an enhanced surface microhardness associated with a significant decrease in bacterial count when compared to laser-treated and non-treated surfaces. The anti-cariogenicity effect of this paste is referred to the calcium phosphate complexes of tryptic casein phosphopeptides [32] which expected to reduce the adherence of *SM* to enamel as compared to non-treated surfaces. This in consistent with Rose et al. (2000) [33] and Pukallus et al. (2013) [34] who found that the casein substitutes albumin in the pellicle interfere with the adhesion of *SM*, and provides basic amino acids that buffers the plaque acids and slow down the diffusion of free calcium ions restricting mineral loss during the cariogenic process and provide a potential source of calcium for subsequent remineralization. Moreover, the casein competes with calcium for plaque binding, which reduce the calcium-bridge between the surface and bacterial cells, and between the bacterial cells, which also reduce the adhesion of bacteria on enamel surfaces. The demineralized enamel surfaces treated by MI paste showed lower number of viable bacteria than sound enamel, which suggest the potential therapeutic effect of this paste that replaced the lost minerals from the surface illustrated by the presence of a random distribution of needle-like mineral deposits over the demineralized enamel surface under SEM (Fig. 3 DMI). In which the prismatic structure is slightly evident, but the cavities are well filled by minerals with a homogeneous and compact layer covering the demineralized enamel surface and dispersed bacterial colonies in comparison to the non-treated surfaces that showed a high degree of surface porosity with dense accumulations of bacteria colonizing over the whole surface (Fig. 3 D). This is inconsistent with previous studies [35,36] that supported the remineralization potential of CPP-ACP-contained products which allow deeper penetration of ions into enamel lesions, resulting in remineralization of entire body of the lesion rather than the superficial layer [36]. Additionally, the presence of fluoride (900 ppm) in MI plus paste is expected to form CPP-stabilized amorphous calcium fluoride phosphate and supply higher concentrations of calcium, phosphate, and fluoride ions thus promoting the remineralization of demineralized enamel surfaces [32]. The EDX analysis (Fig. 4 SMI, DMI) revealed a maintained calcium content (atomic %), but two-fold increase in phosphorous ions in comparison to non-treated demineralized surfaces. This might be due to the short demineralization period that was limited to 72 h which might not be enough to remove a significant level of calcium from enamel surface.

The best results achieved when the MI paste was applied on CO_2_ irradiated enamel surfaces. In which, enamel surfaces whether intact or demineralized showed significantly higher tissue hardness associated with significant reduction in *SM* count. This is attributed to the synergistic effect between CO_2_ laser and CPP-ACP-containing paste that are able to form both loosely and firmly bound mineral complexes [37]. The adsorption of these precipitates is a surface phenomenon and related to the surface energy. CO_2_ irradiation, at the same time, is known to modify tooth surface energy and the mechanical properties of the surface. Therefore, it can be speculated that the laser modified the surface energy for more stable adsorption of mineral complexes, than MI application alone. Additionally, the exposure to laser beams creates microcracks at enamel surfaces that are able to trap the available ions and serve as sites for remineralization within the enamel surface. This supported by EDX results that showed the higher contents of calcium, phosphate and fluoride in both enamel surfaces (Fig. 4, SCO_2_-MI, DCO_2_-MI). Moreover, CO_2_ laser could increase the uptake of fluoride by treated surfaces added to the consistency of the paste that allow more intimate contact with the surface, leading to elevated fluoride uptake by enamel surfaces. This might lead to the formation of a calcium fluoride-like material (CaF2-like) over the enamel surface [38], that leads to the transformation of HAp crystals into fluoroapatite. This justified the significant enhancement in microhardness of intact and demineralized enamel associated with a significant reduction in bacterial counts in comparison to all other groups (p < 0.000). However, the increase in temperature may affect the subsurface demineralized enamel surface which necessities further assessments.

This was supported by previous studies [39,40] that reported a significant increase in fluoride uptake when fluoride-containing products was combined with laser treatment. Nammour et al. (2003) [41] found that the in vivo application of argon laser allowed more fluoride retention compared to un-lased enamel associated with higher microhardness values than those of NaF mousse. Furthermore, laser irradiation induces changes in surface energy, charge, topography, with microcrack formation. These changes facilitate the adsorption of mineral precipitate when the CPP-ACP-contained paste was used for two weeks, which seems to have a direct impact on reducing the adhesion of *SM* biofilm [31].

The demineralized surfaces showed lower hardness values in comparison to sound enamel but higher resistance to biofilm adhesion especially when MI was used solely. This supports the potential repair ability of MI that supply the lost ions and form mineral complexes that reinforce the demineralized surfaces and provide better protection against biofilm formation [42].

This study utilized only monospecies biofilms, which may differ from the clinically relevant biofilms. However, the experimental studies suggested that the amount of lactic acid produced in vitro by polymicrobial biofilms is very similar to that produced by S. Mutans biofilms alone [43]. Moreover, the laser treatment was applied on smooth enamel surfaces, while in clinical reality, other surfaces are prone to caries lesions (i.e., proximal, adjacent to restoration) which cannot be treated using laser irradiation. Despite these limitations, the use of CO_2_ laser treatment alone or in combination with MI plus seems to be beneficial when applied in vitro using the described laser settings. Nevertheless, randomized clinical trials are required to justify the use of these promising approaches regarding tissue prevention and repair with clinical longevity.

## 5 Conclusions

The application of CO_2_ laser was successful in enhancing the surface characteristics of intact and demineralized enamel when followed by two weeks treatment of MI past plus with a significant reduction in viability of initial colonies of *SM*. This is associated with presence of a protective layer covering the demineralized enamel surface, with higher calcium, phosphorus and fluoride ions that are essential for subsequent remineralization. However, the use of each technique alone was also beneficial as they exhibited higher microhardness with lower CFU count in comparison to non-treated surfaces, with the presence of dispersed mineral deposits at enamel surfaces treated by MI paste plus.

## Author contribution statement

Dhuha H. Almarsomy: Conceived and designed the experiments; Analyzed and interpreted the data; Contributed reagents, materials, analysis tools or data; Wrote the paper.

Fadia A. Al-khayat: Performed the experiments; Analyzed and interpreted the data.

Lamis Abdul Hameed Al-Taee: Conceived and designed the experiments; Analyzed and interpreted the data; Wrote the paper.

## Funding statement

This research received no specific grant from any funding agency in the public, commercial, or not-for-profit sectors.

## Data availability statement

Data included in article/supp. Material/referenced in article.

## Declaration of competing interest

The authors declare that they have no known competing financial interests or personal relationships that could have appeared to influence the work reported in this paper.
