# Supplementary material for: The preventive/therapeutic effect of CO2 laser and MI Paste Plus® on intact and demineralized enamel against Streptococcus mutans (In Vitro Study)
Source: Heliyon. 2023 Sep 23;9(10):e20310. doi: 10.1016/j.heliyon.2023.e20310 (PMC10543189; doi:10.1016/j.heliyon.2023.e20310)
Supplement: Supplementary file 1 — Multimedia component 1 [file mmc1.docx]

**MATERIALS AND METHOD**

- 1. **Materials**
     1. **Materials for sample tooth collection**

The Materials used for sample tooth collection listed below and show in figure (2.1).

1. Forty freshly extracted of sound permanent upper first premolars.
2. Self-cure acrylic (Veracril® Acrílico Líquido Autopolimerizable) (EXP. Date 10-2024).
3. Alcohol 70%.
4. Cotton & gauze (Weiqiao Textile/China).
5. Disposable cups (Ruian Mingyuan Machinery/China).
6. Disposable Dental probe double ends (SinaliDent/China).
7. Face mask and shield (Xiantao Zhibo Nonwoven /China).
8. Sample beaker with cover 125ml
9. Bio-Shade Self Curing Denture Base Material Dental Acrylic Powder (EXP. Date 10-2024).
10. Deionized distilled water (Iraq), Exp. (05-2024).
11. Non-fluoride pumice powder (PD, Switzerland), Exp. (04-2024).
12. Rubber cup (Dental, china)
13. Silicon mold.
14. Dental bond brush.
15. Vaseline® Blue Seal Original Petroleum Jelly (EXP. Date 05-2023).
16. Surgical absorbent cotton (lagrita, Turkey), Exp. (03-2025).
17. Silicon carbide waterproof abrasive papers sequentially (P1200, P2500, and P4000) (Smirdex, Greece).

**Figure (2-1): Some of the materials used in the study.**


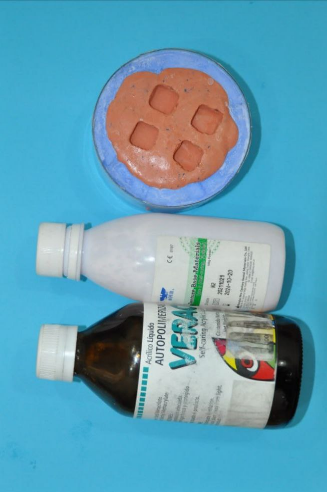

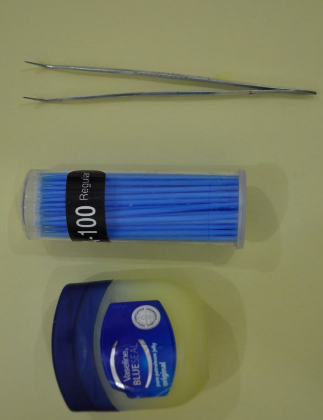

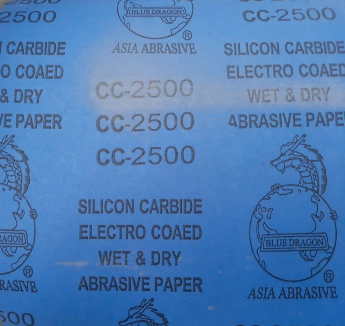

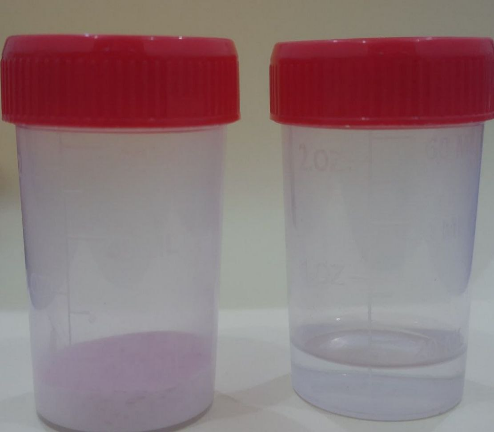

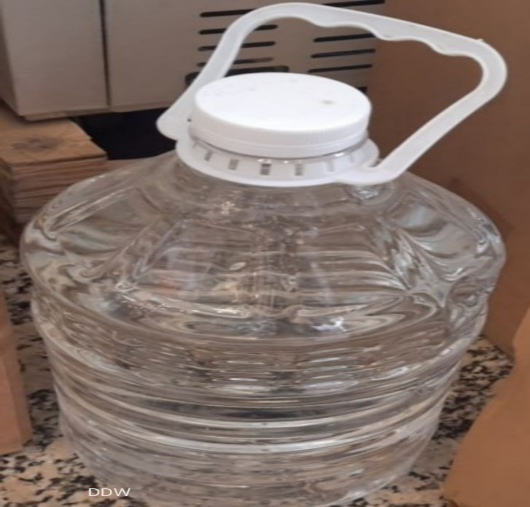

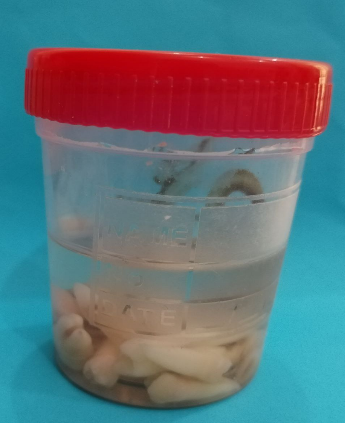


**2.1.2 Materials used in demineralized enamel lesions lesion formation.**

1. demineralization solution and their chemical composition that were used in this work are presented in table (2.1) and shown in figure (2.2)


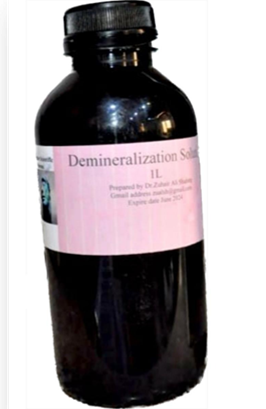


Figure 2.2: demineralization solution

| Table 2.1: The Formulations of Demineralizing solution | | | | |
| --- | --- | --- | --- | --- |
| Material | Formula | Concentration | Manufacturer | Exp |
| acetic acid, | CH _3_CO _2_H | 50 mM | Avonchem UK | 2024 |
| Calcium nitrate | Ca (NO_3_)_2_ | 2.2 mM | Hemedia india | 2023 |
| Potassium dihydrogen orthophosphate | KH_2_PO_4_ | 2.2 mM | Qualikemis india | 2023 |
| Sodium azide | NaN_3_ | 5.0 mM | Qualikemis india | 2023 |
| Sodium fluoride | NaF | 0.5 ppm | Hemedia india | 2023 |

**2.1.3**

**Materials used as remineralizing agents**

Materials used as remineralizing agents and their chemical composition that were used in this work are presented in table (2.2), (2.3) and shown in figure (2.3):


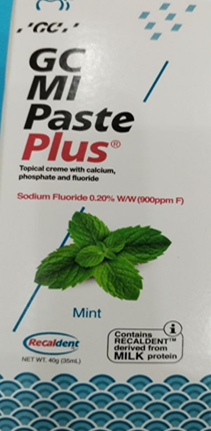

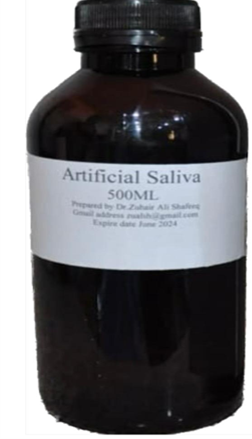


A B

Figure 2-3: Remineralizing agents’ materials (A: GC MI Paste Plus^®^, B: Artificial saliva)

| Table 2.2: The Formulations of remineralizing Agents | | | |
| --- | --- | --- | --- |
| Material | Manufacturer | Composition | Exp |
| GC MI Paste Plus^®^ | GC America INC.USA | Pure water, Glycerol, CPP_ACP, D-Sorbitol, CMC-Na, Propylene Glycol, Silicon Dioxide, Titanium Dioxide, Xylitol, Phosphoric Acid, Sodium Fluoride, Flavoring, Sodium Saccharin, Ethyl P-Hydroxybenzoate, Propyl P Hydroxybenzoate, Butyl P-Hydroxybenzoate. | 2-6-2023 |

| Table 2.3: The Formulations of Artificial saliva | | | | |
| --- | --- | --- | --- | --- |
| Material | Formula | Concentration | Manufacturer | Exp |
| Calcium chloride dehydrate | CaCl_2_ | 0.7 mmol/L | Avonchem UK | 2024 |
| Magnesium chloride | MgCl_2_.6H_2_O | 0.2 mmol/L | Thomas Baker india | 2024 |
| di-potassium hydrogen orthophosphate | KH2PO4 | 4 mmol/L | Thomas Baker india | 2024 |
| Potassium chloride | KCl, | 30 mmol/L | Alphachemika india | 2024 |
| 4-(2-hydroxyethyle)-piperazin-1-ethan-sulfonsaure | C_8_H_18_N_2_O_4_S | 20 mmol/L | Avonchem UK | 2026 |

**2.1.4 Materials used in plaque collection and microbiological part shown in the (table 2.4) and figure (2.4) below**


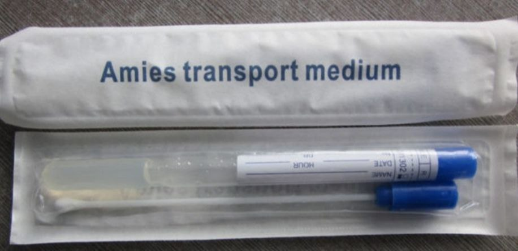

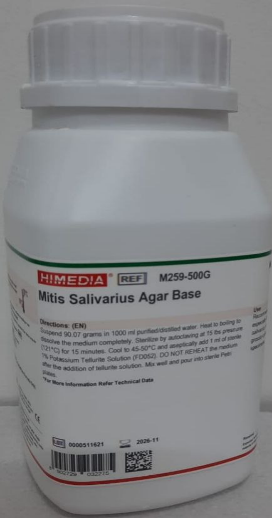

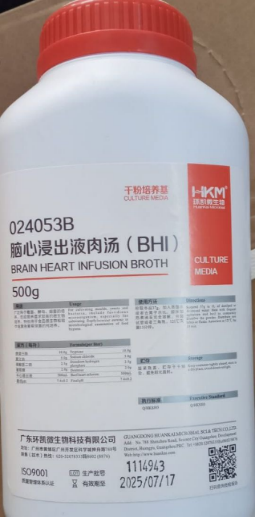

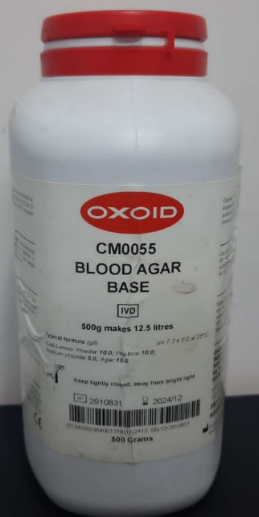


| Table 2.4: materials used in plaque collection and microbiological part | | |
| --- | --- | --- |
| Material | Company | Exp |
| sterile Transport medium swabs | JIANGSU HXRT MD CO., LTD, , China | 2027 |
| Mitis salivarius bacitracin agar | Hemedia india | 2026 |
| Blood agar base | Oxoid/ UK | 2024 |
| Brain Heart Infusion Broth (PH=7) | Hi Media, India | 2025 |

- - 1. **Chemicals in Microbiological Part shown in the (table 2.5) below.**

| Table 2.5:Chemicals in Microbiological Part | | |
| --- | --- | --- |
| Material | Company | Exp |
| Sterile phosphate buffered saline ( PH=7.4) | BDH /England | 2024 |
| Gram stain | Gain Land Chemical Company sandy croft U.K. | 2025 |
| Mannitol | Merck / Germany | 2024 |
| Hydrogen peroxide (3% v/v) (H_2_O_2_) | CDH/ India | 2024 |
| Glycerol | Iraq | 2024 |
| Sucrose | AFCO /India | 2024 |
| Normal Saline | Turktipsan Saglik A.S.(tts company) Turkey | 2023 |
| Vitek 2 GP ID rapid card | Biomerieux, France | 2023 |
| sodium chloride (NaCl) | BDH / England | 2024 |
| Absolute ethanol 99.9% | PRS Panreac, Espana | 2025 |
| Bacitracin antibiotic powder | Appli-Chem, Germany | 2024 |
| McFarland Turbidity Standard No. 0.5 tube . | BBL/USA | 2023 |

**2.2 Instruments**

**2.2.1 Instruments, supplies, and devices for sample cutting and demineralized enamel lesion formation shown in the (table 2.6) and figure (2.5)**

| Table 2.6: Instruments, supplies, and devices | |
| --- | --- |
|  | |
| **Instruments and supplies** | **Origin** |
| Low speed hand-piece | (W&H, Austria) |
| Magnifying lenss10X | (Fukai, China). |
| Ruler 12-inch, 30cm-length | (Sate, China). |
| Pen marker | (Schneider, Germany). |
| Diamond cutting disc (thickness 0.30mm) | (China) |
| Strong 204. dental micromotor handpiece | (sanshein, China). |
| Triple syringe |  |
| Beakers 250 ml capacity | (Renon Lab, China). |
| Quick adjusting vice | Ingco( china) |
| Digital pen type pH meter | (HANNA, Italy) |
| Digital Vernier caliper | (Ningbo, China) |
| Incubator | (Termaks,Norwegian) |
| Rolled laboratory shaker | (DLAB ,china) |
| Polishing machine | (LARYEE TECHNOLOGY, China) |
| Digital Ultra-sonic cleaner | (TreeDental, USA). |
| Mini fridge | China |
| Disposable syringe 5ml | India |
| Disposable Dental tweezers | China |
| Tray Dental towels | China |
| Plastic dental disposable partition tray | China |


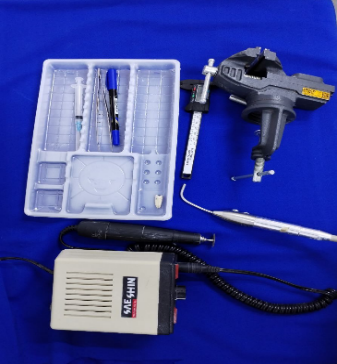

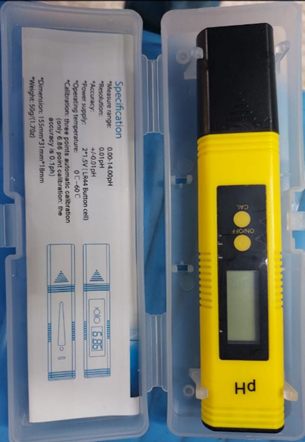

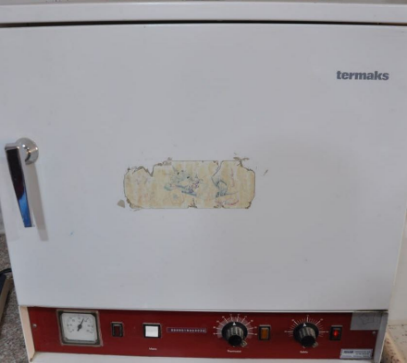

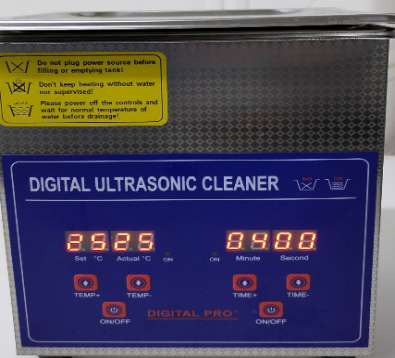

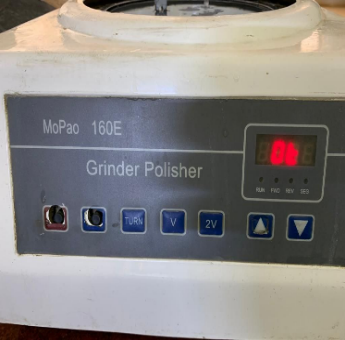

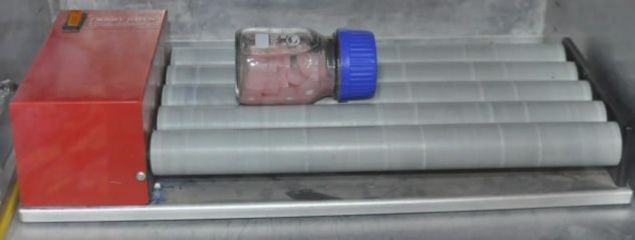


Figure 2-5: Instruments, supplies, and devices for sample cutting and demineralized enamel lesion formation

**2.2.2 Tools used in plaque collection and microbiological part shown in the (table 2.7) below and figure (2.6), (2.7).**

| Table 2.7:Tools used microbiological part | |
| --- | --- |
| **Instruments and supplies** | **Origin** |
| Anaerobic Candle Jar | Rod well - scientific instrument Ltd |
| Bacteriological loop and spreaders | (NerbeplusGmbH&Co.KG/Germany) |
| Cotton roll | (ARTEDENT, Italy) |
| Disposable Mouth mirror | (SinaliDent/China) |
| Disposable plain tube | (Fisher/Malaysia). |
| Disposable Tips | (Dragon lab, Beijing, China) |
| Micropipette | (Mettler Toledo, Germany). |
| PH meter | (High Tech Instruments/China). |
| Plastic petri dishes | (Joy Lab, Shanghai, China) |
| Screw cap | (JinhuaNokeBiotechnology/China) |
| Slides | (Bestscope/China) |
| Spectrophotometer | APEL,Japan |
| Incubator | Shanghai lianhua, china |
| Vortex mixer | Acculab/Korea |
| Vitek 2 compact | BioMerieux, France |
| Deep Freeze | Concord/Lebanon |
| Steam Autoclave | EORAL, China |
| Bunsen burner | Germany |
| Light microscope | KRUSS, German |
| Cooling box | Egypt |
| Saliva ejector | China |
| Point tip tweezer | China |
| dissecting (stereo microscope) | AITAY HAMILTON, Europe |


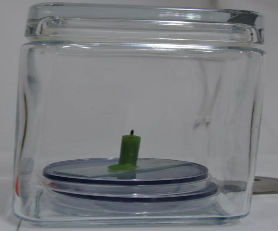

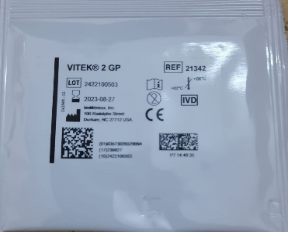

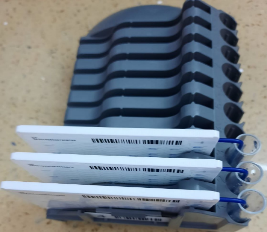

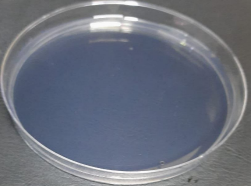

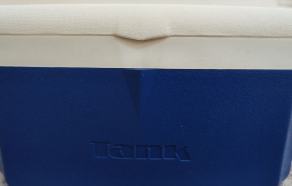

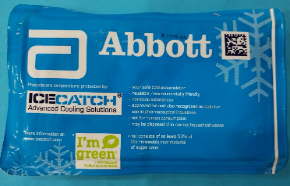

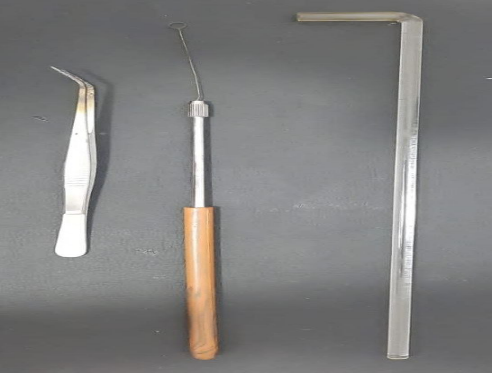

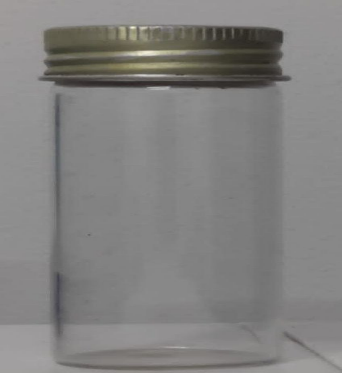


Figure 2-6: Some tools used in plaque collection and microbiological part


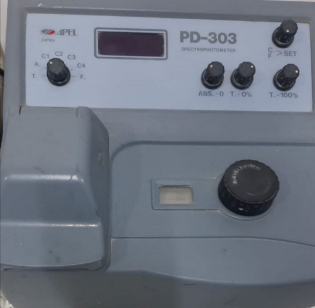

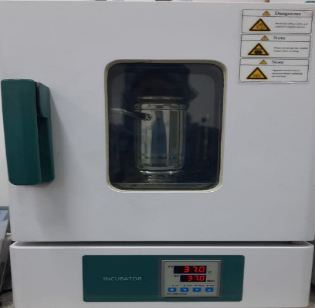

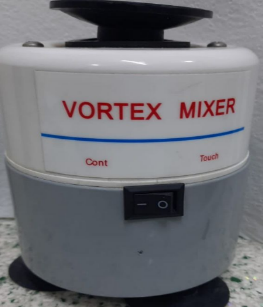

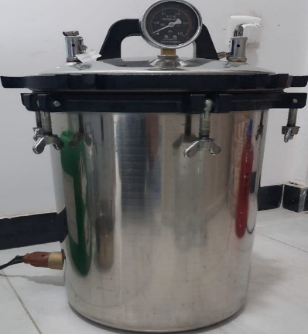

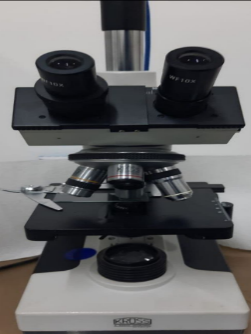

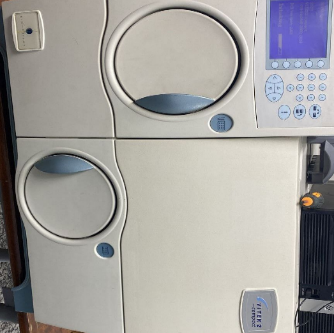


Figure 2-7: Equipment used in plaque collection and microbiological part

**2.3 Equipment to treated and analysis enamel surface**

1. Digital Vickers microhardness tester (Laryee, Model: HVS-1000, Beijing Time High Technology Ltd, China), figure(2-8C).
2. Scanning electron microscope (Thermo Scientific™ Axia™ ChemiSEM, FEI Company, USA) figure(2-8A).
3. Single target plasma coater under vacuum (YKY, China) figure(2-8B).
4. Fractional CO_2_ Laser (CO_2_ Fractional Laser Brochure, JHC1180,

China), figure(2-8D).and device specifications are:

- Laser wavelength: 10600 nm
- Output power: ≤30 W
- Pulse Duration: 0.1-10 ms (adjustable)
- Spots distance: 0.1 – 2.6 mm (adjustable)
- Interval time (time between pulses): 0.1 ms-500 ms (adjustable)
- Mode of scan: order, disorder, parallel (switching)
- Pulse energy: maximum 300 mJ
- Area of Focal Spot: 0.05 mm2
- Output graphic: square, rectangle, triangle, circle, oval, hexagon, linear (scalable)
- Graphic area: ≤ 20 mm × 20 mm
- Optical system: 7 articulated arms

**
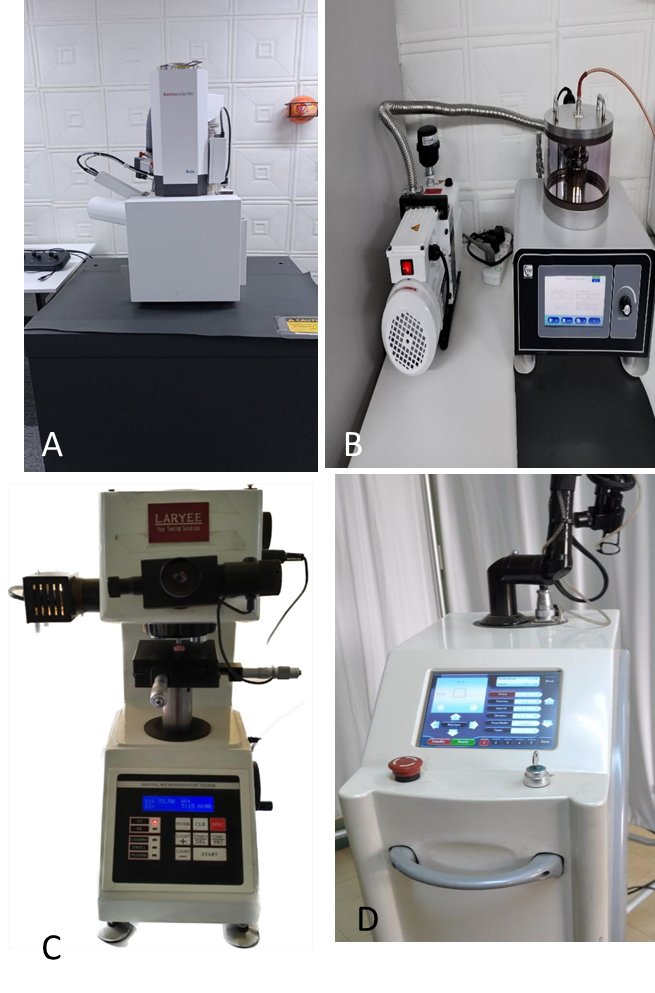
**

Figure 2-8: Equipment(A): SEM, (B): Single target plasma coater, (C): Vickers microhardness, (D): Fractional CO2 Laser.

**2.4 Methods**

This research was conducted in vitro after receiving ethical approval from the College of Dentistry / University of Baghdad's scientific and ethical committees (Approval Number:496522; Appendix). The project spanned from January 2022 to January 2023 and consisted of multiple processes, including the collecting of tooth samples and preparation for the desired hardness measurement, CFU and microscopic examination.

- - 1. **Sample selections**

The samples consisted of forty sound human permanent maxillary first premolar teeth out of 90 teeth which had comparable dimensions by measuring the mesiodistal (M-D) and occlusogingival (O-G) height using a digital Vernier, the height of teeth were (8.40 ± 1.3) mm and the width (8.3± 1.1) mm Figure (2.9). The age of patients was < 20 yrs.

The teeth were extracted for the purpose of orthodontic treatment from Oral Surgery Department at the College of Dentistry / Baghdad University and some private clinics in Karbala. The teeth were washed and cleaned with tap water and polished by non-fluoridated pumice slurry for each sample using a rubber cup in a low-speed handpiece for 10 seconds to remove any remnant debris on the tooth surface. After that, they were washed with de-ionized water and dried with cotton pads, examined by magnifying lens (10X), any tooth had a visible fracture, crack, Decay, or Restorations was discarded The teeth were then placed in 0.1% thymol solution (as anti-microbial solution for inhibition of bacterial growth) and stored for one day. Then placed in a de-ionized water in a cold cabinet (+4°C) until use (Sorkhdini et al., 2020, Wang et al., 2022, Al-Shareefi et al., 2022).


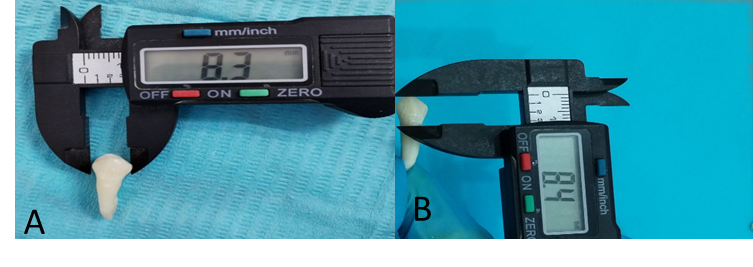


Figure 2-9: Measuring the dimensions of tooth (A): M-D width of buccal surface, (B): O-G length of the buccal surface,

**2.4.2 Enamel sample preparation**

On all of the specimens, the roots separated from the crowns with a low-speed diamond disc under constant cooling water. Each crown was sectioned mesiodistally into two-halves obtaining the buccal half only, then each surface was further sectioned in to 4 slabs (4.0 × 4.0 × 2.0 mm) figure. the specimens (n=160) were divided in two groups(n=80) figure, slabs used for colony forming unit measurement, a separate medium in form of petroleum jelly was used to coat inner surface of each slabs applied to prevent it adhesion to acrylic material and to facilitate specimen removal from block figure. While for Vickers microhardness tester without separated medium.

A specially designed cubic silicone mold with internal dimensions 1.5 cm x 1.5 cm x 1.5 cm was used for the construction of acrylic blocks. The cold cure acrylic resin mixed according to the manufacturer , powder and liquid 2/1 by volume until the dough stage was reached then the acrylic was poured in the mold and the enamel side facing upward figure, slab was placed according to the level determined before then left in place until the acrylic set for 30 mins (Navimipour et al., 2012).

In order to guarantee a flat surface for accurate measurements, the surfaces were polished using a polishing machine figure(Laryee Technology CO.LTD, China) with silicon waterproof abrasive papers sequentially (P1200 for 10 s, P2500 for 10 s and P4000 for 4 min in association with water to achieve a smooth and finished enamel surface (Alturki et al., 2020). Ultrasonication was conducted for 1min after P1200 and P2500 and 4 min after P4000 to remove the smear layer. The samples were stored in deionized distilled water to prevent dehydration.

Figure 2-10:(A)Stabilizing the tooth on Quick adjusting vice
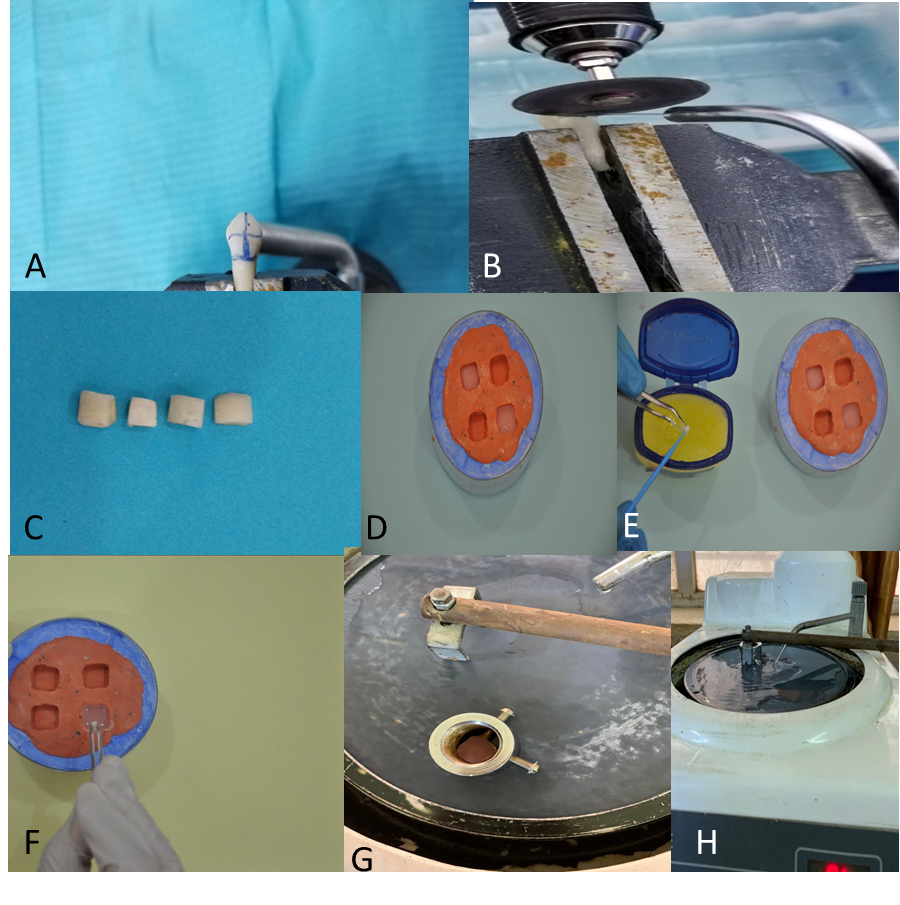
 ,(B)tooth sectioning using diamond disc,(C) enamel slabs after sectioning,(D) acrylic poured in the mold,(E) applied separate medium to inner surface of slab,(F)slabs mounted in acrylic,(G) place enamel block on holder for standardize polishing procedure,(H) enamel slab polished by polishing machine.

Figure 2.11: enamel slab in acrylic block.


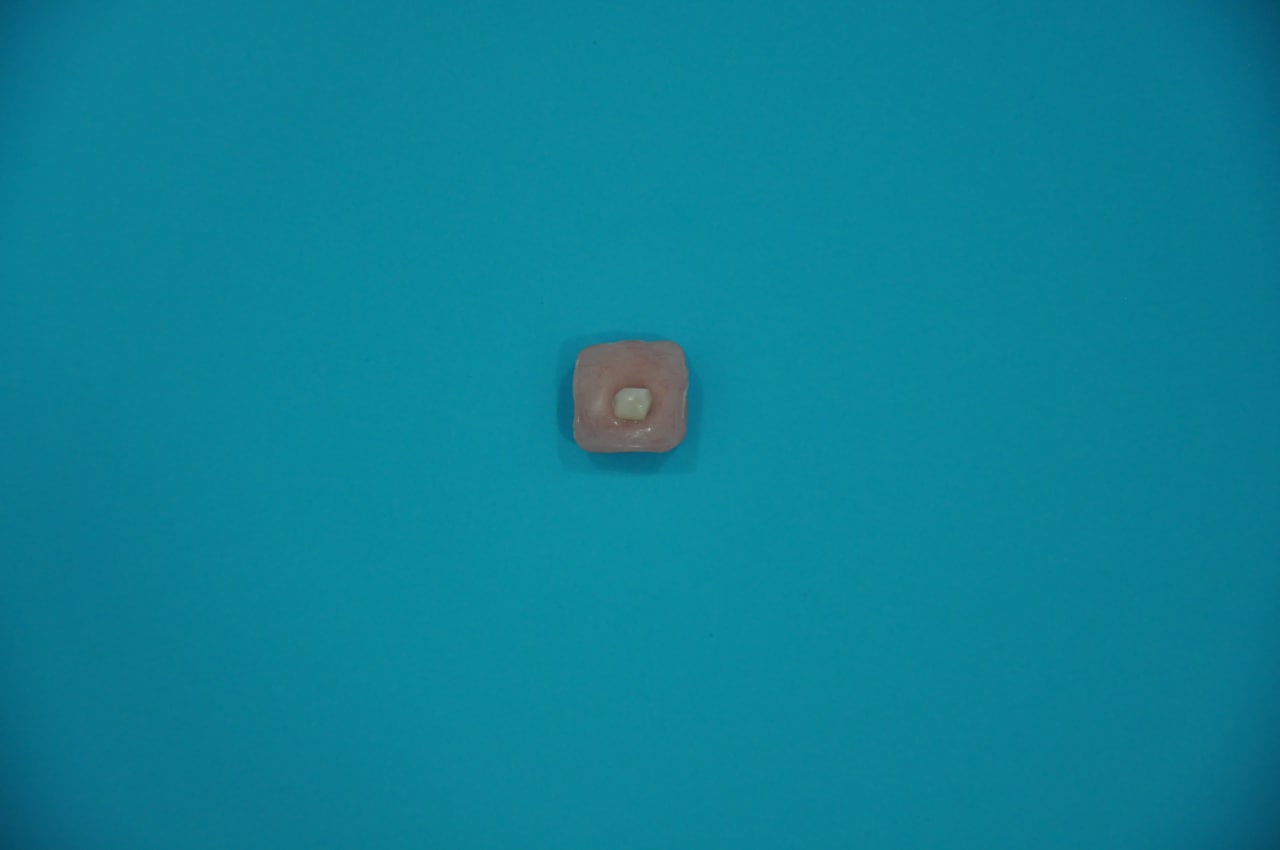

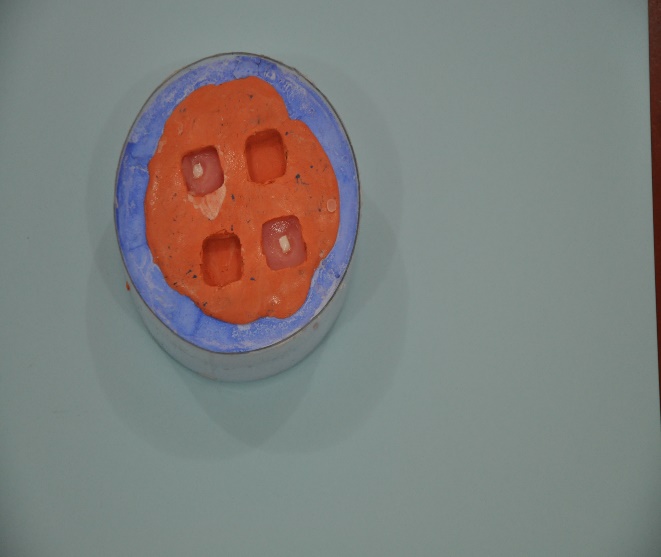


**2.4.3 Sample grouping and study design**

160 Enamel slabs were divided as follow, 80 enamel slabs were subjected to CFU examination, the rest 80 enamel slabs have been subjected to VMH Test, Teeth were randomly allocated in to two main groups; sound and demineralised enamel (n=80 per group) figure (2.12). and so there were four different sub-groups (n=20) in each main group figure (2.13):

Group 1: Sound enamel as a control (n=20)

Group 2: sound enamel samples were irradiated with CO2 laser beams (n=20)

Group 3: Sound enamel samples were treated with MI Paste Plus (n=20).

Group 4: Sound enamel samples were first treated by MI Paste Plus with then irradiated with CO2 laser beams(n=20).

Group 5: Demineralized enamel (n=20)

Group 6: Demineralized enamel samples were irradiated with CO2 laser beams (n=20).

Group 7: Demineralized enamel samples were treated with MI Paste Plus (n=20).

Group 8: Demineralized enamel samples were first treated by MI Paste Plus and then irradiated with CO2 laser beams, (n=20).


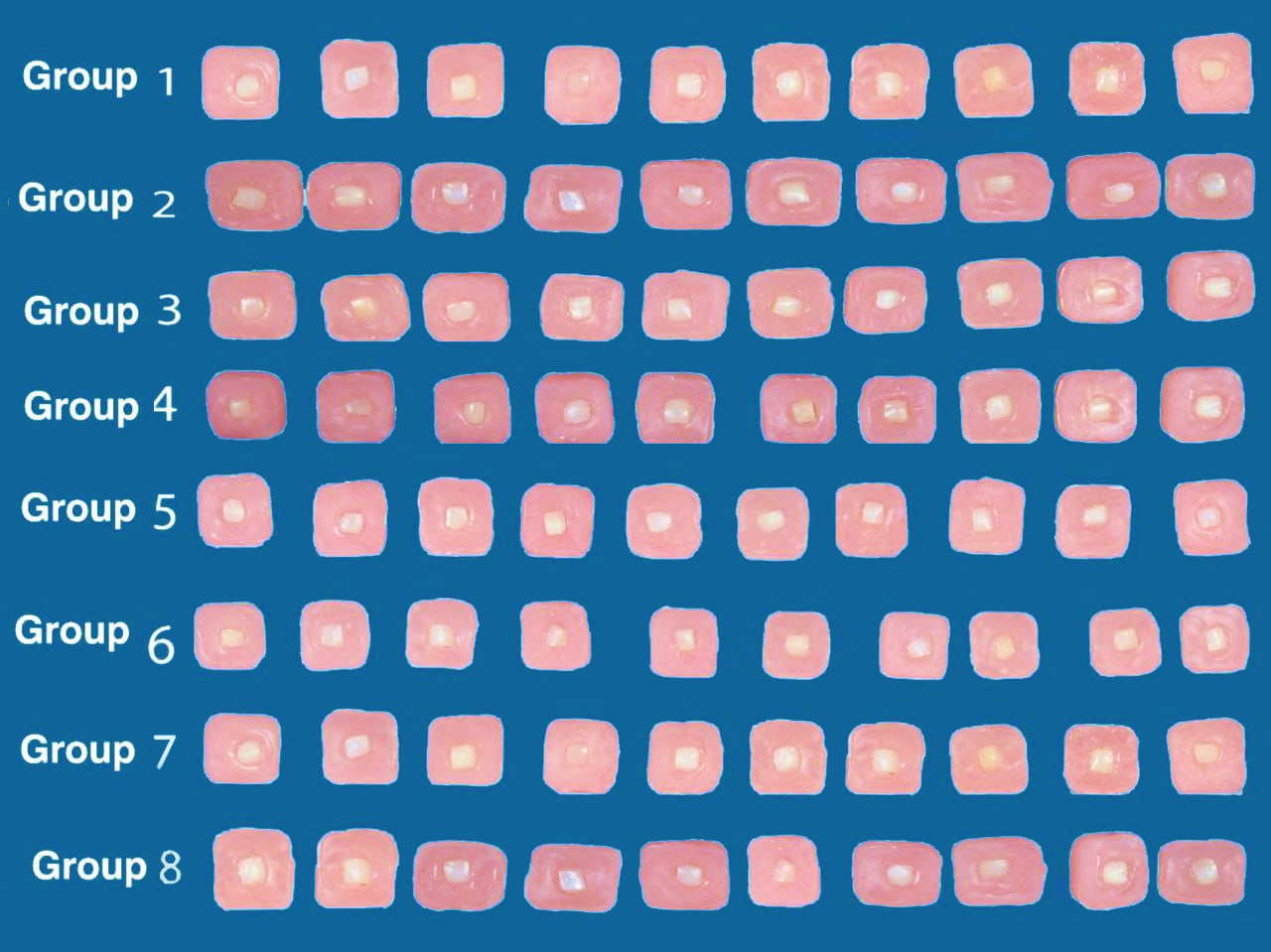
Figure 2.12: main groups


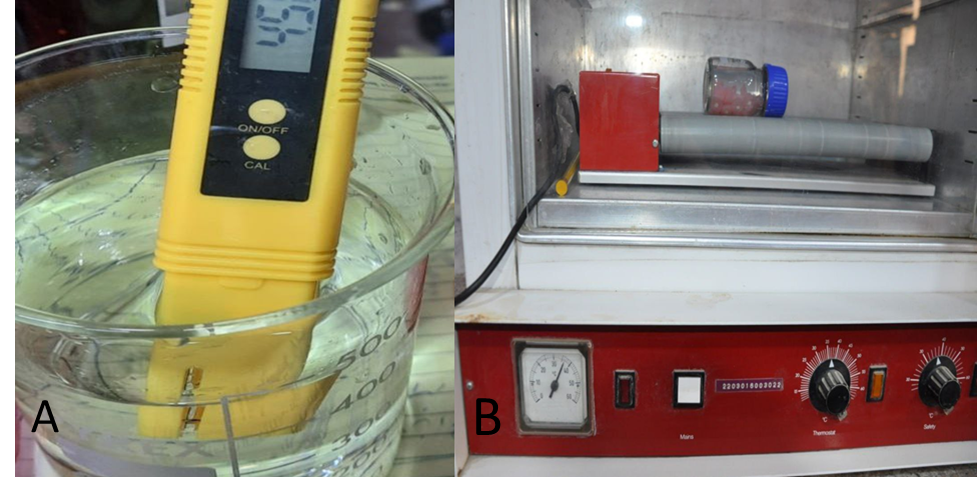


**2.4.4Enamel Carious Lesion Formation:**

Eighty enamel slabs in groups 5,6,7 and 8, were immersed in demineralization solution (50 mM acetic acid, 2.2 mM Ca (NO3)2, 2.2 mM KH2PO4, 5.0 mM NaN3, and 0.5 ppm NaF, pH 4.5). The Blocks were immersed in demineralization solution (4 mL per block) at 37°C for 3 d in a shaker without solution refreshing (37°C, 50 rpm/min), figure( 2.14) resulting in subsurface demineralization-like initial enamel caries(Fan et al., 2021).

Figure 2.14: A) demineralization solution with pH meter adjusted to 4.5), B) Blocks immersed in demineralization solution at 37°C


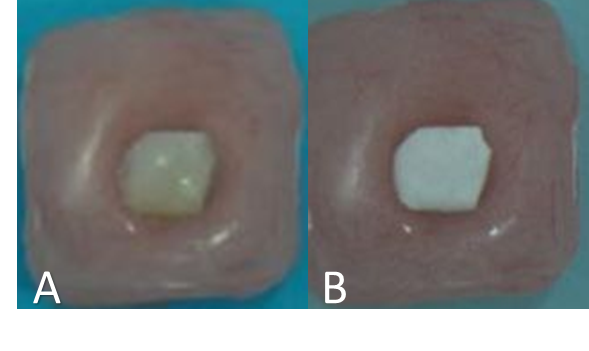


Figure 2.15: (A) sound enamel slabs, (B) enamel slabs after demineralization procedure.

**2.4.5The application of MI paste PLUS**

The MI Paste plus (GC MI Paste Plus^®^ with RECALDENT™ (CPP-ACP) was applied on sound and demineralized enamel surfaces (group 3, 4, 7&8) (n=20 per group) following the manufactures’ instructions. In which a Micro Applicator Brush was used to dispense the paste over enamel surfaces and left undisturbed for three minutes. The paste was spread over the surface for one minute in a circular motion, then washed away after five minutes with deionized water for 30 sec. figure (2.16), and then rinsed with deionized water for 30sec(Llena et al., 2019).


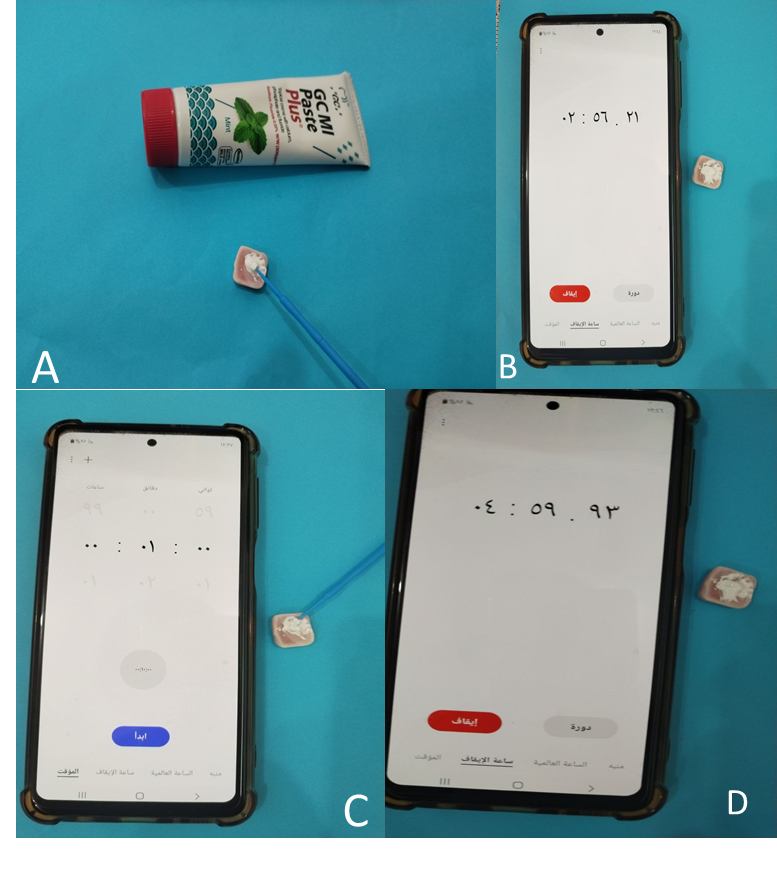
This procedure was repeated once application daily for two weeks.(Heshmat et al., 2016, Mehta et al., 2013). , and specimens were kept in artificial saliva at 37 °C during application period figure (2.17)(Zhang et al., 2011).

Figure 2.16: A) application MI paste PLUS, B) left undisturbed for three minutes, C) spread over the surface for one minute in a circular motion, D) left five minutes


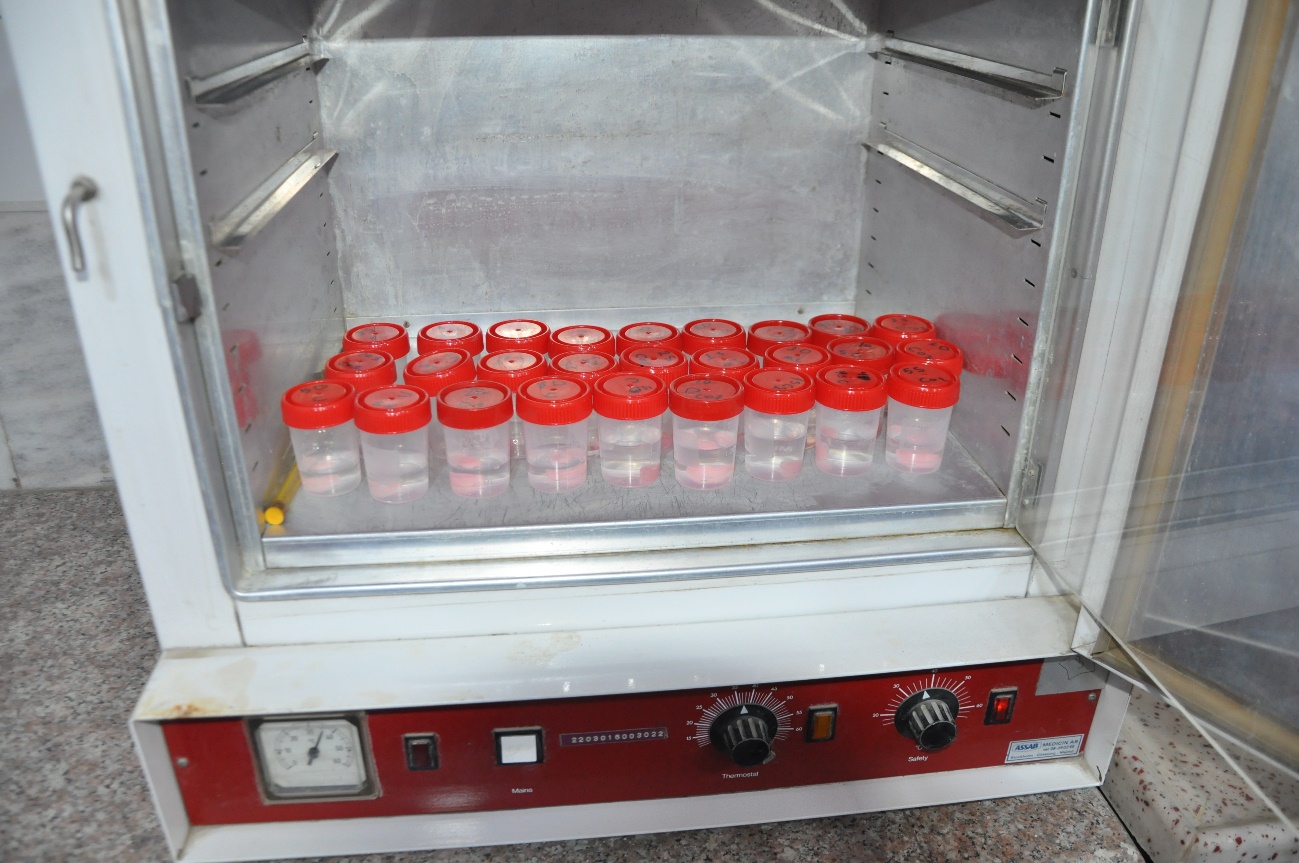
Figure 2.17: samples saved in the artificial saliva at 37 °C

**2.4.6 The application of carbon dioxide laser CO_2_**

A commercially pulsed CO_2_ laser (CO_2_ Fractional Laser, JHC1180, China) was applied to sound and demineralised enamel surfaces (subgroup 2, 4, 6&8) (n=20 per group) using the following parameters: 2 W power, 10 ms pulse duration, 50 Hz pulse frequency, 0.2 mm focal spot, and 11.5 J/cm^2^ energy figure (2.18A). Surface scanning was done for 5 secs from an X-Y positioning platform in noncontact mode keeping a 10 mm distance between the handpiece tip and the treated surfaces figure (2.18B) (Steiner-Oliveira et al., 2006). In the last subgroups (4&8) (n=40), of both sound and demineralised enamel, the surfaces were treated by MI paste for two weeks then exposed to CO_2_ laser following the same procedures of both. Then, all specimens were kept in artificial saliva at 37°C until the next step(Kasraei et al., 2021).


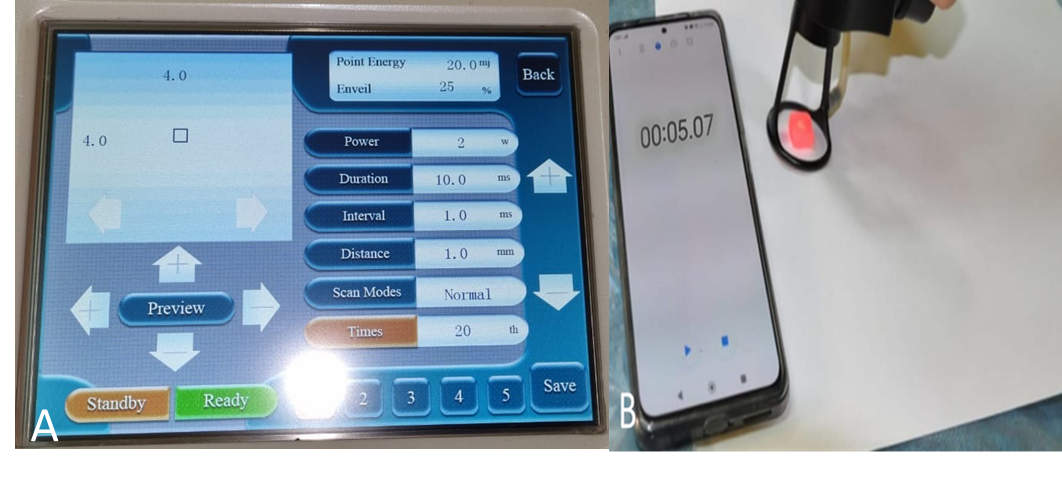
Figure 2.18: A) laser parameters B) position of sample during lasing.

**2.4.7 Plaque biofilm collection:**

Plaque samples were collected from five healthy individuals attending the College of Dentistry-University of Baghdad, with no oral disease with an age range of 25-54 years, after applying the patients’ consent form and approval, the samples were collected from December 2021 to January 2022. A microbiological collection swab containing transport media was used for collecting samples after drying the surface to prevent contamination with saliva. Then, each swab (n=5) was immersed individually in 1 mL of sterile phosphate buffered saline immediately, then kept inside an icebox until being transferred to the laboratory (Hsu et al., 2010).

**2.4.8 Preparation of the culture media and solutions:**

**2.4.8.1 Phosphate buffer saline (PBS):**

A phosphate buffer saline of pH 7.0-7.2 was prepared by dissolving a tablet of phosphate buffer (LDH) in 100 ml of deionized water and spread in ten test tubes (9.9ml) and sealed with a piece of cotton and tin foil and then sterilized in the autoclave. Then it was left to cool down and finally stored in the fridge until the time of its use(Al-Huwaizi and Al-Alousi, 2013).

**2.4.8.2 Bacitracin solution:**

Preparation of bacitracin was achieved by dissolving 0.364g of bacitracin antibiotic in 100 ml. of sterilized distilled water. A magnetic stirrer was used to ensure the complete dissolution of the antibiotic then the Bacitracin (antibiotic) solution was sterilized by the filtration using Millipore filter with size (about 0.22 μm)(Al-Mizraqchi, 1998).

**2.4.8.3 Preparation of Cystine Trypticase-Mannitol Agar (CT-Mannitol Agar):**

Preparation was done according to the instructions of the manufacturer by suspending 28.5gm powder in 1000 ml of D.W, mixed by using magnetic stirrer to ensure dissolution of the whole quantity of the powder. After the preparation of Cystine Trypticase Agar (CTA) as, 1% mannitol was added to the CTA media and heated to ensure dissolution of the whole quantity of the powder in the CTA media and then was distributed into screw capped bottles (10 ml in each bottle), autoclaved and stored in the refrigerator until used.

**2.4.8.4 Preparation of Mitis Salivarius Bacitracin Agar (MSBA) medium**

Mitis salivarius Bacitracin agar is a type of selective media used to isolate Mutans streptococci cells, other bacteria than MS were inhibited by this medium. According to manufacturer, 90 gram of powder was suspended in 1000 ml of distilled water for media preparation. The magnetic stirrer was used to guarantee that the powder was completely dissolved. Then Sucrose in a concentration of 150g/l was added and after autoclaving the medium and cooling it to 45°C, 1ml of bacitracin in a concentration of 200 I unit/l (0.36g of powder in 100 ml) was added to each liter of agar. The media was then poured into sterilized petri dishes figure (2.19) and allowed to cool and set. Then stored in the refrigerator until
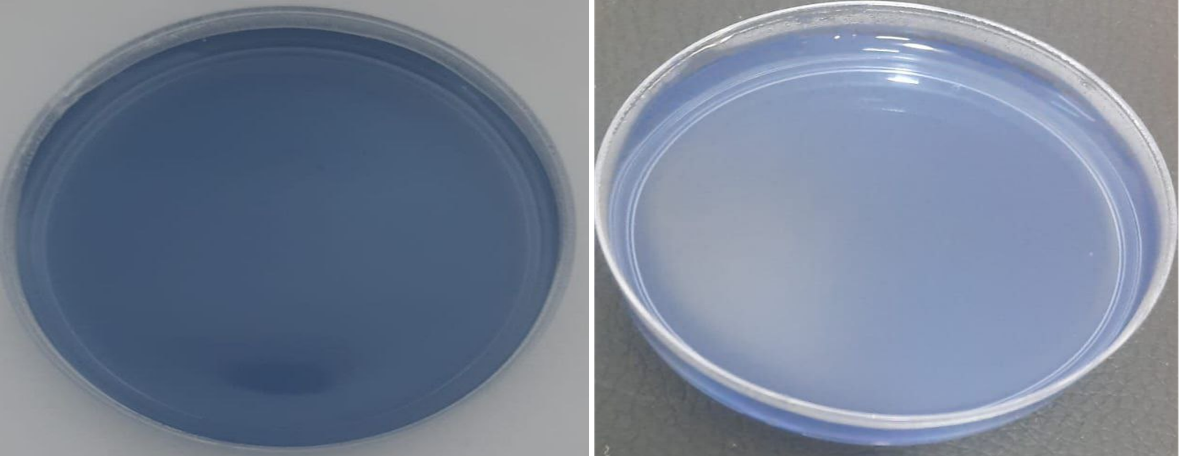
used.(Hamad and Atiyea, 2021).

Figure 2.19: Mitis salivarius bacitracin agar

**2.4.9 Sterilization:**

Hot air oven at 180 °C for 1 hour, was used for the sterilization of glassware, steel equipment’s and metal instruments while autoclave at 121°C at 15 pounds per square inch was used for the sterilization of all the culture media, phosphate buffer saline for 15 minutes. The sterilization of bacitracin solution was achieved by Millipore filter (0.20µm)(Fathallh and Mahmood, 2021).

**2.4.10 Isolation of *S. mutans***

The plaque collected samples were immersed in 1 ml of phosphate buffer saline then subjected to vortex for a minute to disperse the plaque and obtain a homogeneous suspension (Mathew et al., 2020). Serial dilution of each sample was prepared at concentrations of 10^-1^- 10^-5^, which was cultured 100 µL from each dilution in selective media for *S. mutans* (Mitis salivarius bacitracin agar, MSBA) and incubated anaerobically 48 h at 37°, followed by 24 h in aerobic condition (Al-Mahmood et al., 2020). At the end of the incubation period, streptococcal colonies appearing on these plates were selected and identified by testing figure (2.20).


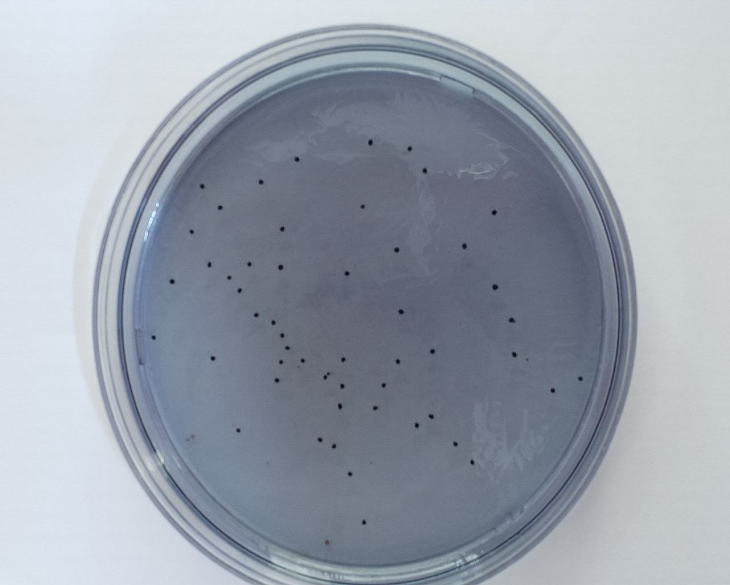

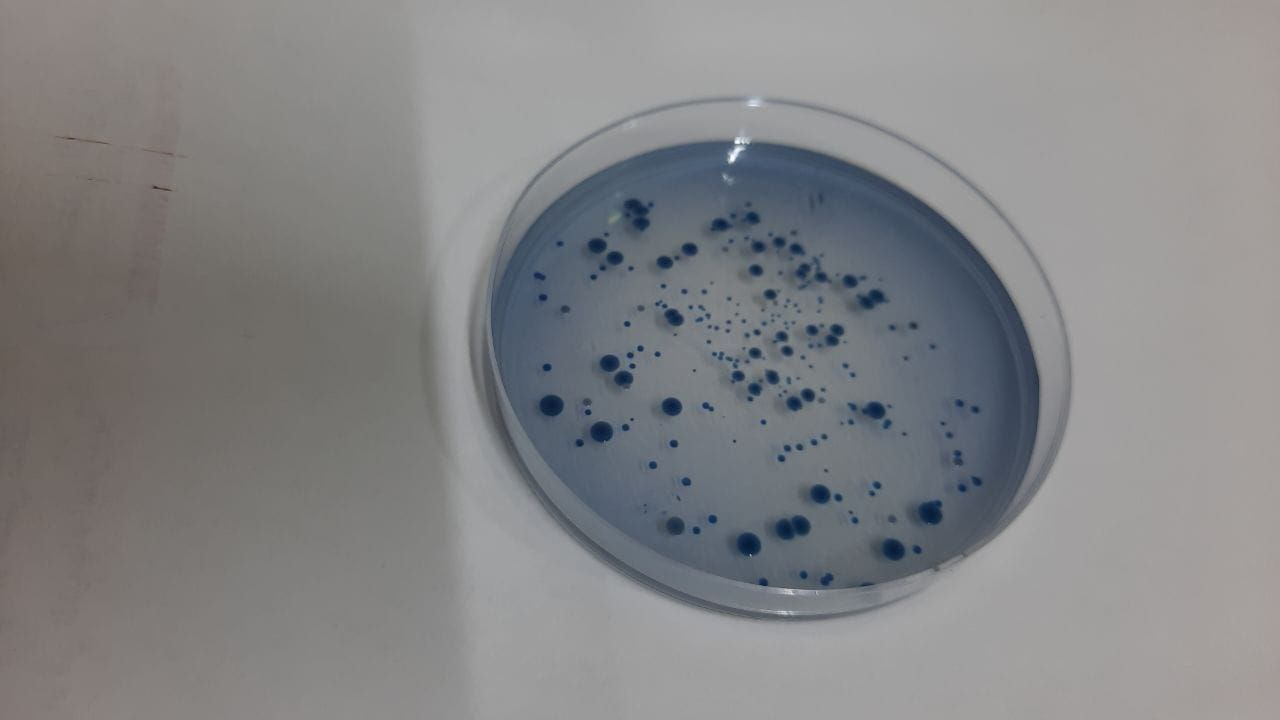


Figure 2.20: A) *mutans streptococci* B) Colonies of *Streptococcus mutans* on MSBA

**2.4.11 Identification of *S. mutans***

**2.4.11.1 Colony morphology:**

*Streptococcus
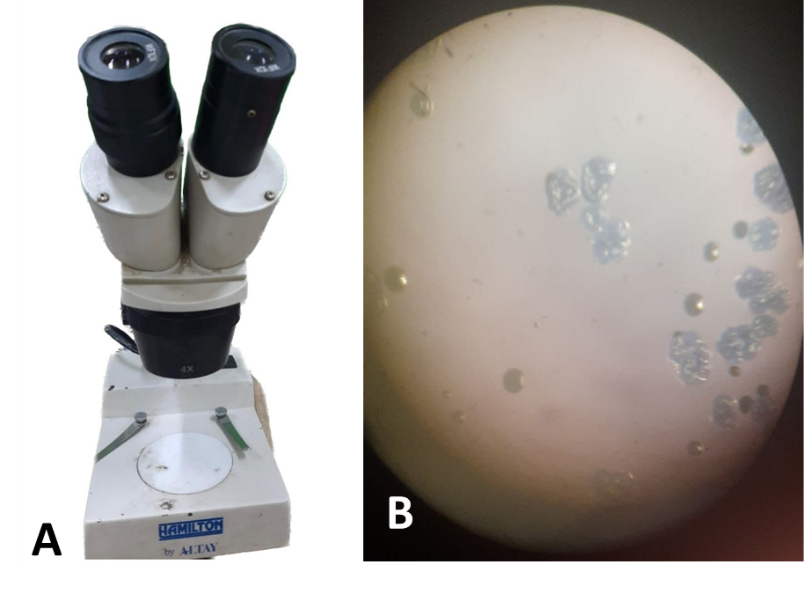
Mutans* colonies were inspected under dissecting microscope at a magnification of 20X description of morphological features on MSBA plates which appeared under as; round or spherical in shape, dark blue color, pin point size, convex with rough surface, and arranged in chains figure(2.21) (Kamble et al., 2022).

Figure 2.21: A) dissecting microscope, B) Colony of *Streptococcus mutans* on MSBA (20Xmagnification) under dissecting microscope

**2.4.11.2Bacterial cell morphology (Microscopical analysis)**

A colony was picked up from MSBA plates under the sterilized conditions and subjected to Gram stain, light microscope figure (2.22).(Saleh et al., 2023). then Staining of the slide completed according to Gram's method(Koneman et al., 1992) as following:

1. The slide flooded with crystal violet for one minute, and then rinsed with water.

2. Gram's iodine was added to the slide allowing it to set for one minute then rinsed with water.

3. Decolorized with aceton- alcohol by holding the slide at an angle and rinsed until solution began to fade from dark blue to colorless, then rinsed immediately with water.

4. Counter stained with safranin for one minute then rinsed and dried. The slide was examined under light microscope.


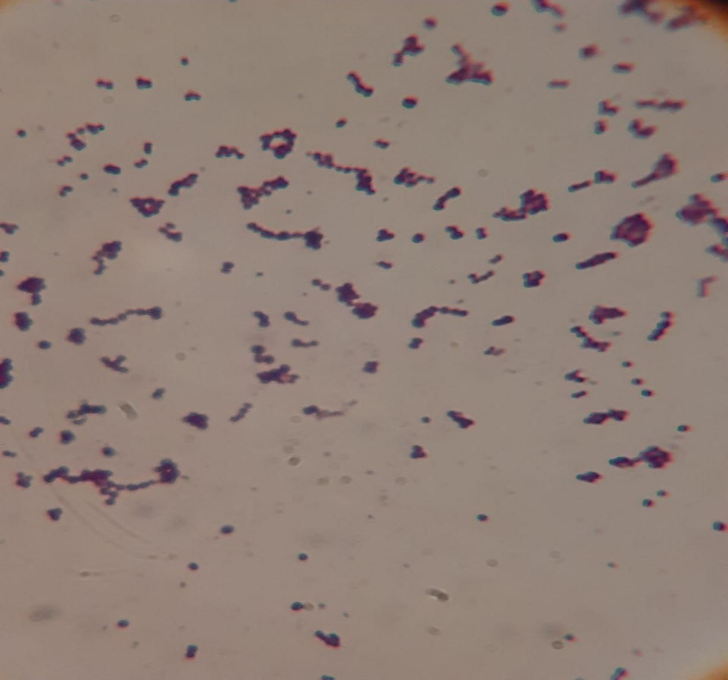


Figure 2.22: Gram`s staining of *Streptococcus mutans* under a light microscope (1000×).

**2.4.11.3Biochemical tests:**

**2.4.11.3.1Catalase test**

Catalase test was carried out by placing a small amount of the growth from the culture onto a clean microscopic slide and adding a one drop of 3% H2O2. A positive result is the rapid evolution of O2 as evidenced by bubbling, while a negative result is no bubbles. *S. mutans* are known to be catalase negative; therefore, colonies showing negative result(Ahmed et al., 2019).

**2.4.11.3.2 Mannitol fermentation test**

All colonies of *Streptococcus mutans* fermented sugar. As a result of the carbohydrates fermentation and acid formation, the color of the indicator changed from red to yellow figure (2.23), indicating a positive result.(Hamad and Atiyea, 2021). Cystine Trypticase-Mannitol Agar had been used to test the ability of M.S. to ferment the mannitol. Each bottle was inoculated with 0.1ml of pure M.S. isolates and incubated aerobically for 48hrs at 37◦C. Changing in color from red to yellow indicated a positive reaction in comparison to the positive control (broth and bacteria without mannitol) and negative control (broth and mannitol without bacteria) due to acid production from the carbohydrate fermentation reaction which caused a pH reduction


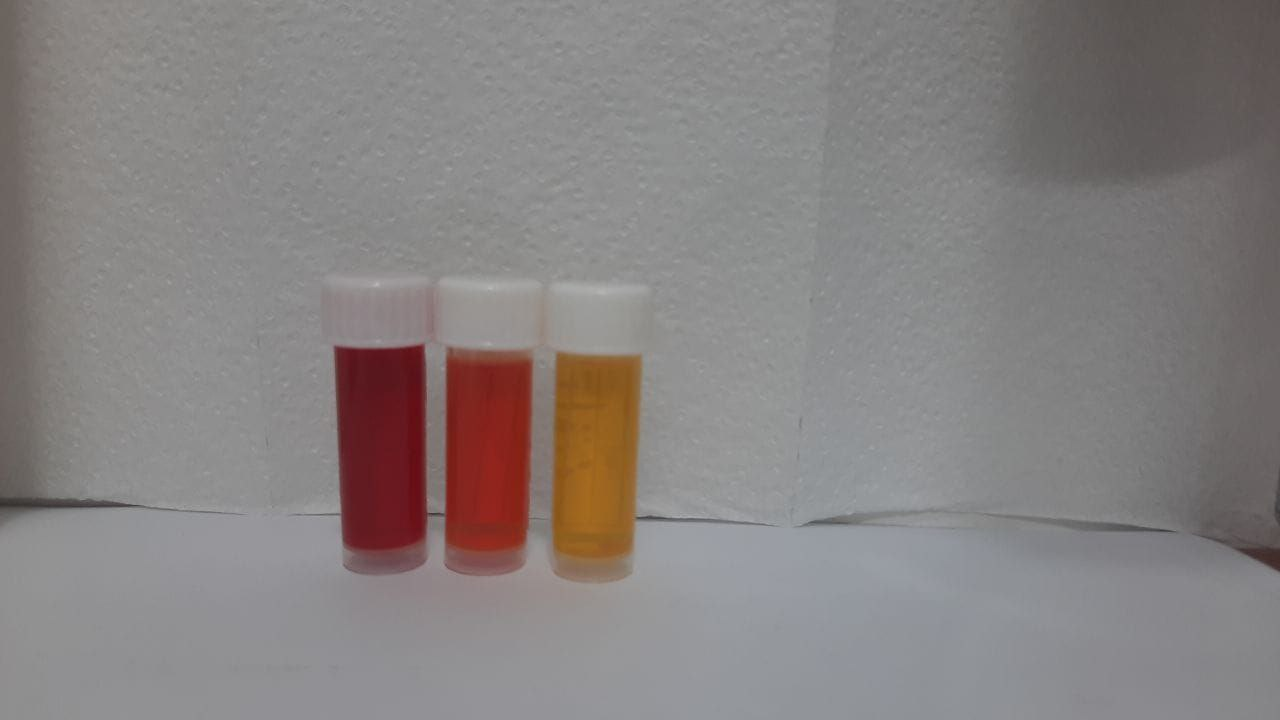


Figure 2.23: Mannitol fermentation test of Streptococcus mutans. A: Positive control tube (agar and bacteria without mannitol). B: Negative control tube (agar and mannitol without bacteria). C: Study tube (agar and mannitol inoculated with Streptococcus mutans)

For more confirmation of bacterial diagnosis, the Vitek 2 compact system was used (Appendix II).

**2.4.12 Maintenance of bacterial isolate.**

Selective colony from each isolate was transferred to 1 ml sterile BHI-B and incubated for 24 hrs at 37◦C aerobically. then from this broth an appropriate amount was taken and 20% of sterilized glycerol was added to the inoculated broth and stored at −80°C, until used .the tubes were labeled (the type of inoculum and the date of inoculation(COTA and ALVIM, 2018)

**2.4.13 Activation of Streptococcus Mutans inoculums:**

activation before each experiment was prepared by addition of 0.1 ml of pure MS isolates to 10 ml of sterile BHI-B (pH 7.0), and followed by incubation aerobically for 18hrs (Al-Huwaizi and Al-Alousi, 2013).

**2.4.14 Determination of viable count:**

All freshly grown bacterial suspensions in 5 mL of their specific broth media were suspended to 1.5× 10^8^ CFU (colony forming unit)/mL according to the turbidity of 0.5 McFarland test standard, and the concentrations of the bacterial suspensions were adjusted spectrophotometrically at an optical density of 600 nm (OD_600_) to match the turbidity of all of the suspensions with 0.5 McFarland test standard(Akca et al., 2016).

**2.4.15 The application of *Streptococcus mutans* at enamel surfaces.**

All enamel blocks (sound and demineralised, n=80) were sterilized in an autoclave at 121°C for 15 min(Afrasiabi et al., 2020). Then each block was placed individually in a sterilized screw-capped bottle containing 2-mL of the adjusted bacterial suspension (1.5 × 10^8^ CFU/mL) and 1 mL of BHI contains 1% sucrose, and incubated aerobically at 37 °C for 24 h figure (2.24) to simulate the cariogenic challenge on enamel surfaces by the *S. mutans* biofilm(Yan et al., 2018, Kim et al., 2022) After that, each block was removed from the tube, immerse twice by saline solution in order to remove loosely bound bacteria. Then each enamel slab was removed from the acrylic block with sterile tweezer carefully and inserted in a tube contains 1 ml of phosphate buffer saline (pH 7.2), and stirred by Vortex for one minute to detach the bacteria from the surface. This suspension was further diluted to ten-fold (Güorgan et al., 1997) , and 100 μL of each suspension was transferred to mitis salivarius bacitracin agar and incubated in 5% CO2 at 37°C for 48 h (Espinosa-Cristóbal et al., 2013).


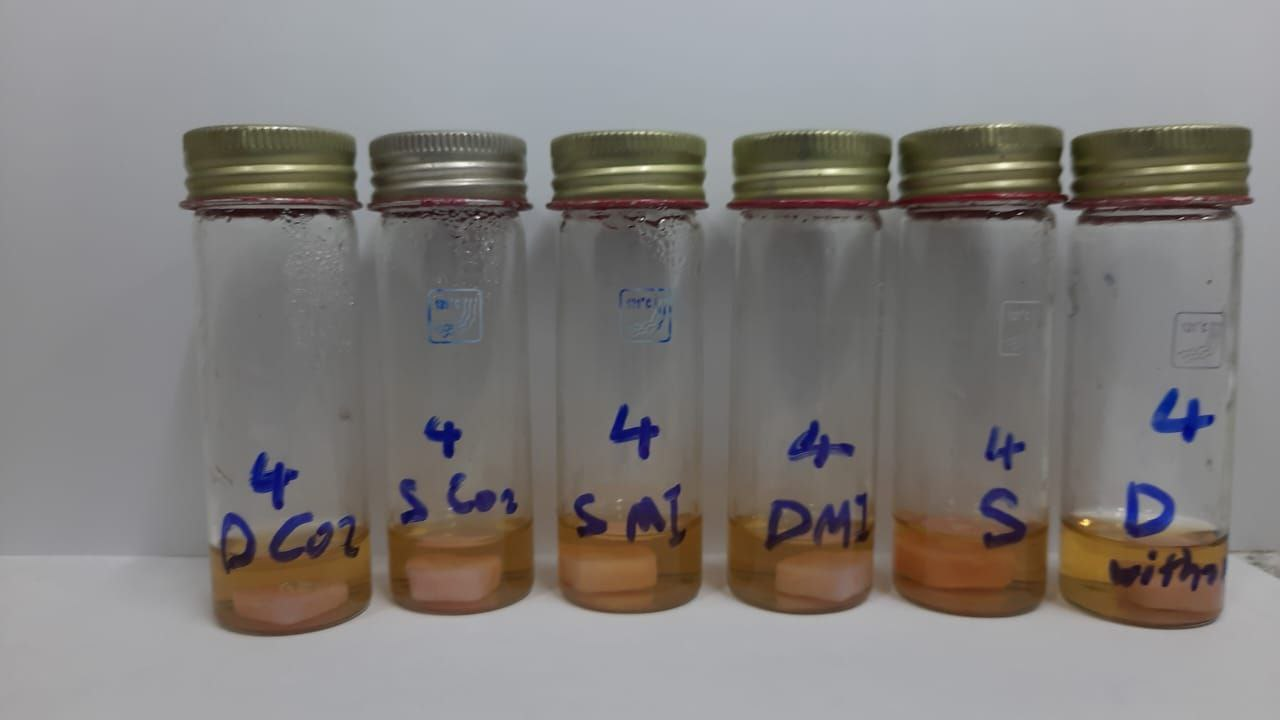


Figure 2.24: blocks in a sterilized screw-capped bottle containing bacterial suspension

**2.4.15 Calculation of *S. mutans* (CFU/mL)**

After incubation of bacterial culture for 48hours at 37˚C, total viable count was carried out for the plates. the plates that contain 30-300 bacterial colonies(Hassan et al., 2010) Usually the 2^nd^ dilution was depended according to a pilot study as the plate was contain 73 bacterial colonies duplicate plates (with same amount plated) have been made from each dilution, the average number of colony counts was divided.

The following equation was used to identify the number of bacterial cells into each 1 ml of the samples. **Number of bacteria (CFU/ml) =No. of colonies in MSB plate ×10^n^ ×10."**When (10^n^) is the dilution factor. (Reynolds, 2005a).

Counting and calculating method:

The plate counting method was used in the current study; the plate count method relies on(Harley and Prescott, 2002).

1. the ability of bacteria to form a colony on a nutrient medium,

2. the colony can be seen by the naked eye

3. colonies on a plate can be counted(Hailan and Al-Khatieeb, 2019)

**2.4.16 Surface Microhardness:**


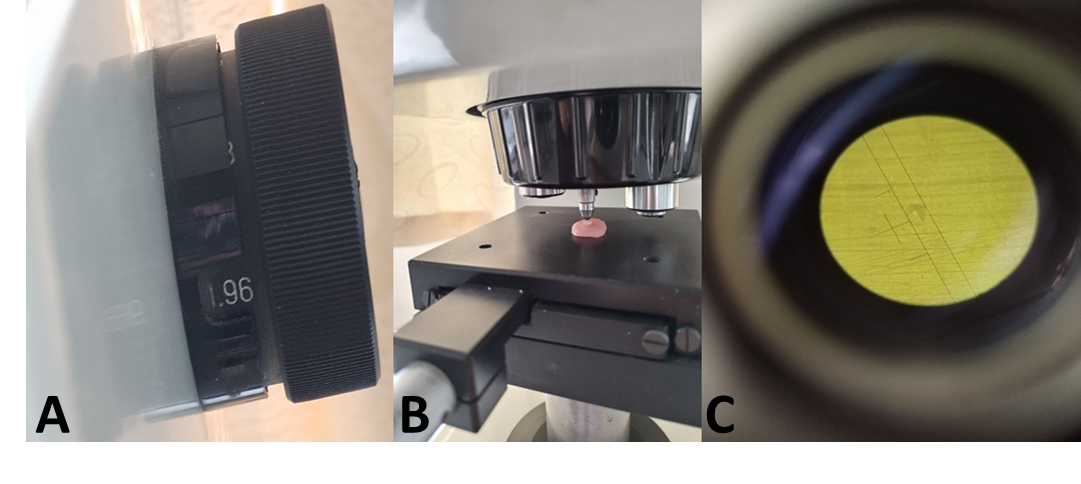
The Hardness profile of sound and demineralized enamel surfaces (n=80) was examined by using Vickers microhardness tester using a diamond square-based pyramid diamond-shaped indenter with a 200 gf (1.96 N) load for 15 sec figure(2.25A,B) (Shubbar et al., 2023)Three indentations were done at middle of each surface with 500 µm distance apart. along a line parallel to the specimen's external surface for 15 seconds, resulting in a diagonal on the surface figure (2.25C) The Vickers hardness number was recorded automatically using the manufacturer’s software, in which the value was an average of three readings.

Figure 2.25 –A, B) Load applied on the enamel surface, C) The diagonal formed above the tooth surface.

**2.4.17 Morphological observation:**

Two specimens of each subgroup were observed under Scanning Electron Microscopy combined with energy dispersive X-ray spectroscopy (EDX). The biofilms on enamel surfaces were washed three times with phosphate-buffered solution (pH 7), Samples were subsequently mounted directly on aluminium SEM stubs without prior fixation figure(2.26A), (Kokkinosa et al., 1998). After that, all specimens were sputter coated with gold figure (2.26B) and vacuum dried to be examined under SEM-EDX figure(2.26C), at 10 kV and two magnification powers (2500x and 5000x), and the working distance at 50 µm and 30 µm, respectively. to assess surface morphology and the bacterial aggregation on enamel surfaces, while the mineral profile was examined by EDX.


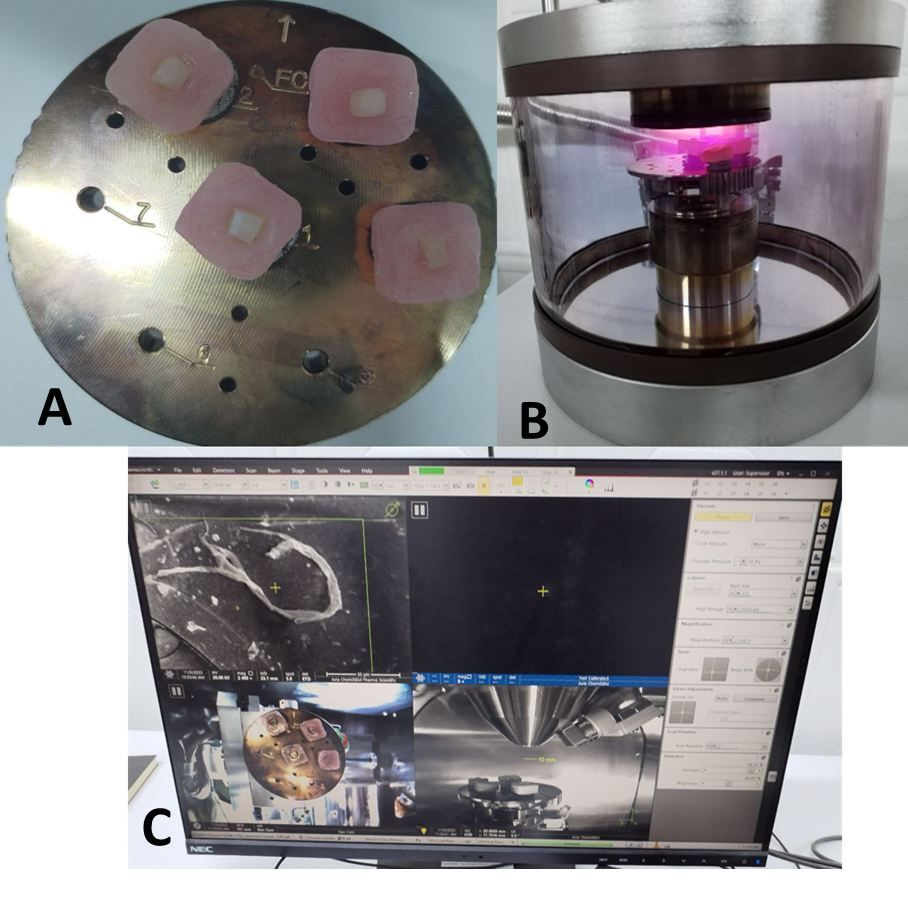


Figure 2.26–A), mounted blocks on aluminium SEM stubs B) sputter coated with gold, C). vacuum dried and examined under SEM-EDX
