# Supplementary material for: The preventive/therapeutic effect of CO2 laser and MI Paste Plus® on intact and demineralized enamel against Streptococcus mutans (In Vitro Study)
Source: Heliyon. 2023 Sep 23;9(10):e20310. doi: 10.1016/j.heliyon.2023.e20310 (PMC10543189; doi:10.1016/j.heliyon.2023.e20310)
Supplement: Multimedia component 3 [file mmc3.docx]

DATASET ACTIVATE DataSet1.

DATASET CLOSE DataSet2.

DESCRIPTIVES VARIABLES=Soundcontrol SCO2 SMI SCO2Mi Demineralised DCO2 DMI DCO2MI

/STATISTICS=MEAN STDDEV MIN MAX SEMEAN.

**Descriptive**

| **Descriptive Statistics** | | | | | | |
| --- | --- | --- | --- | --- | --- | --- |
|  | N | Minimum | Maximum | Mean | | Std. Deviation |
|  | Statistic | Statistic | Statistic | Statistic | Std. Error | Statistic |
| Sound control | 10 | 18000 | 23000 | 20200.00 | 442.217 | 1398.412 |
| SCO2 | 10 | 15000 | 20000 | 17200.00 | 512.076 | 1619.328 |
| SMI | 10 | 13000 | 17000 | 14900.00 | 458.258 | 1449.138 |
| SCO2Mi | 10 | 400 | 760 | 587.50 | 40.244 | 127.263 |
| Demineralised | 10 | 23000 | 31000 | 25700.00 | 856.997 | 2710.064 |
| DCO2 | 10 | 13000 | 17000 | 15700.00 | 422.953 | 1337.494 |
| DMI | 10 | 10000 | 16000 | 12300.00 | 558.768 | 1766.981 |
| DCO2MI | 10 | 1000 | 4000 | 2600.00 | 305.505 | 966.092 |
| Valid N (listwise) | 10 |  |  |  |  |  |

EXAMINE VARIABLES=Soundcontrol SCO2 SMI SCO2Mi Demineralised DCO2 DMI DCO2MI

/PLOT BOXPLOT STEMLEAF HISTOGRAM NPPLOT

/COMPARE GROUPS

/STATISTICS DESCRIPTIVES

/CINTERVAL 95

/MISSING LISTWISE

/NOTOTAL.

**Explore**

| **Case Processing Summary** | | | | | | | | | |  |
| --- | --- | --- | --- | --- | --- | --- | --- | --- | --- | --- |
|  | Cases | | | | | | | | |  |
|  | Valid | | Missing | | | | Total | | |  |
|  | N | Percent | N | | Percent | | N | Percent | |  |
| Sound control | 10 | 100.0% | 0 | | 0.0% | | 10 | 100.0% | |  |
| SCO2 | 10 | 100.0% | 0 | | 0.0% | | 10 | 100.0% | |  |
| SMI | 10 | 100.0% | 0 | | 0.0% | | 10 | 100.0% | |  |
| SCO2Mi | 10 | 100.0% | 0 | | 0.0% | | 10 | 100.0% | |  |
| Demineralised | 10 | 100.0% | 0 | | 0.0% | | 10 | 100.0% | |  |
| DCO2 | 10 | 100.0% | 0 | | 0.0% | | 10 | 100.0% | |  |
| DMI | 10 | 100.0% | 0 | | 0.0% | | 10 | 100.0% | |  |
| DCO2MI | 10 | 100.0% | 0 | | 0.0% | | 10 | 100.0% | |  |
| **Descriptives** | | | | | | | | | | |
|  | | | | | | Statistic | | | Std. Error | |
| Sound control | Mean | | | | | 20200.00 | | | 442.217 | |
|  | 95% Confidence Interval for Mean | | | Lower Bound | | 19199.64 | | |  | |
|  |  |  |  | Upper Bound | | 21200.36 | | |  | |
|  | 5% Trimmed Mean | | | | | 20166.67 | | |  | |
|  | Median | | | | | 20000.00 | | |  | |
|  | Variance | | | | | 1955555.556 | | |  | |
|  | Std. Deviation | | | | | 1398.412 | | |  | |
|  | Minimum | | | | | 18000 | | |  | |
|  | Maximum | | | | | 23000 | | |  | |
|  | Range | | | | | 5000 | | |  | |
|  | Interquartile Range | | | | | 2000 | | |  | |
|  | Skewness | | | | | .475 | | | .687 | |
|  | Kurtosis | | | | | .813 | | | 1.334 | |
| SCO2 | Mean | | | | | 17200.00 | | | 512.076 | |
|  | 95% Confidence Interval for Mean | | | Lower Bound | | 16041.60 | | |  | |
|  |  |  |  | Upper Bound | | 18358.40 | | |  | |
|  | 5% Trimmed Mean | | | | | 17166.67 | | |  | |
|  | Median | | | | | 17000.00 | | |  | |
|  | Variance | | | | | 2622222.222 | | |  | |
|  | Std. Deviation | | | | | 1619.328 | | |  | |
|  | Minimum | | | | | 15000 | | |  | |
|  | Maximum | | | | | 20000 | | |  | |
|  | Range | | | | | 5000 | | |  | |
|  | Interquartile Range | | | | | 2500 | | |  | |
|  | Skewness | | | | | .188 | | | .687 | |
|  | Kurtosis | | | | | -.476 | | | 1.334 | |
| SMI | Mean | | | | | 14900.00 | | | 458.258 | |
|  | 95% Confidence Interval for Mean | | | Lower Bound | | 13863.35 | | |  | |
|  |  |  |  | Upper Bound | | 15936.65 | | |  | |
|  | 5% Trimmed Mean | | | | | 14888.89 | | |  | |
|  | Median | | | | | 15000.00 | | |  | |
|  | Variance | | | | | 2100000.000 | | |  | |
|  | Std. Deviation | | | | | 1449.138 | | |  | |
|  | Minimum | | | | | 13000 | | |  | |
|  | Maximum | | | | | 17000 | | |  | |
|  | Range | | | | | 4000 | | |  | |
|  | Interquartile Range | | | | | 2500 | | |  | |
|  | Skewness | | | | | .214 | | | .687 | |
|  | Kurtosis | | | | | -.987 | | | 1.334 | |
| SCO2Mi | Mean | | | | | 587.50 | | | 40.244 | |
|  | 95% Confidence Interval for Mean | | | Lower Bound | | 496.46 | | |  | |
|  |  |  |  | Upper Bound | | 678.54 | | |  | |
|  | 5% Trimmed Mean | | | | | 588.33 | | |  | |
|  | Median | | | | | 582.50 | | |  | |
|  | Variance | | | | | 16195.833 | | |  | |
|  | Std. Deviation | | | | | 127.263 | | |  | |
|  | Minimum | | | | | 400 | | |  | |
|  | Maximum | | | | | 760 | | |  | |
|  | Range | | | | | 360 | | |  | |
|  | Interquartile Range | | | | | 228 | | |  | |
|  | Skewness | | | | | -.317 | | | .687 | |
|  | Kurtosis | | | | | -1.114 | | | 1.334 | |
| Demineralised | Mean | | | | | 25700.00 | | | 856.997 | |
|  | 95% Confidence Interval for Mean | | | Lower Bound | | 23761.34 | | |  | |
|  |  |  |  | Upper Bound | | 27638.66 | | |  | |
|  | 5% Trimmed Mean | | | | | 25555.56 | | |  | |
|  | Median | | | | | 25000.00 | | |  | |
|  | Variance | | | | | 7344444.444 | | |  | |
|  | Std. Deviation | | | | | 2710.064 | | |  | |
|  | Minimum | | | | | 23000 | | |  | |
|  | Maximum | | | | | 31000 | | |  | |
|  | Range | | | | | 8000 | | |  | |
|  | Interquartile Range | | | | | 5000 | | |  | |
|  | Skewness | | | | | .773 | | | .687 | |
|  | Kurtosis | | | | | -.228 | | | 1.334 | |
| DCO2 | Mean | | | | | 15700.00 | | | 422.953 | |
|  | 95% Confidence Interval for Mean | | | Lower Bound | | 14743.21 | | |  | |
|  |  |  |  | Upper Bound | | 16656.79 | | |  | |
|  | 5% Trimmed Mean | | | | | 15777.78 | | |  | |
|  | Median | | | | | 16000.00 | | |  | |
|  | Variance | | | | | 1788888.889 | | |  | |
|  | Std. Deviation | | | | | 1337.494 | | |  | |
|  | Minimum | | | | | 13000 | | |  | |
|  | Maximum | | | | | 17000 | | |  | |
|  | Range | | | | | 4000 | | |  | |
|  | Interquartile Range | | | | | 2250 | | |  | |
|  | Skewness | | | | | -1.059 | | | .687 | |
|  | Kurtosis | | | | | .458 | | | 1.334 | |
| DMI | Mean | | | | | 12300.00 | | | 558.768 | |
|  | 95% Confidence Interval for Mean | | | Lower Bound | | 11035.98 | | |  | |
|  |  |  |  | Upper Bound | | 13564.02 | | |  | |
|  | 5% Trimmed Mean | | | | | 12222.22 | | |  | |
|  | Median | | | | | 12000.00 | | |  | |
|  | Variance | | | | | 3122222.222 | | |  | |
|  | Std. Deviation | | | | | 1766.981 | | |  | |
|  | Minimum | | | | | 10000 | | |  | |
|  | Maximum | | | | | 16000 | | |  | |
|  | Range | | | | | 6000 | | |  | |
|  | Interquartile Range | | | | | 2250 | | |  | |
|  | Skewness | | | | | .943 | | | .687 | |
|  | Kurtosis | | | | | .873 | | | 1.334 | |
| DCO2MI | Mean | | | | | 2600.00 | | | 305.505 | |
|  | 95% Confidence Interval for Mean | | | Lower Bound | | 1908.90 | | |  | |
|  |  |  |  | Upper Bound | | 3291.10 | | |  | |
|  | 5% Trimmed Mean | | | | | 2611.11 | | |  | |
|  | Median | | | | | 2500.00 | | |  | |
|  | Variance | | | | | 933333.333 | | |  | |
|  | Std. Deviation | | | | | 966.092 | | |  | |
|  | Minimum | | | | | 1000 | | |  | |
|  | Maximum | | | | | 4000 | | |  | |
|  | Range | | | | | 3000 | | |  | |
|  | Interquartile Range | | | | | 1250 | | |  | |
|  | Skewness | | | | | .111 | | | .687 | |
|  | Kurtosis | | | | | -.623 | | | 1.334 | |

| **Tests of Normality** | | | | | | |
| --- | --- | --- | --- | --- | --- | --- |
|  | Kolmogorov-Smirnov^a^ | | | Shapiro-Wilk | | |
|  | Statistic | df | Sig. | Statistic | df | Sig. |
| Sound control | .184 | 10 | .200^*^ | .945 | 10 | .609 |
| SCO2 | .151 | 10 | .200^*^ | .950 | 10 | .673 |
| SMI | .172 | 10 | .200^*^ | .917 | 10 | .330 |
| SCO2Mi | .166 | 10 | .200^*^ | .929 | 10 | .435 |
| Demineralised | .202 | 10 | .200^*^ | .891 | 10 | .174 |
| DCO2 | .289 | 10 | .018 | .855 | 10 | .067 |
| DMI | .169 | 10 | .200^*^ | .927 | 10 | .418 |
| DCO2MI | .233 | 10 | .133 | .904 | 10 | .245 |
| *. This is a lower bound of the true significance. | | | | | | |
| a. Lilliefors Significance Correction | | | | | | |

**Sound control**


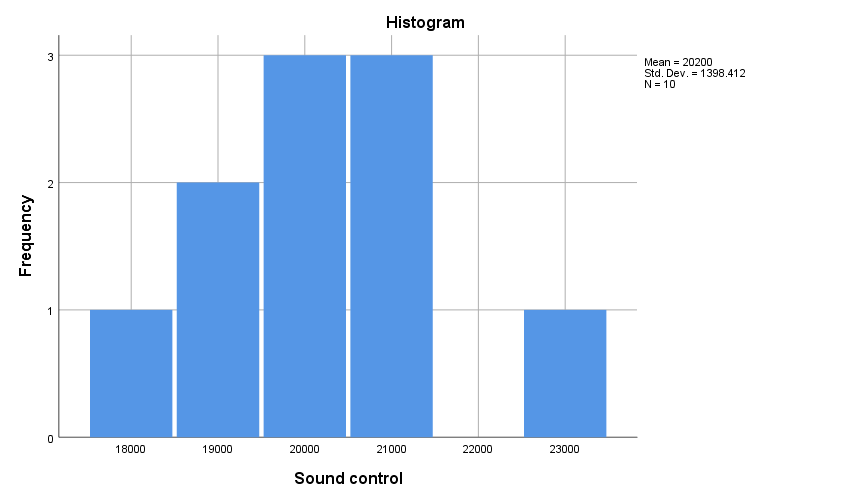


Sound control Stem-and-Leaf Plot

Frequency Stem & Leaf

1.00 18 . 0

2.00 19 . 00

3.00 20 . 000

3.00 21 . 000

.00 22 .

1.00 23 . 0

Stem width: 1000

Each leaf: 1 case(s)


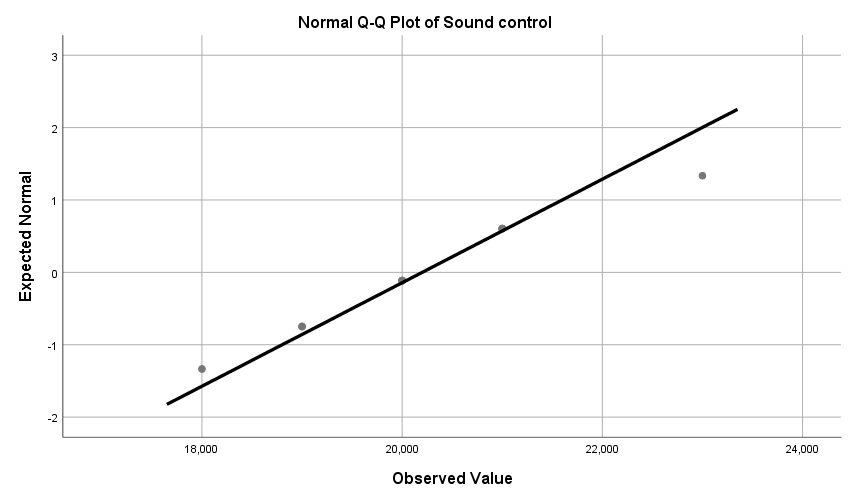


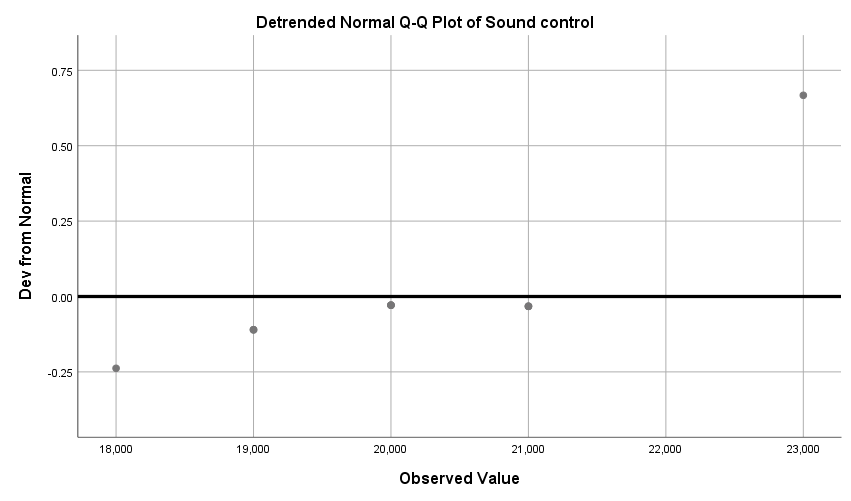


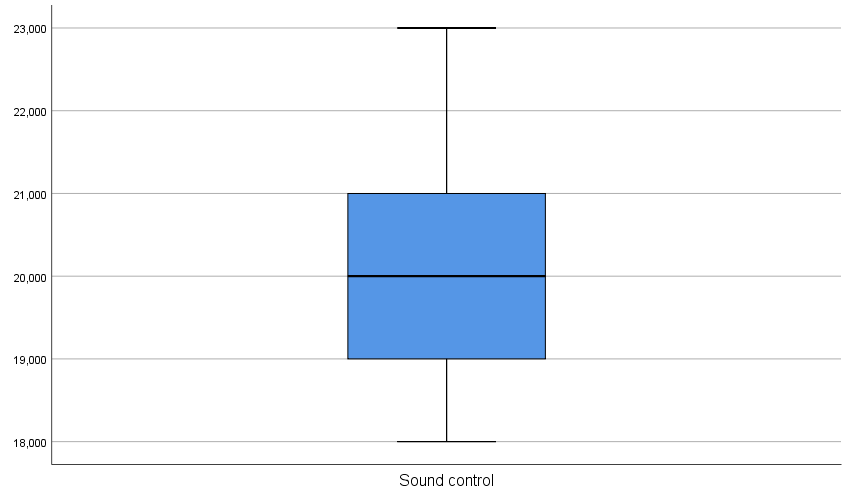


**SCO2**


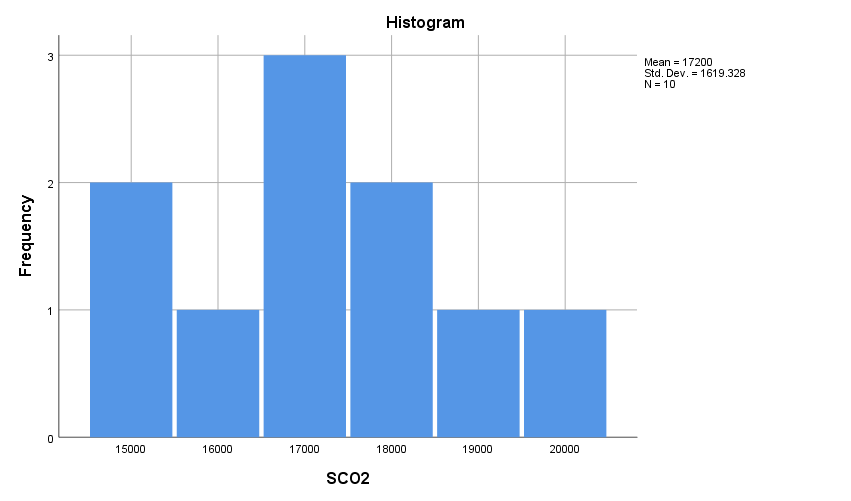


SCO2 Stem-and-Leaf Plot

Frequency Stem & Leaf

2.00 15 . 00

1.00 16 . 0

3.00 17 . 000

2.00 18 . 00

1.00 19 . 0

1.00 20 . 0

Stem width: 1000

Each leaf: 1 case(s)


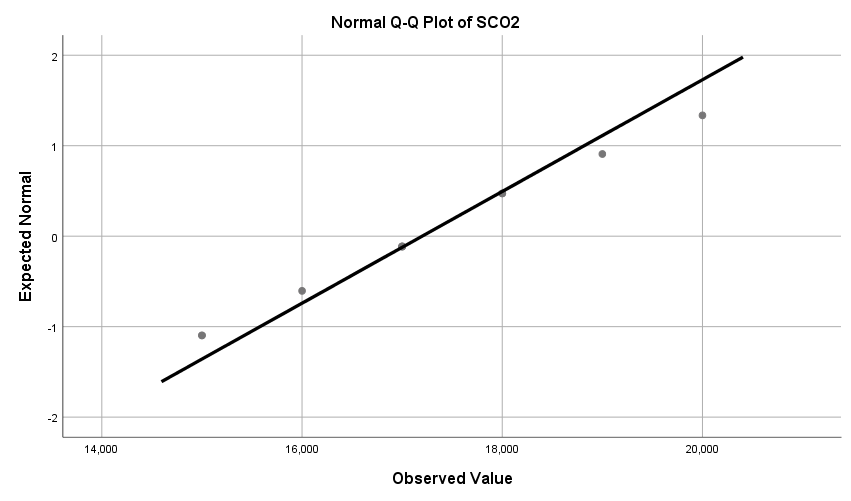


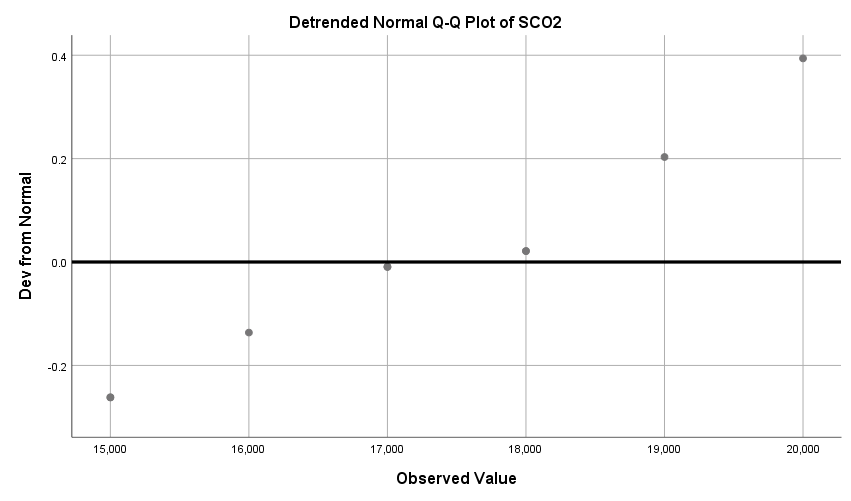


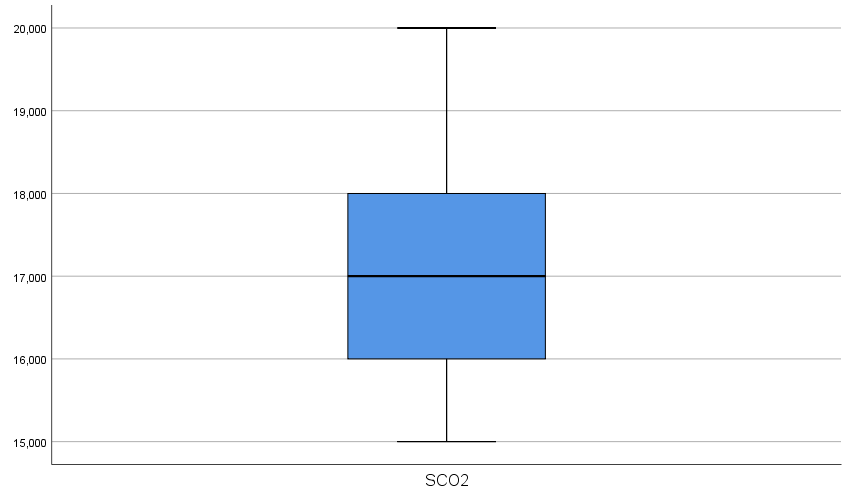


**SMI**


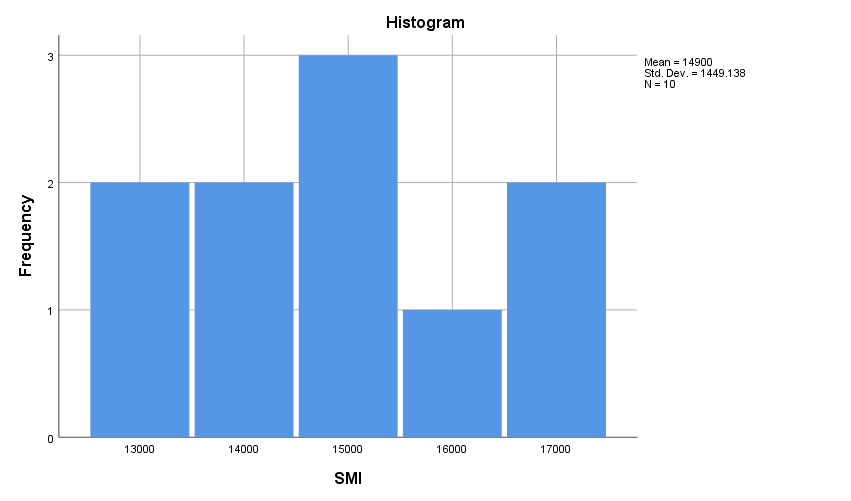


SMI Stem-and-Leaf Plot

Frequency Stem & Leaf

2.00 13 . 00

2.00 14 . 00

3.00 15 . 000

1.00 16 . 0

2.00 17 . 00

Stem width: 1000

Each leaf: 1 case(s)


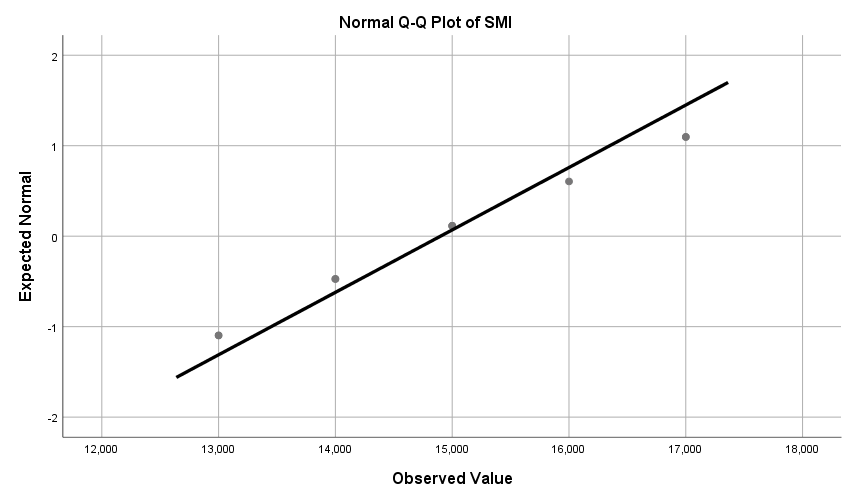


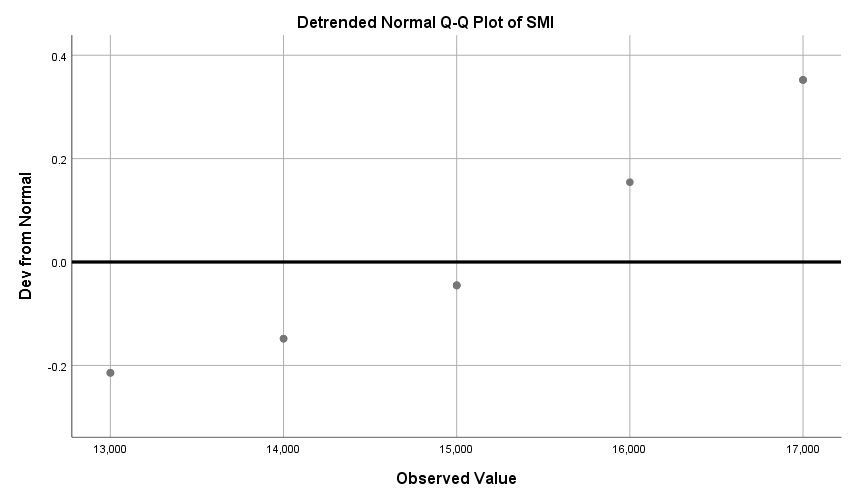


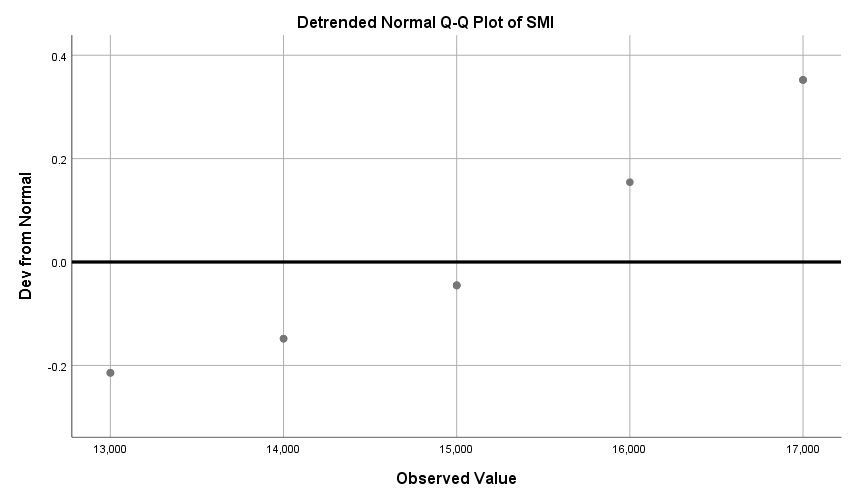


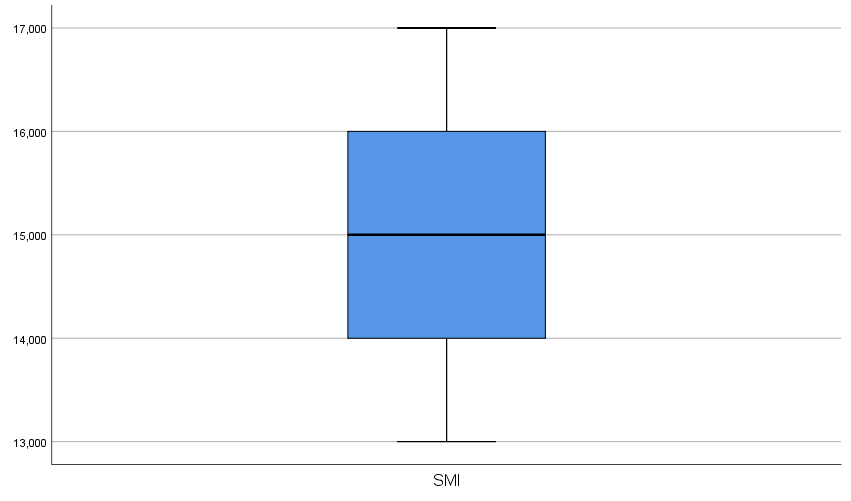


**SCO2Mi**


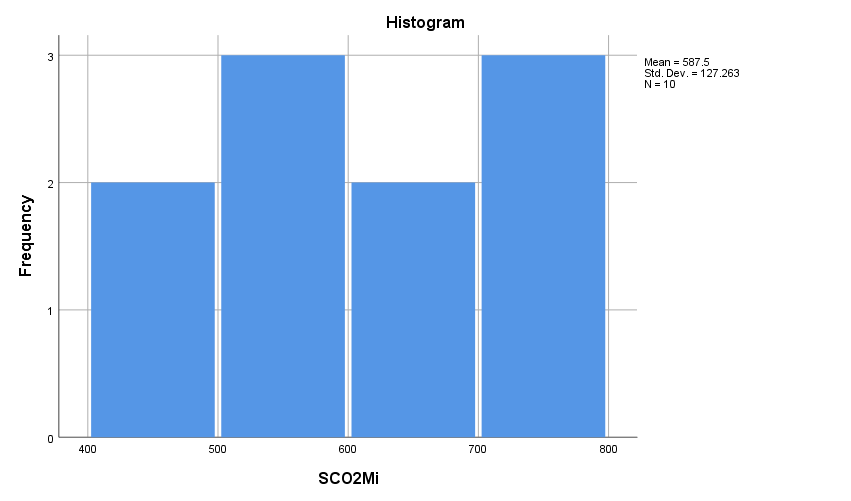


SCO2Mi Stem-and-Leaf Plot

Frequency Stem & Leaf

2.00 4 . 00

3.00 5 . 066

2.00 6 . 08

3.00 7 . 016

Stem width: 100

Each leaf: 1 case(s)


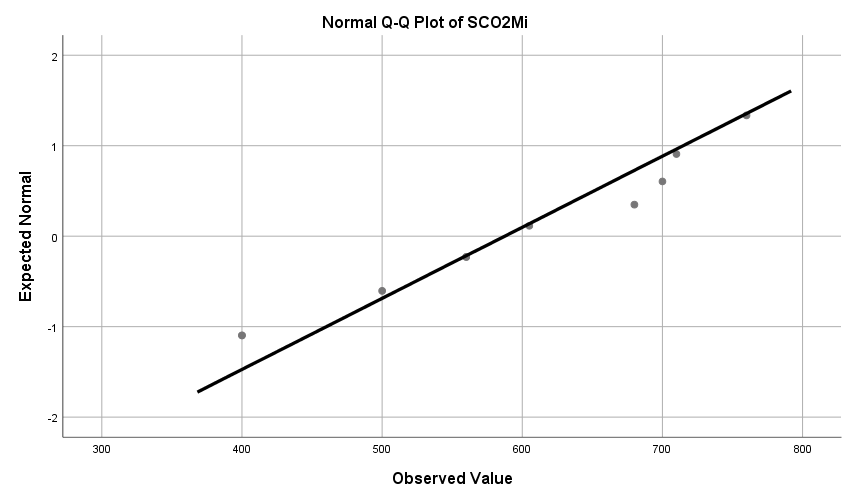


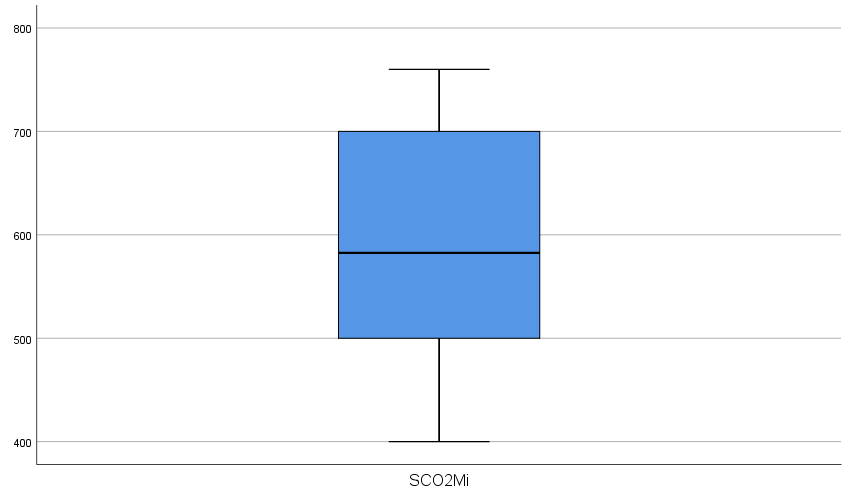


**Demineralised**


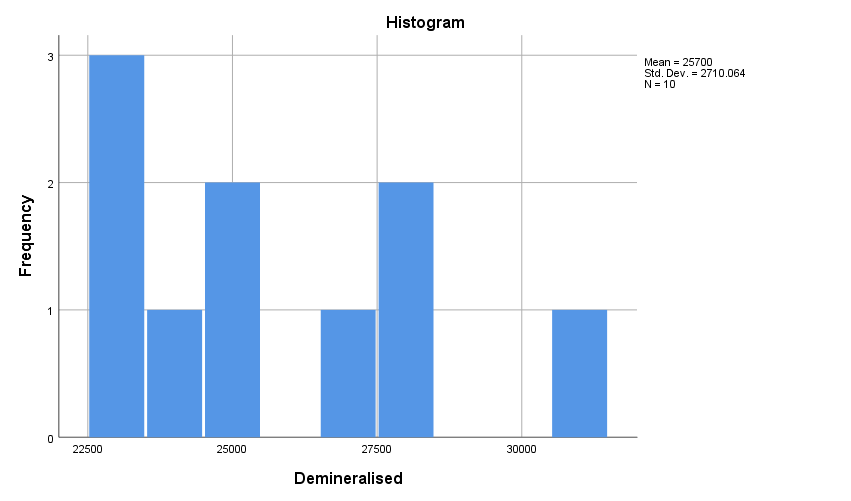


Demineralised Stem-and-Leaf Plot

Frequency Stem & Leaf

4.00 2 . 3334

5.00 2 . 55788

1.00 3 . 1

Stem width: 10000

Each leaf: 1 case(s)


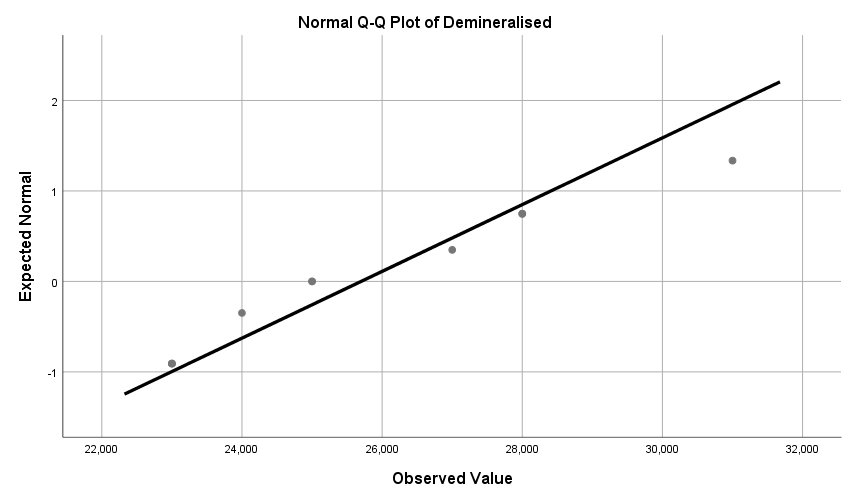


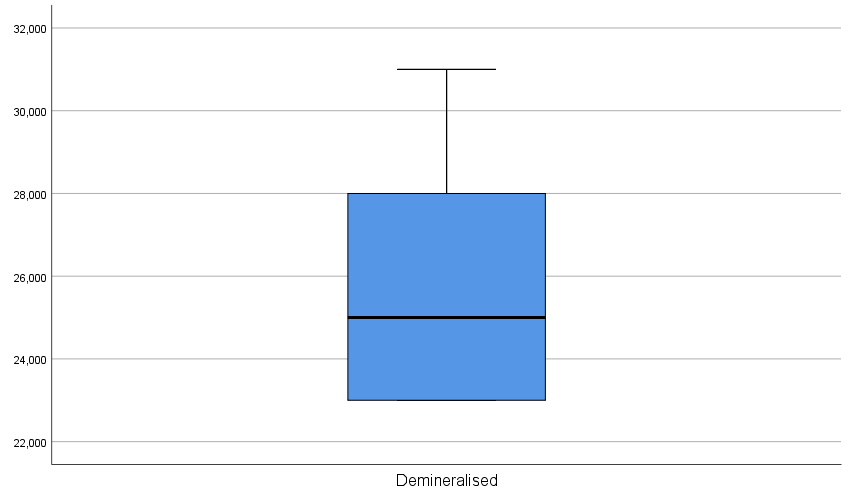


**DCO2**


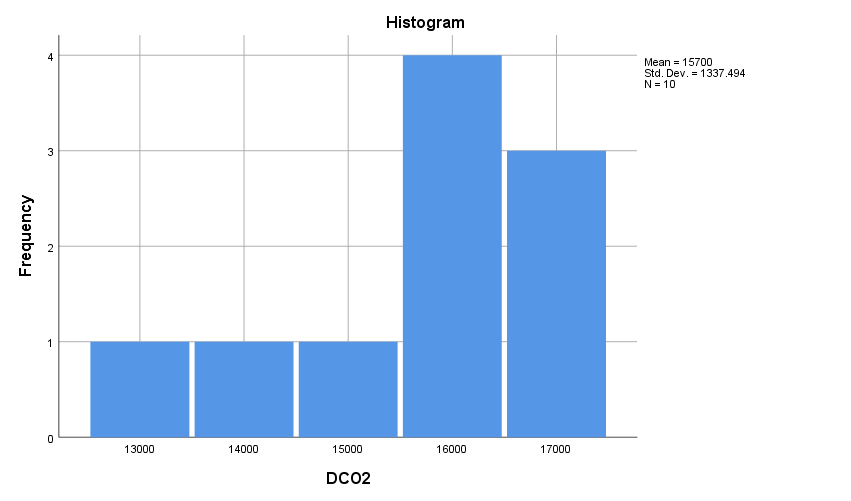


DCO2 Stem-and-Leaf Plot

Frequency Stem & Leaf

1.00 13 . 0

1.00 14 . 0

1.00 15 . 0

4.00 16 . 0000

3.00 17 . 000

Stem width: 1000

Each leaf: 1 case(s)


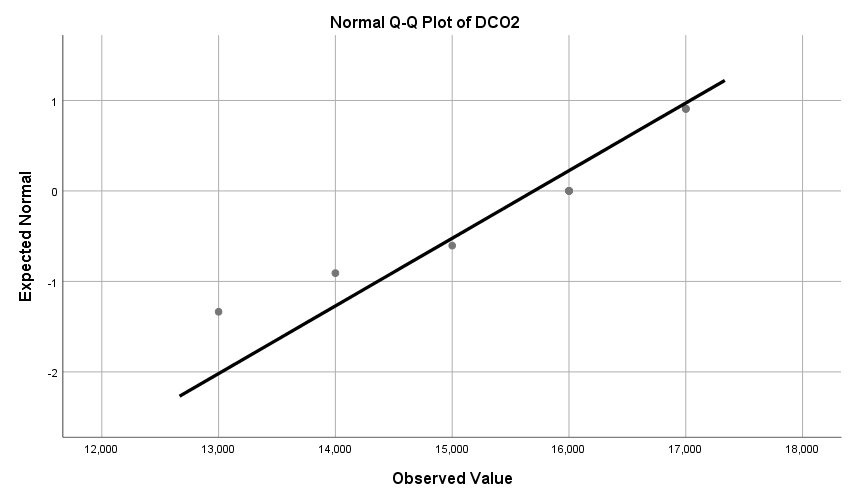


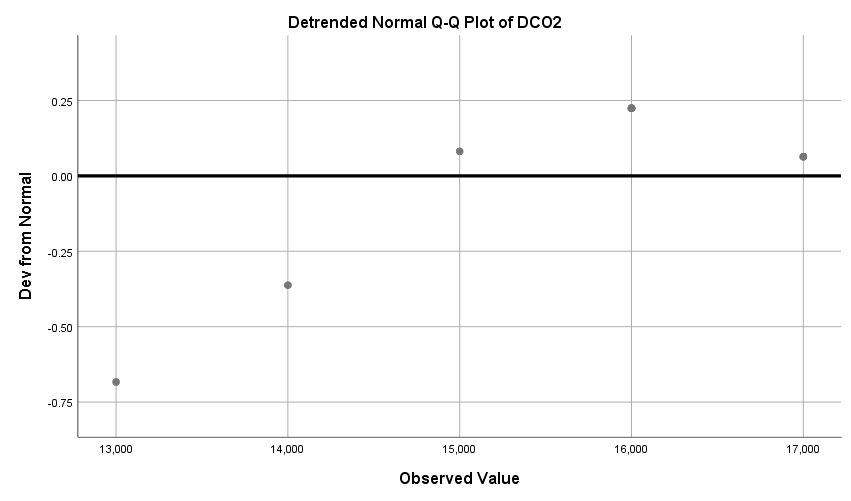


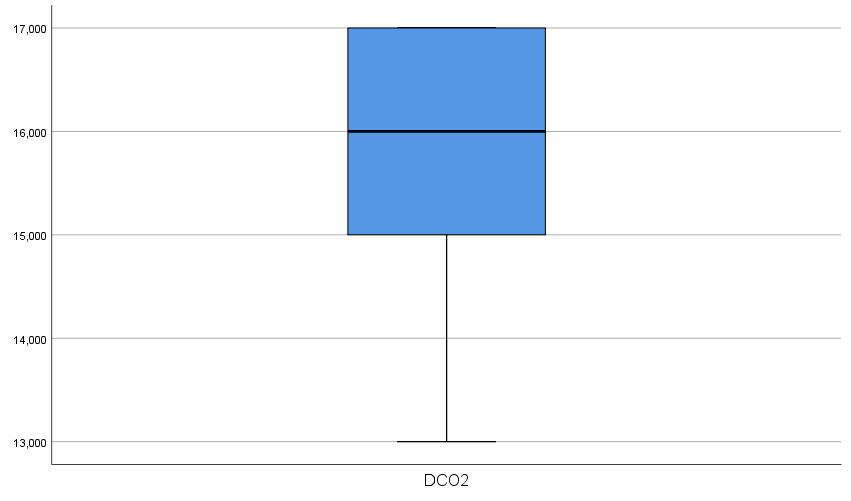


DMI Stem-and-Leaf Plot

Frequency Stem & Leaf

4.00 1 . 0111

4.00 1 . 2233

1.00 1 . 4

1.00 1 . 6

Stem width: 10000

Each leaf: 1 case(s)

DMI Stem-and-Leaf Plot

Frequency Stem & Leaf

4.00 1 . 0111

4.00 1 . 2233

1.00 1 . 4

1.00 1 . 6

Stem width: 10000

Each leaf: 1 case(s)


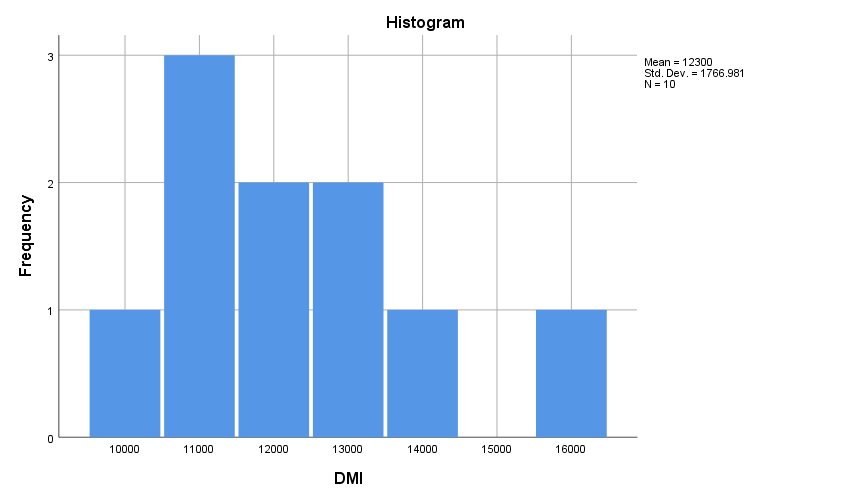


DMI Stem-and-Leaf Plot

Frequency Stem & Leaf

4.00 1 . 0111

4.00 1 . 2233

1.00 1 . 4

1.00 1 . 6

Stem width: 10000

Each leaf: 1 case(s)


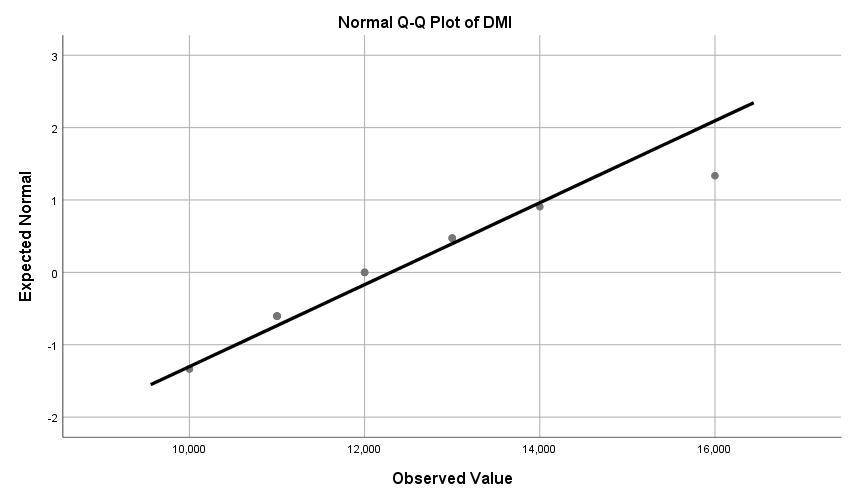


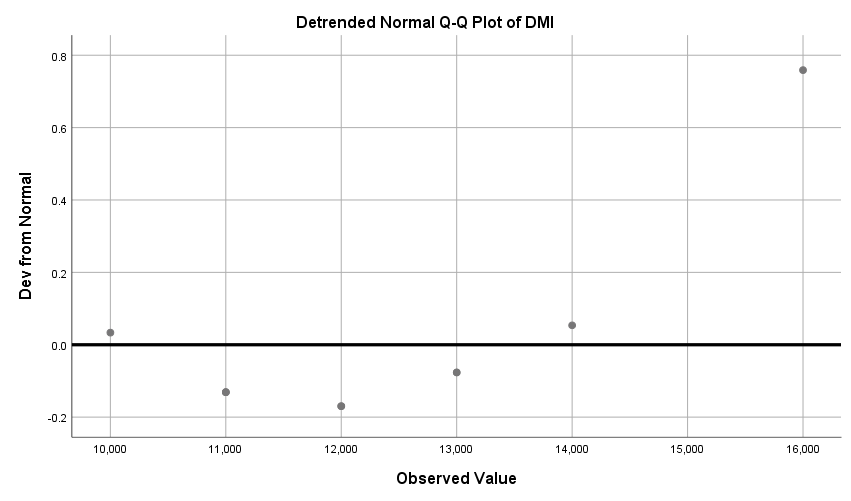


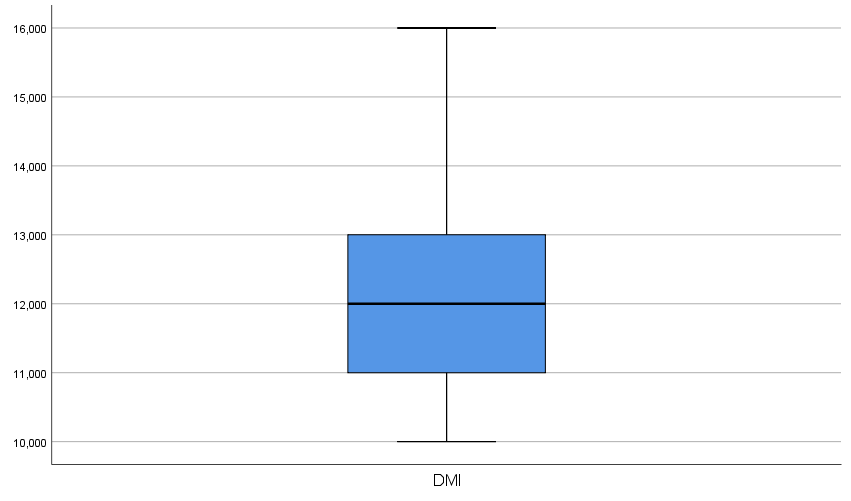


**DCO2MI**


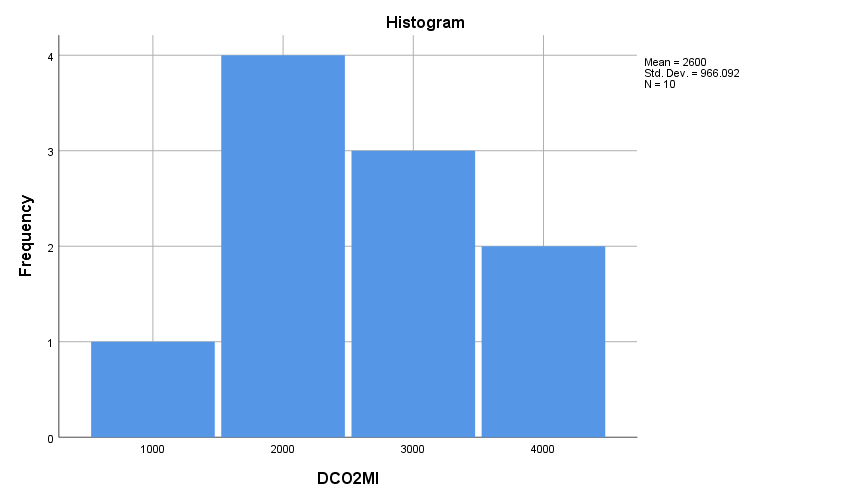


DCO2MI Stem-and-Leaf Plot

Frequency Stem & Leaf

1.00 1 . 0

4.00 2 . 0000

3.00 3 . 000

2.00 4 . 00

Stem width: 1000

Each leaf: 1 case(s)


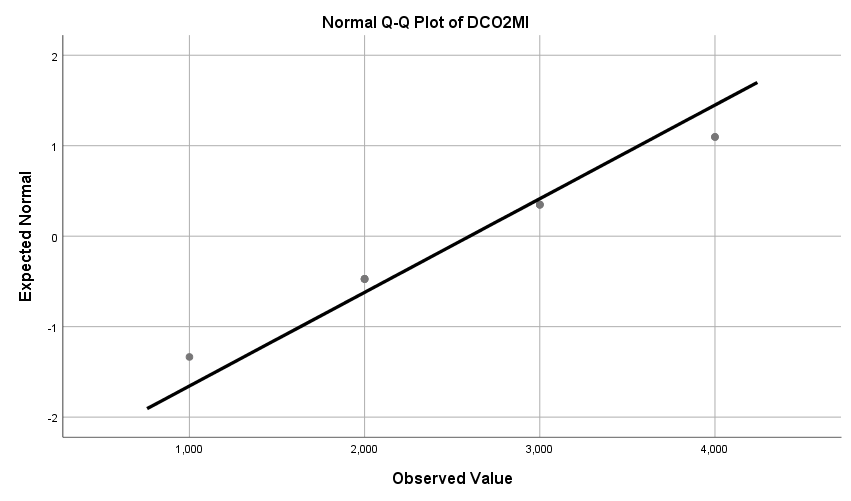


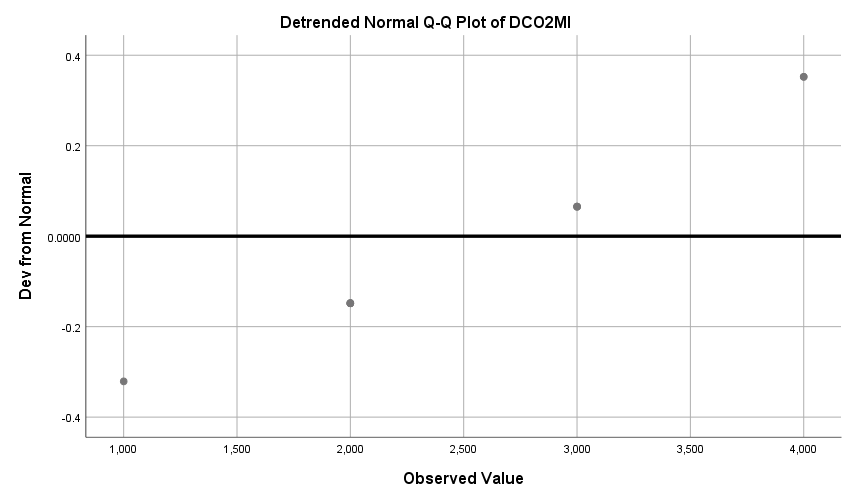


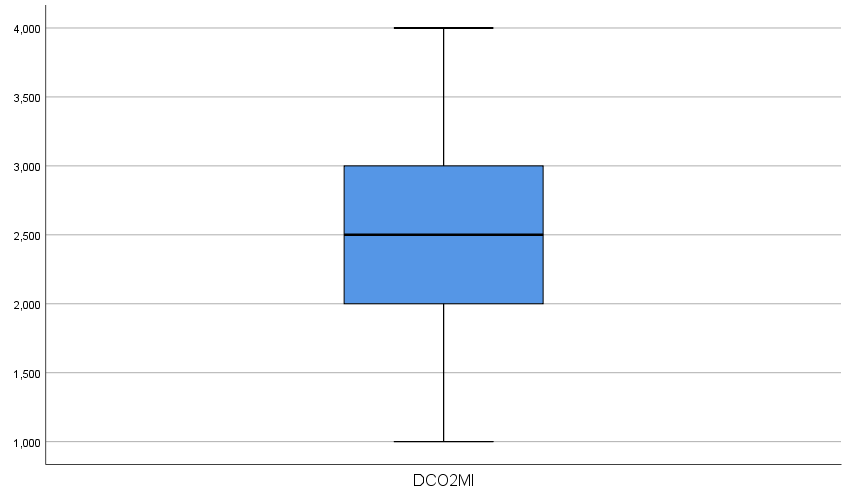


GET

FILE='C:\Users\apple\Desktop\Untitled2.sav'.

DATASET NAME DataSet4 WINDOW=FRONT.
