# Supplementary material for: The preventive/therapeutic effect of CO2 laser and MI Paste Plus® on intact and demineralized enamel against Streptococcus mutans (In Vitro Study)
Source: Heliyon. 2023 Sep 23;9(10):e20310. doi: 10.1016/j.heliyon.2023.e20310 (PMC10543189; doi:10.1016/j.heliyon.2023.e20310)
Supplement: Multimedia component 4 [file mmc4.docx]

Your temporary usage period for IBM SPSS Statistics will expire in 4782 days.

GET DATA

/TYPE=XLSX

/FILE='C:\Users\apple\Desktop\احصاء ضحى حميدي.xlsx'

/SHEET=name 'Sheet6'

/CELLRANGE=FULL

/READNAMES=ON

/DATATYPEMIN PERCENTAGE=95.0

/HIDDEN IGNORE=YES.

EXECUTE.

DATASET NAME DataSet1 WINDOW=FRONT.

T-TEST GROUPS=group(1 5)

/MISSING=ANALYSIS

/VARIABLES=treatment

/CRITERIA=CI(.95).

**T-Test**

[DataSet1]

| **Group Statistics** | | | | | |
| --- | --- | --- | --- | --- | --- |
|  | group | N | Mean | Std. Deviation | Std. Error Mean |
| treatment | 1 | 10 | 20200.00 | 1398.412 | 442.217 |
|  | 5 | 10 | 25700.00 | 2710.064 | 856.997 |

| **Independent Samples Test** | | | | | | | | | | |
| --- | --- | --- | --- | --- | --- | --- | --- | --- | --- | --- |
|  | | Levene's Test for Equality of Variances | | t-test for Equality of Means | | | | | | |
|  |  | F | Sig. | t | df | Sig. (2-tailed) | Mean Difference | Std. Error Difference | 95% Confidence Interval of the Difference | |
|  |  |  |  |  |  |  |  |  | Lower | Upper |
| treatment | Equal variances assumed | 5.707 | .028 | -5.703 | 18 | .000 | -5500.000 | 964.365 | -7526.056 | -3473.944 |
|  | Equal variances not assumed |  |  | -5.703 | 13.475 | .000 | -5500.000 | 964.365 | -7575.936 | -3424.064 |

T-TEST GROUPS=group(2 6)

/MISSING=ANALYSIS

/VARIABLES=treatment

/CRITERIA=CI(.95).

**T-Test**

| **Group Statistics** | | | | | |
| --- | --- | --- | --- | --- | --- |
|  | group | N | Mean | Std. Deviation | Std. Error Mean |
| treatment | 2 | 10 | 17200.00 | 1619.328 | 512.076 |
|  | 6 | 10 | 15700.00 | 1337.494 | 422.953 |

| **Independent Samples Test** | | | | | | | | | | |
| --- | --- | --- | --- | --- | --- | --- | --- | --- | --- | --- |
|  | | Levene's Test for Equality of Variances | | t-test for Equality of Means | | | | | | |
|  |  | F | Sig. | t | df | Sig. (2-tailed) | Mean Difference | Std. Error Difference | 95% Confidence Interval of the Difference | |
|  |  |  |  |  |  |  |  |  | Lower | Upper |
| treatment | Equal variances assumed | .313 | .583 | 2.258 | 18 | .037 | 1500.000 | 664.162 | 104.647 | 2895.353 |
|  | Equal variances not assumed |  |  | 2.258 | 17.380 | .037 | 1500.000 | 664.162 | 101.069 | 2898.931 |

T-TEST GROUPS=group(3 7)

/MISSING=ANALYSIS

/VARIABLES=treatment

/CRITERIA=CI(.95).

**T-Test**

| **Group Statistics** | | | | | |
| --- | --- | --- | --- | --- | --- |
|  | group | N | Mean | Std. Deviation | Std. Error Mean |
| treatment | 3 | 10 | 14900.00 | 1449.138 | 458.258 |
|  | 7 | 10 | 12300.00 | 1766.981 | 558.768 |

| **Independent Samples Test** | | | | | | | | | | |
| --- | --- | --- | --- | --- | --- | --- | --- | --- | --- | --- |
|  | | Levene's Test for Equality of Variances | | t-test for Equality of Means | | | | | | |
|  |  | F | Sig. | t | df | Sig. (2-tailed) | Mean Difference | Std. Error Difference | 95% Confidence Interval of the Difference | |
|  |  |  |  |  |  |  |  |  | Lower | Upper |
| treatment | Equal variances assumed | .325 | .576 | 3.598 | 18 | .002 | 2600.000 | 722.649 | 1081.770 | 4118.230 |
|  | Equal variances not assumed |  |  | 3.598 | 17.336 | .002 | 2600.000 | 722.649 | 1077.589 | 4122.411 |

T-TEST GROUPS=group(4 8)

/MISSING=ANALYSIS

/VARIABLES=treatment

/CRITERIA=CI(.95).

**T-Test**

| **Group Statistics** | | | | | |
| --- | --- | --- | --- | --- | --- |
|  | group | N | Mean | Std. Deviation | Std. Error Mean |
| treatment | 4 | 10 | 587.50 | 127.263 | 40.244 |
|  | 8 | 10 | 2600.00 | 966.092 | 305.505 |

| **Independent Samples Test** | | | | | | | | | | |
| --- | --- | --- | --- | --- | --- | --- | --- | --- | --- | --- |
|  | | Levene's Test for Equality of Variances | | t-test for Equality of Means | | | | | | |
|  |  | F | Sig. | t | df | Sig. (2-tailed) | Mean Difference | Std. Error Difference | 95% Confidence Interval of the Difference | |
|  |  |  |  |  |  |  |  |  | Lower | Upper |
| treatment | Equal variances assumed | 21.416 | .000 | -6.531 | 18 | .000 | -2012.500 | 308.144 | -2659.887 | -1365.113 |
|  | Equal variances not assumed |  |  | -6.531 | 9.312 | .000 | -2012.500 | 308.144 | -2706.024 | -1318.976 |
