# Supplementary material for: The preventive/therapeutic effect of CO2 laser and MI Paste Plus® on intact and demineralized enamel against Streptococcus mutans (In Vitro Study)
Source: Heliyon. 2023 Sep 23;9(10):e20310. doi: 10.1016/j.heliyon.2023.e20310 (PMC10543189; doi:10.1016/j.heliyon.2023.e20310)

Your temporary usage period for IBM SPSS Statistics will expire in 4782 days.

GET DATA

/TYPE=XLSX

/FILE='C:\Users\apple\Desktop\احصاء ضحى حميدي.xlsx'

/SHEET=name 'Sheet4'

/CELLRANGE=FULL

/READNAMES=ON

/DATATYPEMIN PERCENTAGE=95.0

/HIDDEN IGNORE=YES.

EXECUTE.

DATASET NAME DataSet1 WINDOW=FRONT.

ONEWAY treatment BY group

/STATISTICS DESCRIPTIVES HOMOGENEITY

/PLOT MEANS

/MISSING ANALYSIS.

**Oneway**

[DataSet1]

| **Descriptives** | | | | | | | | |
| --- | --- | --- | --- | --- | --- | --- | --- | --- |
| treatment | | | | | | | | |
|  | N | Mean | Std. Deviation | Std. Error | 95% Confidence Interval for Mean | | Minimum | Maximum |
|  |  |  |  |  | Lower Bound | Upper Bound |  |  |
| 5 | 10 | 25700.00 | 2710.064 | 856.997 | 23761.34 | 27638.66 | 23000 | 31000 |
| 6 | 10 | 15700.00 | 1337.494 | 422.953 | 14743.21 | 16656.79 | 13000 | 17000 |
| 7 | 10 | 12300.00 | 1766.981 | 558.768 | 11035.98 | 13564.02 | 10000 | 16000 |
| 8 | 10 | 2600.00 | 966.092 | 305.505 | 1908.90 | 3291.10 | 1000 | 4000 |
| Total | 40 | 14075.00 | 8540.664 | 1350.398 | 11343.56 | 16806.44 | 1000 | 31000 |

| **Test of Homogeneity of Variances** | | | | | |
| --- | --- | --- | --- | --- | --- |
|  | | Levene Statistic | df1 | df2 | Sig. |
| treatment | Based on Mean | 4.347 | 3 | 36 | .010 |
|  | Based on Median | 2.514 | 3 | 36 | .074 |
|  | Based on Median and with adjusted df | 2.514 | 3 | 23.594 | .083 |
|  | Based on trimmed mean | 3.925 | 3 | 36 | .016 |

| **ANOVA** | | | | | |
| --- | --- | --- | --- | --- | --- |
| treatment | | | | | |
|  | Sum of Squares | df | Mean Square | F | Sig. |
| Between Groups | 2726075000.000 | 3 | 908691666.667 | 275.593 | .000 |
| Within Groups | 118700000.000 | 36 | 3297222.222 |  |  |
| Total | 2844775000.000 | 39 |  |  |  |

**Means Plots**


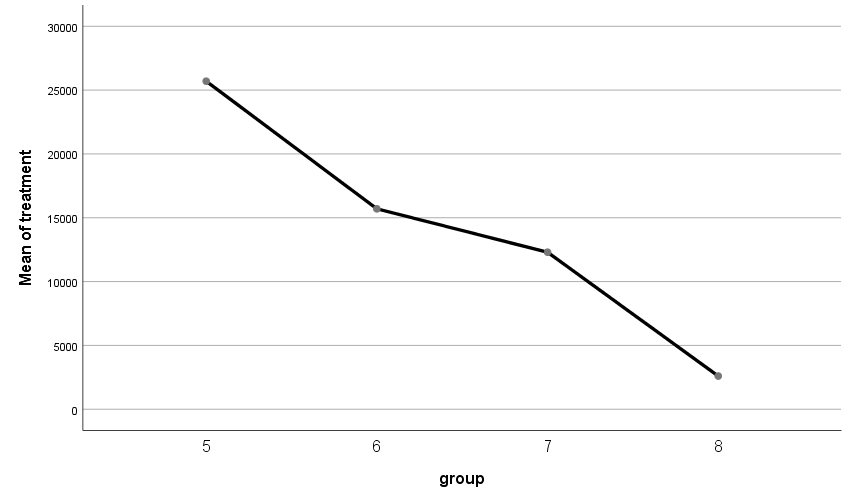

Supplement: Multimedia component 5 [file mmc5.docx]
