# Supplementary material for: The preventive/therapeutic effect of CO2 laser and MI Paste Plus® on intact and demineralized enamel against Streptococcus mutans (In Vitro Study)
Source: Heliyon. 2023 Sep 23;9(10):e20310. doi: 10.1016/j.heliyon.2023.e20310 (PMC10543189; doi:10.1016/j.heliyon.2023.e20310)

GET DATA

/TYPE=XLSX

/FILE='C:\Users\apple\Desktop\احصاء ضحى حميدي.xlsx'

/SHEET=name 'Sheet4'

/CELLRANGE=FULL

/READNAMES=ON

/DATATYPEMIN PERCENTAGE=95.0

/HIDDEN IGNORE=YES.

EXECUTE.

DATASET NAME DataSet1 WINDOW=FRONT.

ONEWAY treatment BY group

/STATISTICS DESCRIPTIVES HOMOGENEITY

/PLOT MEANS

/MISSING ANALYSIS

/POSTHOC=TUKEY ALPHA(0.05).

**Oneway**

[DataSet1]

| **Descriptives** | | | | | | | | |
| --- | --- | --- | --- | --- | --- | --- | --- | --- |
| treatment | | | | | | | | |
|  | N | Mean | Std. Deviation | Std. Error | 95% Confidence Interval for Mean | | Minimum | Maximum |
|  |  |  |  |  | Lower Bound | Upper Bound |  |  |
| 5 | 10 | 25700.00 | 2710.064 | 856.997 | 23761.34 | 27638.66 | 23000 | 31000 |
| 6 | 10 | 15700.00 | 1337.494 | 422.953 | 14743.21 | 16656.79 | 13000 | 17000 |
| 7 | 10 | 12300.00 | 1766.981 | 558.768 | 11035.98 | 13564.02 | 10000 | 16000 |
| 8 | 10 | 2600.00 | 966.092 | 305.505 | 1908.90 | 3291.10 | 1000 | 4000 |
| Total | 40 | 14075.00 | 8540.664 | 1350.398 | 11343.56 | 16806.44 | 1000 | 31000 |

| **Test of Homogeneity of Variances** | | | | | | | | | | | |
| --- | --- | --- | --- | --- | --- | --- | --- | --- | --- | --- | --- |
|  | | | | Levene Statistic | | df1 | | df2 | | Sig. | |
| treatment | Based on Mean | | | 4.347 | | 3 | | 36 | | .010 | |
|  | Based on Median | | | 2.514 | | 3 | | 36 | | .074 | |
|  | Based on Median and with adjusted df | | | 2.514 | | 3 | | 23.594 | | .083 | |
|  | Based on trimmed mean | | | 3.925 | | 3 | | 36 | | .016 | |
| **ANOVA** | | | | | | | | | | |  |
| treatment | | | | | | | | | | |  |
|  | | Sum of Squares | df | | Mean Square | | F | | Sig. | |  |
| Between Groups | | 2726075000.000 | 3 | | 908691666.667 | | 275.593 | | .000 | |  |
| Within Groups | | 118700000.000 | 36 | | 3297222.222 | |  | |  | |  |
| Total | | 2844775000.000 | 39 | |  | |  | |  | |  |

**Post Hoc Tests**

| **Multiple Comparisons** | | | | | | |
| --- | --- | --- | --- | --- | --- | --- |
| Dependent Variable: treatment | | | | | | |
| Tukey HSD | | | | | | |
| (I) group | (J) group | Mean Difference (I-J) | Std. Error | Sig. | 95% Confidence Interval | |
|  |  |  |  |  | Lower Bound | Upper Bound |
| 5 | 6 | 10000.000^*^ | 812.062 | .000 | 7812.93 | 12187.07 |
|  | 7 | 13400.000^*^ | 812.062 | .000 | 11212.93 | 15587.07 |
|  | 8 | 23100.000^*^ | 812.062 | .000 | 20912.93 | 25287.07 |
| 6 | 5 | -10000.000^*^ | 812.062 | .000 | -12187.07 | -7812.93 |
|  | 7 | 3400.000^*^ | 812.062 | .001 | 1212.93 | 5587.07 |
|  | 8 | 13100.000^*^ | 812.062 | .000 | 10912.93 | 15287.07 |
| 7 | 5 | -13400.000^*^ | 812.062 | .000 | -15587.07 | -11212.93 |
|  | 6 | -3400.000^*^ | 812.062 | .001 | -5587.07 | -1212.93 |
|  | 8 | 9700.000^*^ | 812.062 | .000 | 7512.93 | 11887.07 |
| 8 | 5 | -23100.000^*^ | 812.062 | .000 | -25287.07 | -20912.93 |
|  | 6 | -13100.000^*^ | 812.062 | .000 | -15287.07 | -10912.93 |
|  | 7 | -9700.000^*^ | 812.062 | .000 | -11887.07 | -7512.93 |
| *. The mean difference is significant at the 0.05 level. | | | | | | |

**Homogeneous Subsets**

| **treatment** | | | | | |
| --- | --- | --- | --- | --- | --- |
| Tukey HSD^a^ | | | | | |
| group | N | Subset for alpha = 0.05 | | | |
|  |  | 1 | 2 | 3 | 4 |
| 8 | 10 | 2600.00 |  |  |  |
| 7 | 10 |  | 12300.00 |  |  |
| 6 | 10 |  |  | 15700.00 |  |
| 5 | 10 |  |  |  | 25700.00 |
| Sig. |  | 1.000 | 1.000 | 1.000 | 1.000 |
| Means for groups in homogeneous subsets are displayed. | | | | | |
| a. Uses Harmonic Mean Sample Size = 10.000. | | | | | |

**Means Plots**


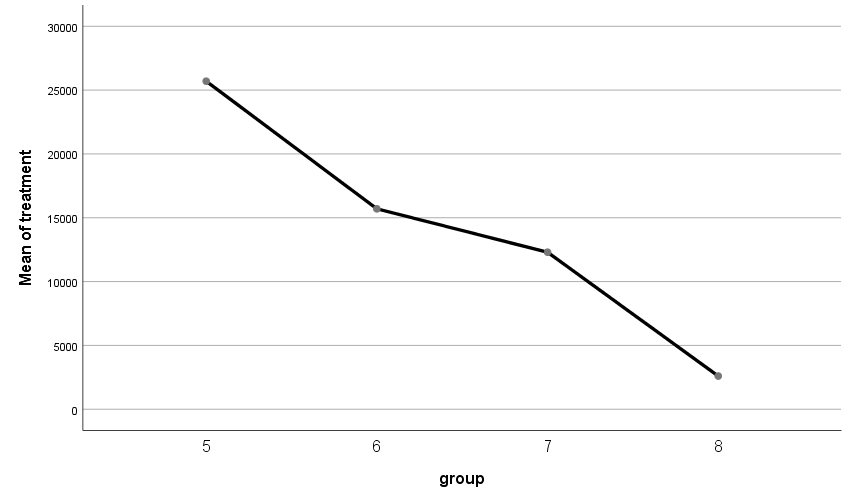

Supplement: Multimedia component 7 [file mmc7.docx]
