# Supplementary material for: The preventive/therapeutic effect of CO2 laser and MI Paste Plus® on intact and demineralized enamel against Streptococcus mutans (In Vitro Study)
Source: Heliyon. 2023 Sep 23;9(10):e20310. doi: 10.1016/j.heliyon.2023.e20310 (PMC10543189; doi:10.1016/j.heliyon.2023.e20310)

Your temporary usage period for IBM SPSS Statistics will expire in 4782 days.

GET DATA

/TYPE=XLSX

/FILE='C:\Users\apple\Desktop\احصاء ضحى حميدي.xlsx'

/SHEET=name 'Sheet3'

/CELLRANGE=FULL

/READNAMES=ON

/DATATYPEMIN PERCENTAGE=95.0

/HIDDEN IGNORE=YES.

EXECUTE.

DATASET NAME DataSet1 WINDOW=FRONT.

ONEWAY treatment BY group

/STATISTICS DESCRIPTIVES HOMOGENEITY

/PLOT MEANS

/MISSING ANALYSIS

/POSTHOC=TUKEY ALPHA(0.05).

**Oneway**

[DataSet1]

| **Descriptives** | | | | | | | | |
| --- | --- | --- | --- | --- | --- | --- | --- | --- |
| treatment | | | | | | | | |
|  | N | Mean | Std. Deviation | Std. Error | 95% Confidence Interval for Mean | | Minimum | Maximum |
|  |  |  |  |  | Lower Bound | Upper Bound |  |  |
| 1 | 10 | 20200.00 | 1398.412 | 442.217 | 19199.64 | 21200.36 | 18000 | 23000 |
| 2 | 10 | 17200.00 | 1619.328 | 512.076 | 16041.60 | 18358.40 | 15000 | 20000 |
| 3 | 10 | 14900.00 | 1449.138 | 458.258 | 13863.35 | 15936.65 | 13000 | 17000 |
| 4 | 10 | 587.50 | 127.263 | 40.244 | 496.46 | 678.54 | 400 | 760 |
| Total | 40 | 13221.88 | 7729.196 | 1222.093 | 10749.96 | 15693.79 | 400 | 23000 |

| **Test of Homogeneity of Variances** | | | | | |
| --- | --- | --- | --- | --- | --- |
|  | | Levene Statistic | df1 | df2 | Sig. |
| treatment | Based on Mean | 4.573 | 3 | 36 | .008 |
|  | Based on Median | 3.740 | 3 | 36 | .019 |
|  | Based on Median and with adjusted df | 3.740 | 3 | 26.596 | .023 |
|  | Based on trimmed mean | 4.482 | 3 | 36 | .009 |

| **ANOVA** | | | | | |
| --- | --- | --- | --- | --- | --- |
| treatment | | | | | |
|  | Sum of Squares | df | Mean Square | F | Sig. |
| Between Groups | 2269632421.875 | 3 | 756544140.625 | 452.075 | .000 |
| Within Groups | 60245762.500 | 36 | 1673493.403 |  |  |
| Total | 2329878184.375 | 39 |  |  |  |

**Post Hoc Tests**

| **Multiple Comparisons** | | | | | | |
| --- | --- | --- | --- | --- | --- | --- |
| Dependent Variable: treatment | | | | | | |
| Tukey HSD | | | | | | |
| (I) group | (J) group | Mean Difference (I-J) | Std. Error | Sig. | 95% Confidence Interval | |
|  |  |  |  |  | Lower Bound | Upper Bound |
| 1 | 2 | 3000.000^*^ | 578.531 | .000 | 1441.88 | 4558.12 |
|  | 3 | 5300.000^*^ | 578.531 | .000 | 3741.88 | 6858.12 |
|  | 4 | 19612.500^*^ | 578.531 | .000 | 18054.38 | 21170.62 |
| 2 | 1 | -3000.000^*^ | 578.531 | .000 | -4558.12 | -1441.88 |
|  | 3 | 2300.000^*^ | 578.531 | .002 | 741.88 | 3858.12 |
|  | 4 | 16612.500^*^ | 578.531 | .000 | 15054.38 | 18170.62 |
| 3 | 1 | -5300.000^*^ | 578.531 | .000 | -6858.12 | -3741.88 |
|  | 2 | -2300.000^*^ | 578.531 | .002 | -3858.12 | -741.88 |
|  | 4 | 14312.500^*^ | 578.531 | .000 | 12754.38 | 15870.62 |
| 4 | 1 | -19612.500^*^ | 578.531 | .000 | -21170.62 | -18054.38 |
|  | 2 | -16612.500^*^ | 578.531 | .000 | -18170.62 | -15054.38 |
|  | 3 | -14312.500^*^ | 578.531 | .000 | -15870.62 | -12754.38 |
| *. The mean difference is significant at the 0.05 level. | | | | | | |

**Homogeneous Subsets**

| **treatment** | | | | | |
| --- | --- | --- | --- | --- | --- |
| Tukey HSD^a^ | | | | | |
| group | N | Subset for alpha = 0.05 | | | |
|  |  | 1 | 2 | 3 | 4 |
| 4 | 10 | 587.50 |  |  |  |
| 3 | 10 |  | 14900.00 |  |  |
| 2 | 10 |  |  | 17200.00 |  |
| 1 | 10 |  |  |  | 20200.00 |
| Sig. |  | 1.000 | 1.000 | 1.000 | 1.000 |
| Means for groups in homogeneous subsets are displayed. | | | | | |
| a. Uses Harmonic Mean Sample Size = 10.000. | | | | | |

**Means Plots**


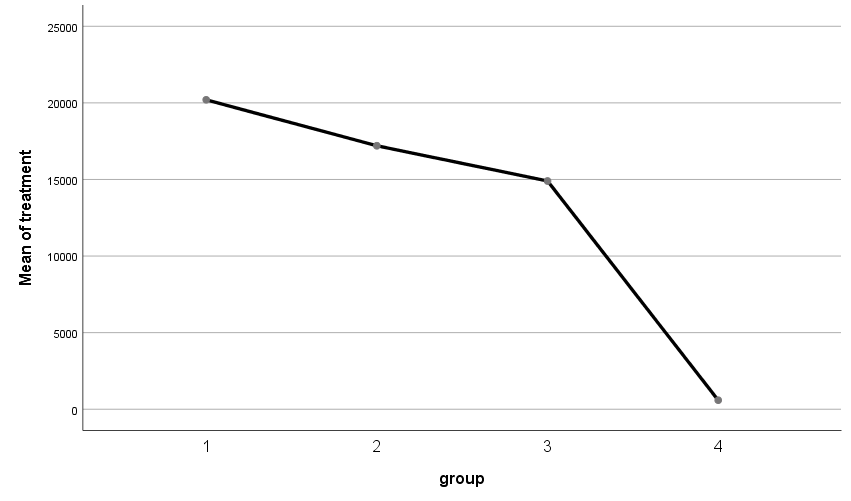

Supplement: Multimedia component 8 [file mmc8.docx]
