# Supplementary material for: The preventive/therapeutic effect of CO2 laser and MI Paste Plus® on intact and demineralized enamel against Streptococcus mutans (In Vitro Study)
Source: Heliyon. 2023 Sep 23;9(10):e20310. doi: 10.1016/j.heliyon.2023.e20310 (PMC10543189; doi:10.1016/j.heliyon.2023.e20310)

## Slide 1
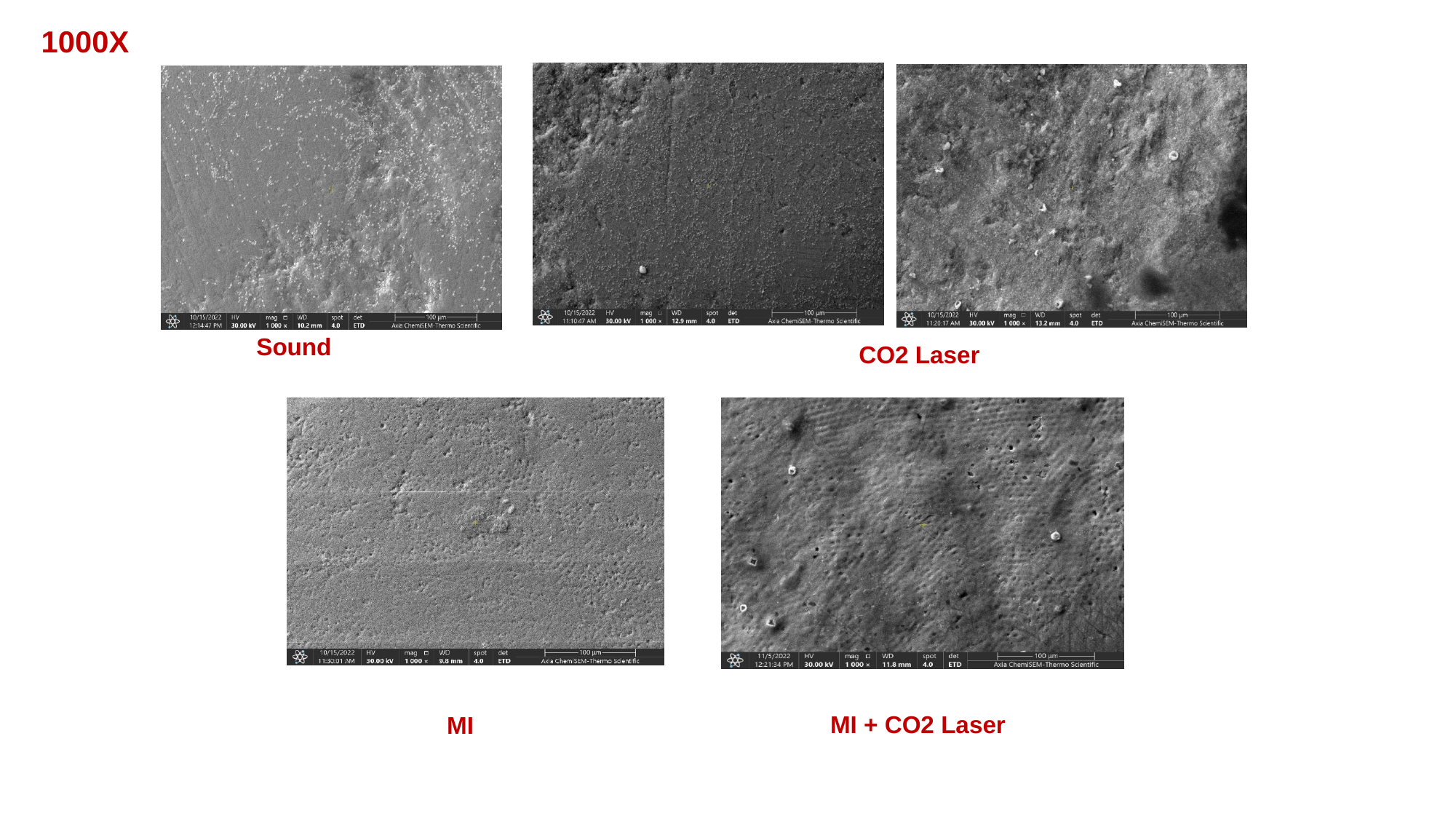

1000X
Sound
CO2 Laser
MI + CO2 Laser
MI

## Slide 2
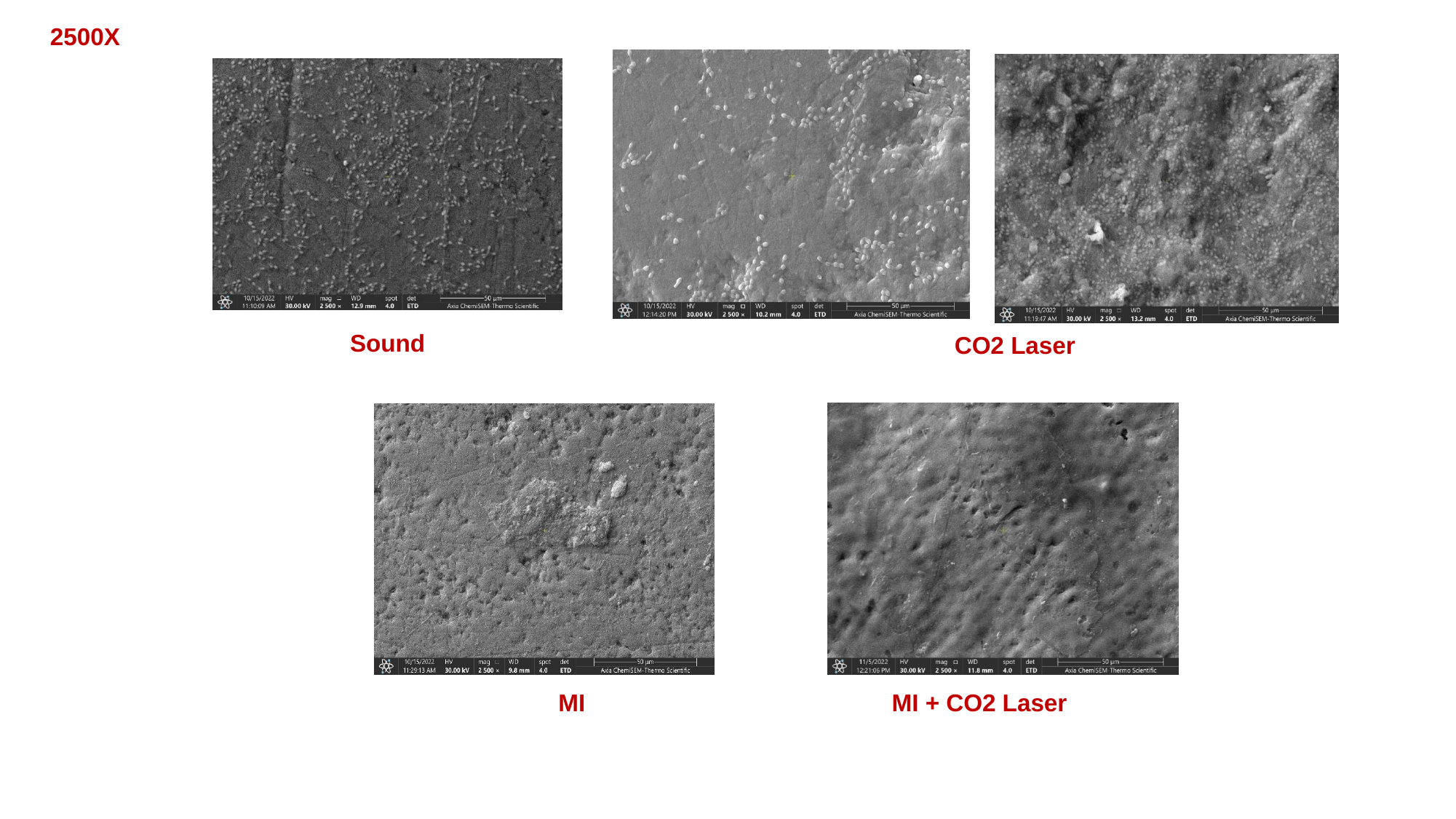

2500X
Sound
CO2 Laser
MI
MI + CO2 Laser

## Slide 3
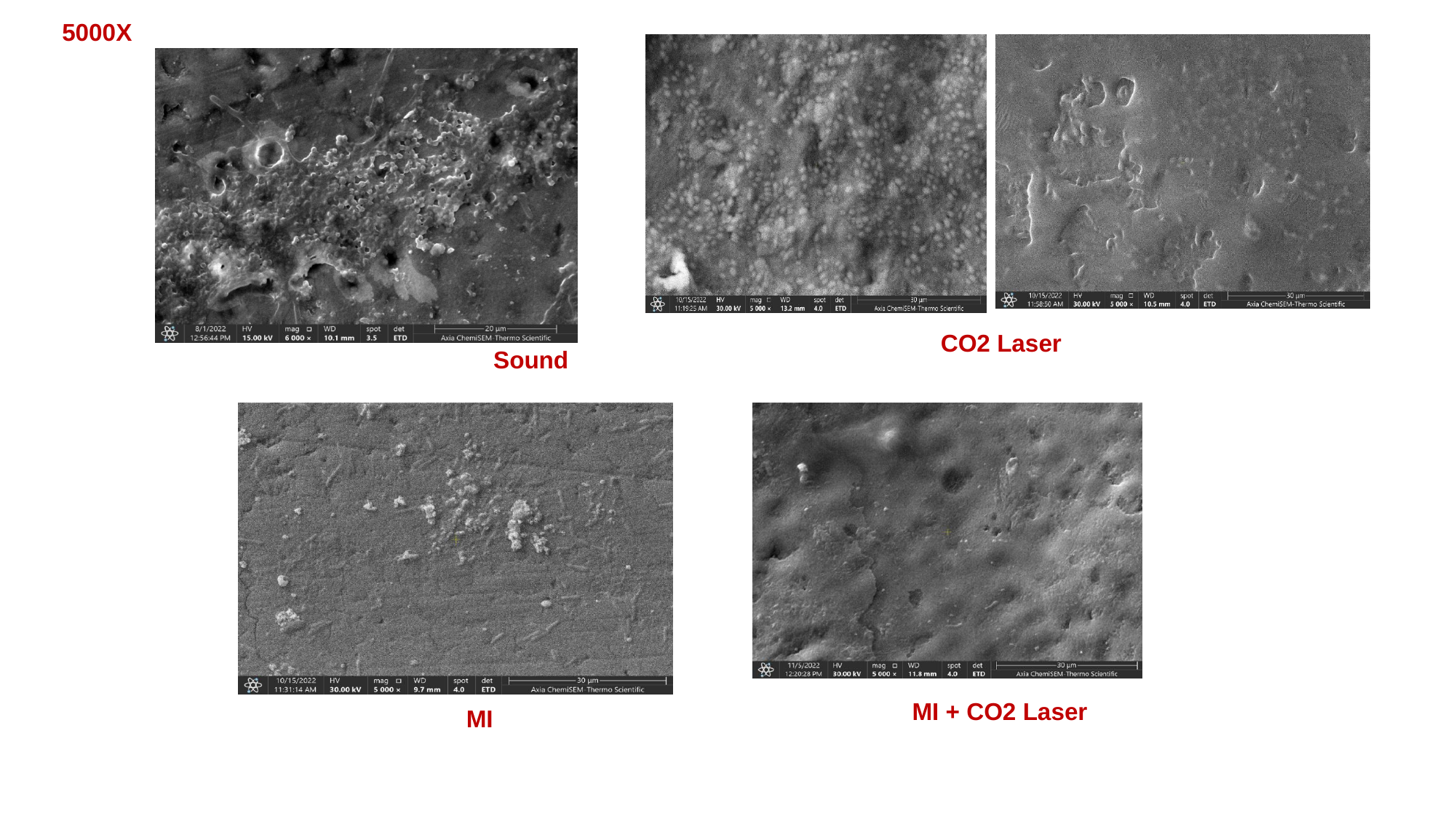

5000X
CO2 Laser
Sound
MI + CO2 Laser
MI

## Slide 4
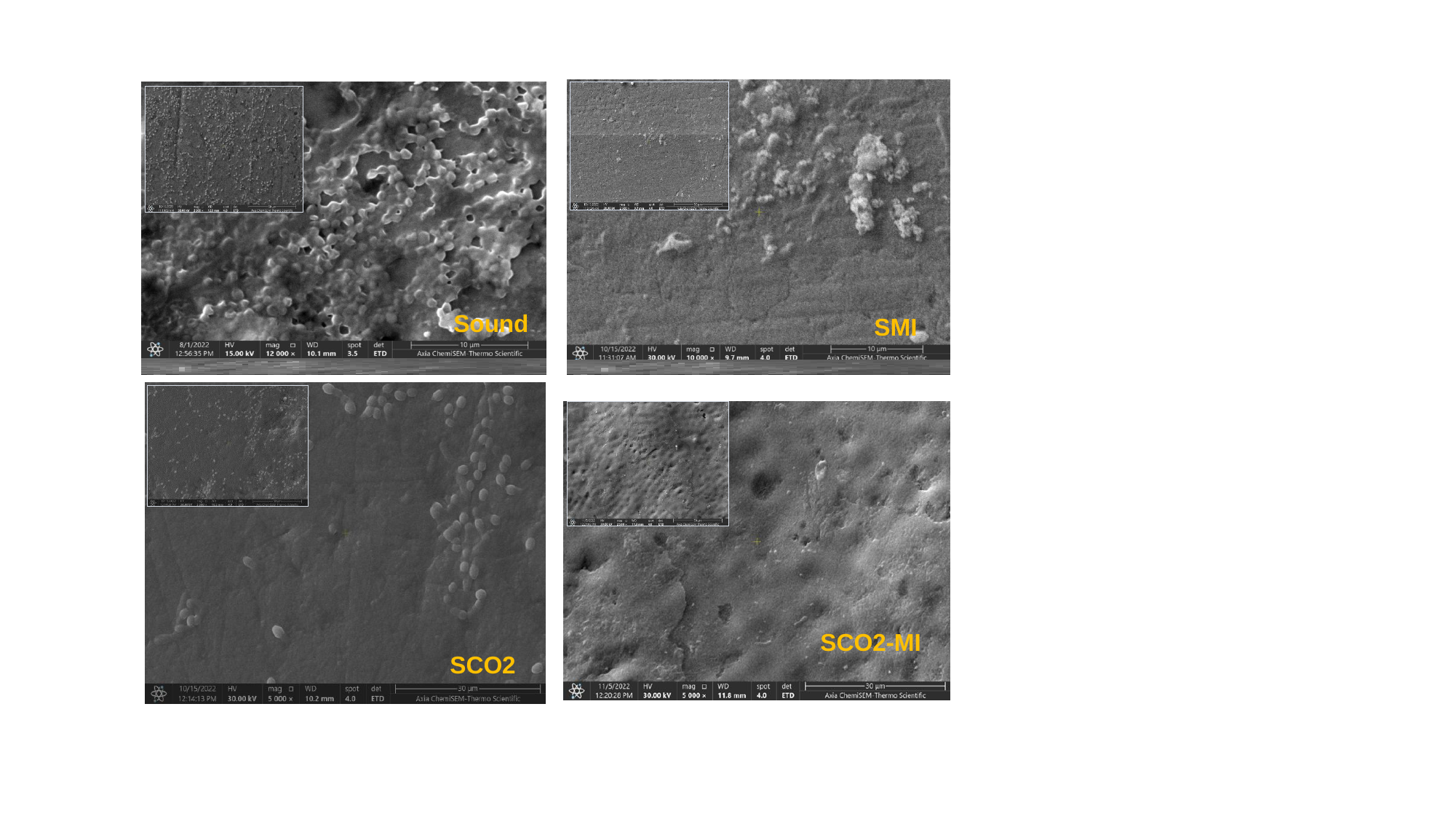

Sound
SMI
SCO2-MI
SCO2

## Slide 5
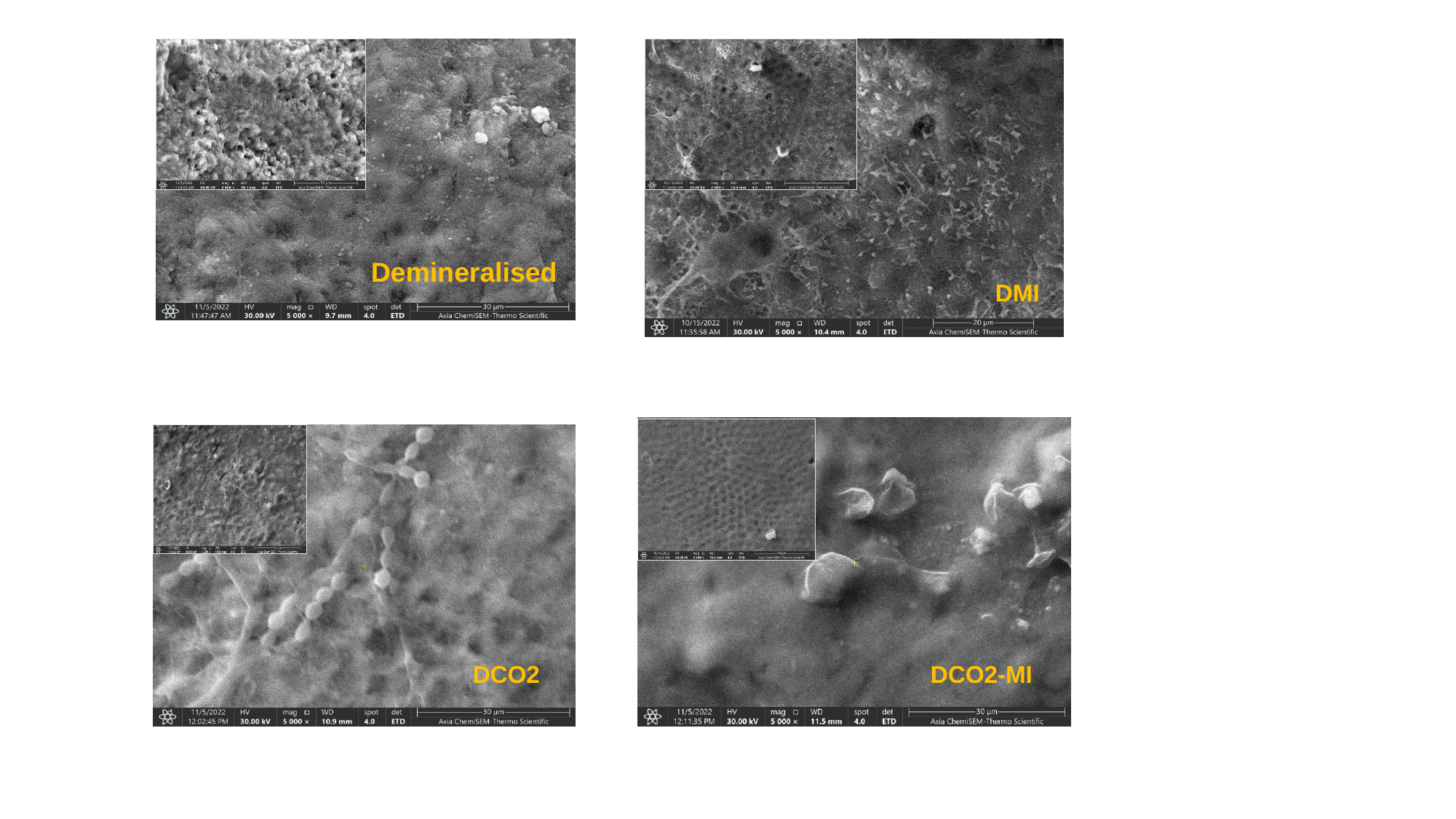

Demineralised
DMI
DCO2
DCO2-MI

## Slide 6
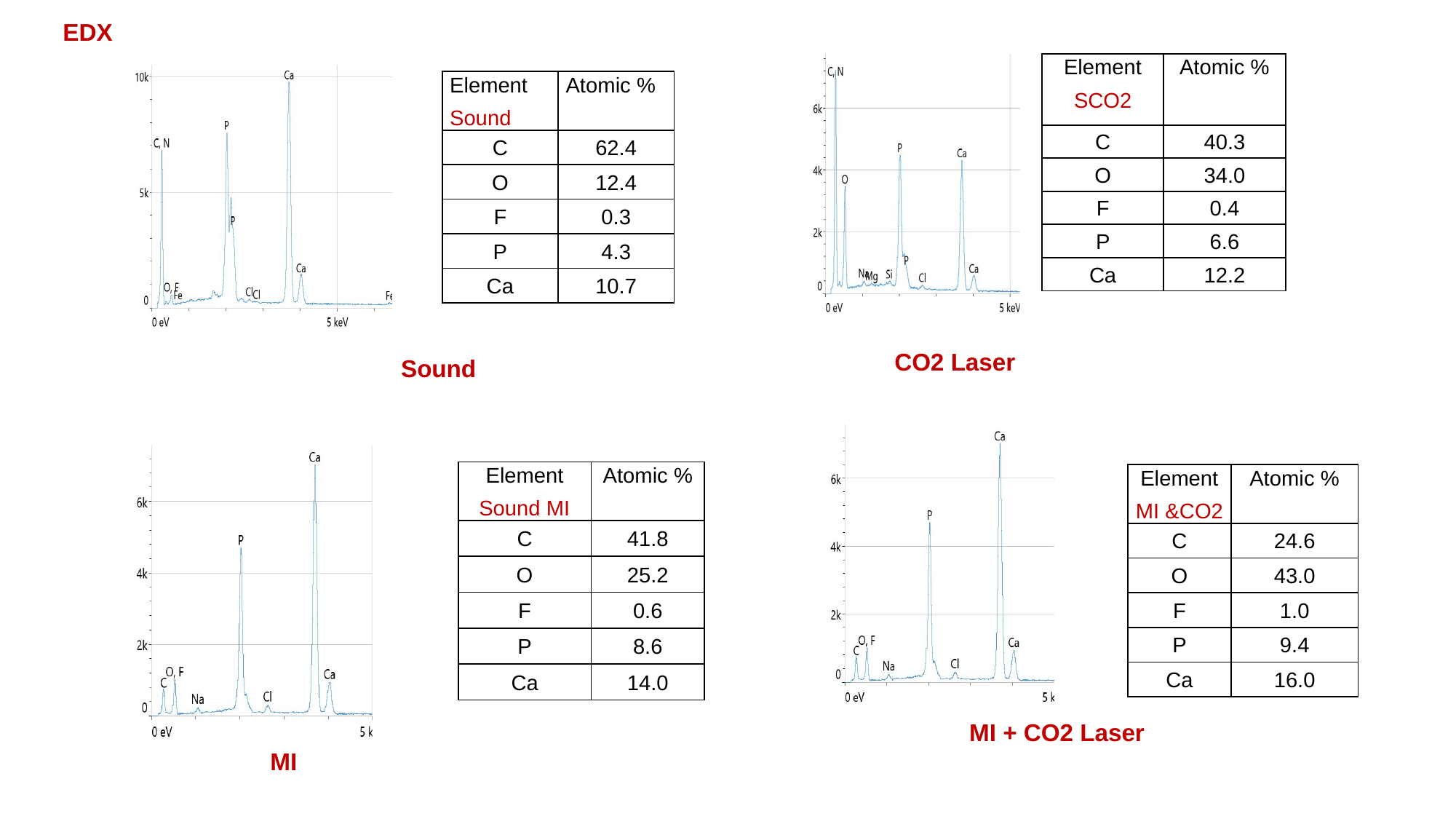

EDX
| Element SCO2 | Atomic % |
| --- | --- |
| C | 40.3 |
| O | 34.0 |
| F | 0.4 |
| P | 6.6 |
| Ca | 12.2 |
| Element Sound | Atomic % |
| --- | --- |
| C | 62.4 |
| O | 12.4 |
| F | 0.3 |
| P | 4.3 |
| Ca | 10.7 |
CO2 Laser
Sound
| Element Sound MI | Atomic % |
| --- | --- |
| C | 41.8 |
| O | 25.2 |
| F | 0.6 |
| P | 8.6 |
| Ca | 14.0 |
| Element MI &CO2 | Atomic % |
| --- | --- |
| C | 24.6 |
| O | 43.0 |
| F | 1.0 |
| P | 9.4 |
| Ca | 16.0 |
MI + CO2 Laser
MI

## Slide 7
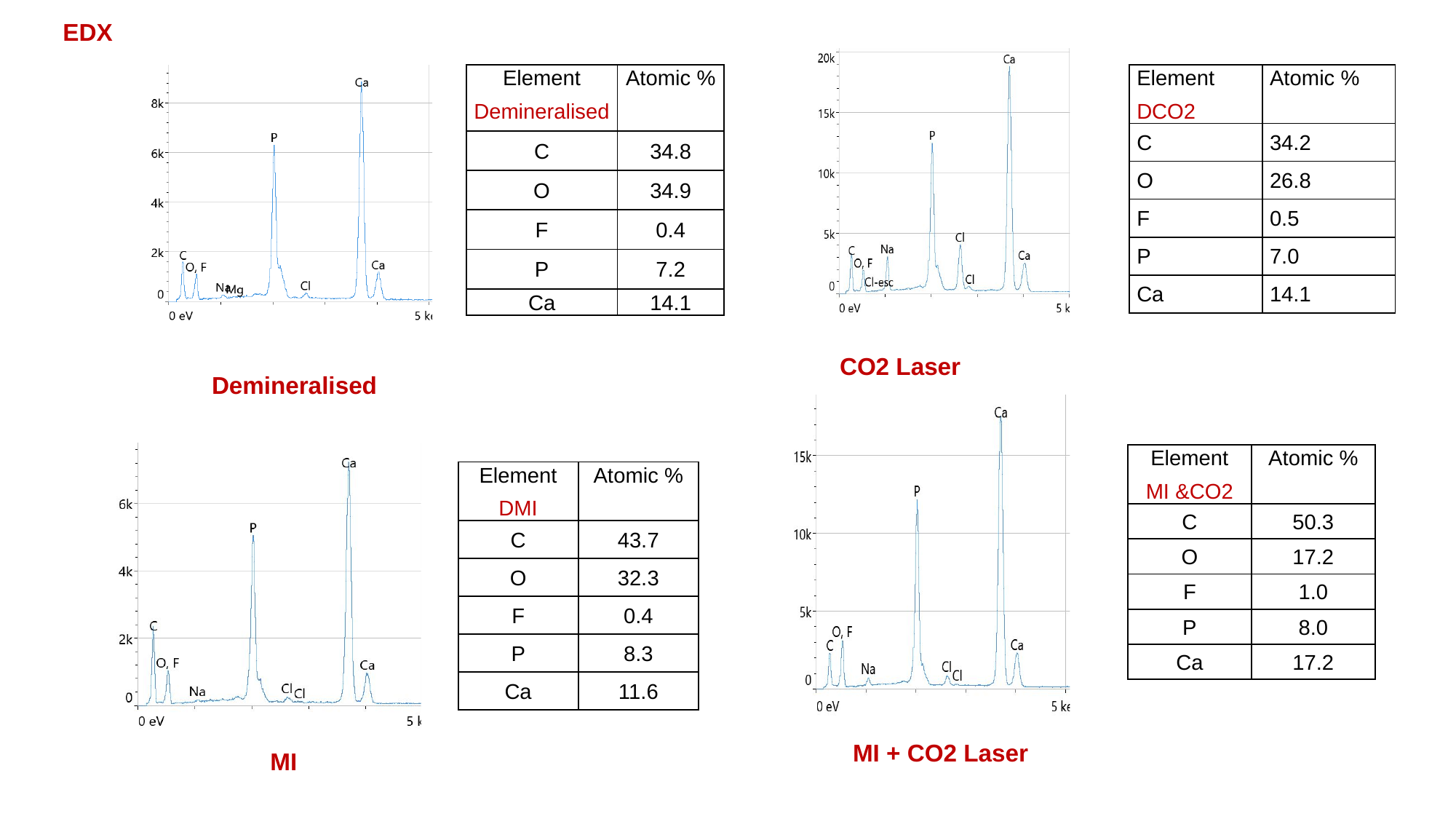

EDX
| Element DCO2 | Atomic % |
| --- | --- |
| C | 34.2 |
| O | 26.8 |
| F | 0.5 |
| P | 7.0 |
| Ca | 14.1 |
| Element Demineralised | Atomic % |
| --- | --- |
| C | 34.8 |
| O | 34.9 |
| F | 0.4 |
| P | 7.2 |
| Ca | 14.1 |
CO2 Laser
Demineralised
| Element MI &CO2 | Atomic % |
| --- | --- |
| C | 50.3 |
| O | 17.2 |
| F | 1.0 |
| P | 8.0 |
| Ca | 17.2 |
| Element DMI | Atomic % |
| --- | --- |
| C | 43.7 |
| O | 32.3 |
| F | 0.4 |
| P | 8.3 |
| Ca | 11.6 |
MI + CO2 Laser
MI

## Slide 8
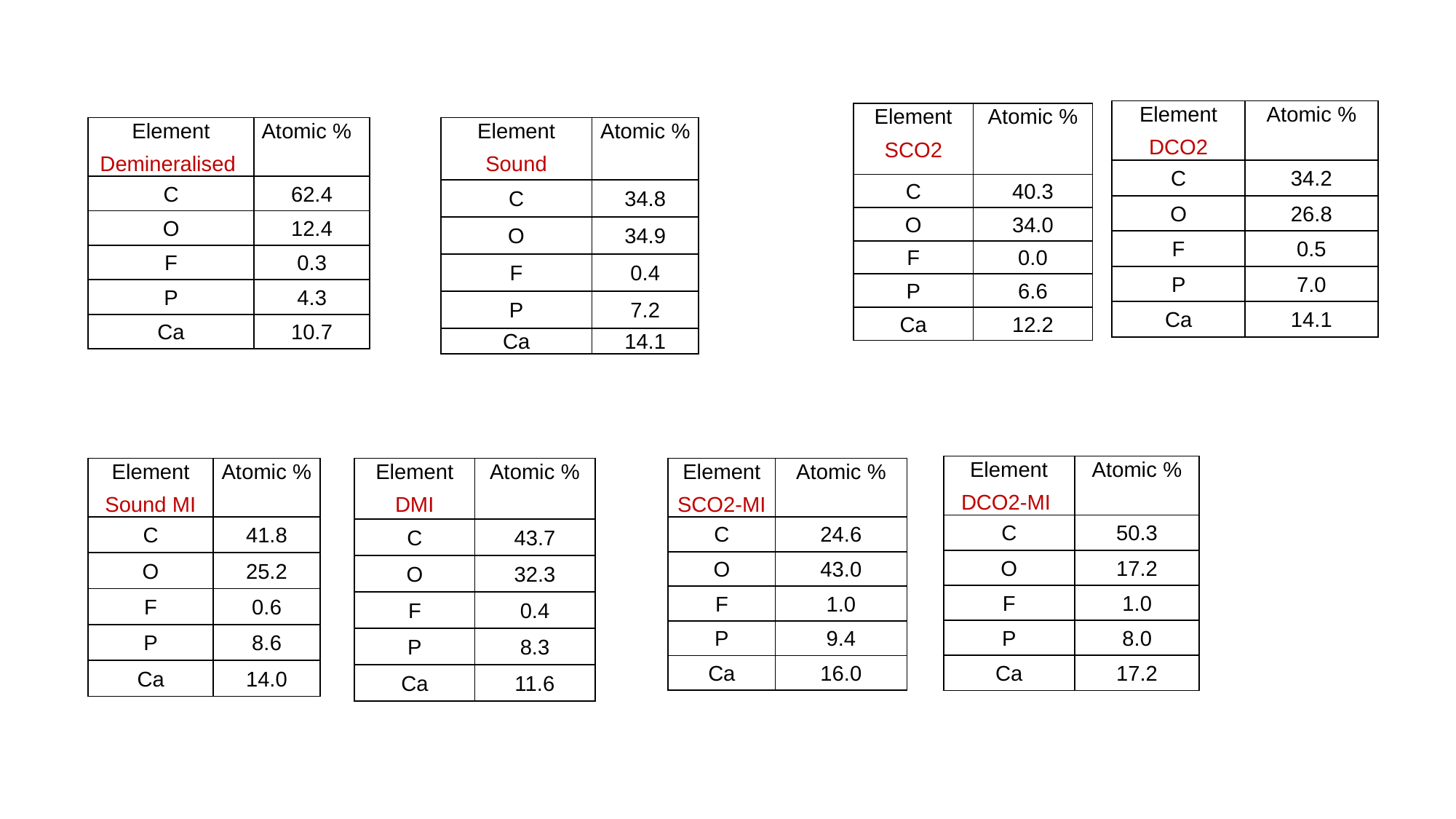

| Element DCO2 | Atomic % |
| --- | --- |
| C | 34.2 |
| O | 26.8 |
| F | 0.5 |
| P | 7.0 |
| Ca | 14.1 |
| Element SCO2 | Atomic % |
| --- | --- |
| C | 40.3 |
| O | 34.0 |
| F | 0.0 |
| P | 6.6 |
| Ca | 12.2 |
| Element Demineralised | Atomic % |
| --- | --- |
| C | 62.4 |
| O | 12.4 |
| F | 0.3 |
| P | 4.3 |
| Ca | 10.7 |
| Element Sound | Atomic % |
| --- | --- |
| C | 34.8 |
| O | 34.9 |
| F | 0.4 |
| P | 7.2 |
| Ca | 14.1 |
| Element DCO2-MI | Atomic % |
| --- | --- |
| C | 50.3 |
| O | 17.2 |
| F | 1.0 |
| P | 8.0 |
| Ca | 17.2 |
| Element Sound MI | Atomic % |
| --- | --- |
| C | 41.8 |
| O | 25.2 |
| F | 0.6 |
| P | 8.6 |
| Ca | 14.0 |
| Element DMI | Atomic % |
| --- | --- |
| C | 43.7 |
| O | 32.3 |
| F | 0.4 |
| P | 8.3 |
| Ca | 11.6 |
| Element SCO2-MI | Atomic % |
| --- | --- |
| C | 24.6 |
| O | 43.0 |
| F | 1.0 |
| P | 9.4 |
| Ca | 16.0 |

## Slide 9
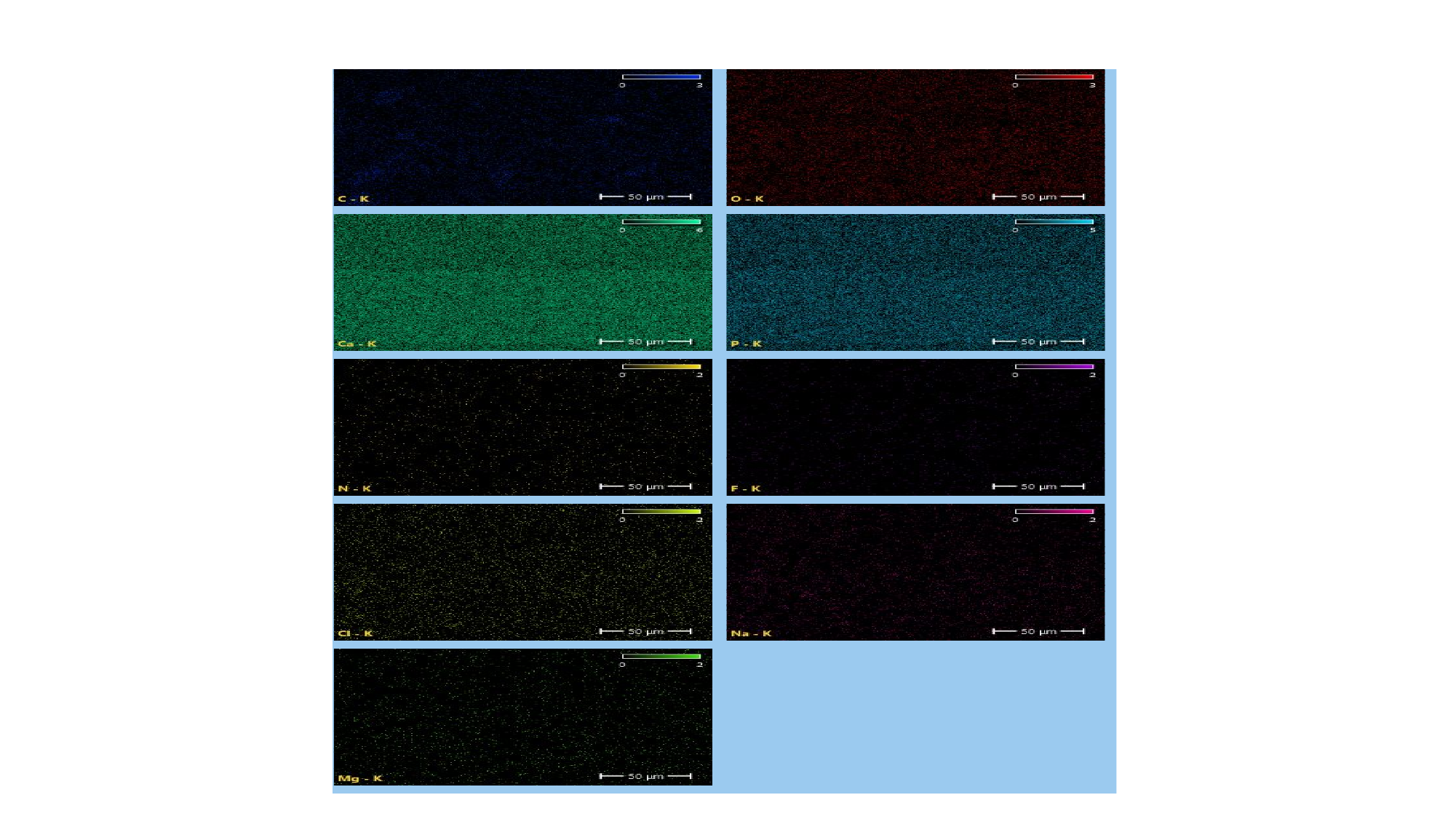

## Slide 10
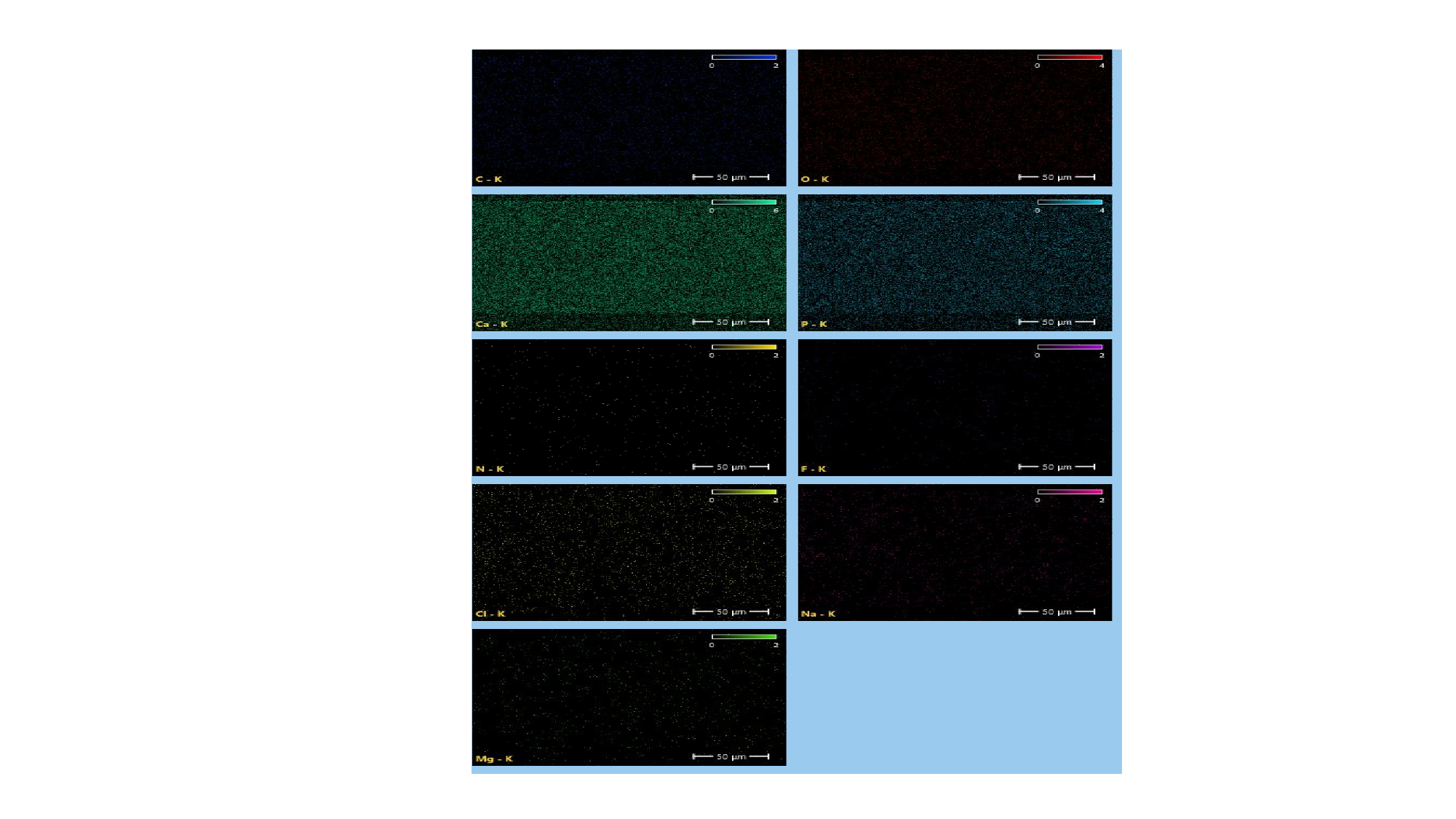

Supplement: Multimedia component 9 [file mmc9.pptx]
